# Association between glycated hemoglobin variability and risk of diabetic kidney disease and diabetic retinopathy in diabetic patients: a systematic review and meta-analysis

**DOI:** 10.3389/fendo.2026.1703190

**Published:** 2026-01-30

**Authors:** Chan Wu, Hanrong Qin, Maoying Wei, Aijing Li, Qingyi Zhu, Jingyi Guo, Anning Sun, Xin Gu, Yincheng Li, Jun Zhang, Yanbing Gong

**Affiliations:** 1 Dongzhimen Hospital, Beijing University of Chinese Medicine, Beijing, China; 2 Ordos City Hospital of Traditional Chinese Medicine, Ordos, China

**Keywords:** diabetic nephropathy, diabetic retinopathy, HbA1c variability, meta-analysis, micro-vascular complications

## Abstract

**Objective:**

To provide a scientific basis for the early prevention of diabetic kidney disease and diabetic retinopathy progression in diabetic patients by systematically evaluating the relationship between glycated hemoglobin (HbA1c) variability and diabetic kidney disease and diabetic retinopathy in these patients.

**Methods:**

Databases including PubMed, Web of Science, Cochrane Library, and Embase were searched for studies investigating the association between HbA1c variability and adverse renal events or retinal diseases in diabetic patients, with data collected from the establishment of each database up to August 5, 2025. Two researchers independently conducted literature screening, data extraction, and assessment of the risk of bias in the included studies. Meta-analysis was performed using the Review Manager 5.3 software, with odds ratio (OR) or hazard ratio (HR) as the effect size indicators.

**Results:**

A total of 45 cohort studies were included in this study, covering 172,111 participants from 20 countries and regions, of which 22 focused on diabetic kidney events and eight on diabetic retinopathy events, and 15 included both outcomes. For the meta-analysis of the association between HbA1c variability and adverse renal events, the standard deviation (SD) of HbA1c was associated with the risk of adverse renal events in patients with type 1 diabetes mellitus (T1DM), with an HR of 0.97 [95% confidence interval (CI): 0.64–1.48, p = 0.90] and an OR of 1.76 (95% CI: 1.12–2.77, p = 0.01); additionally, for each 1% increase in HbA1c-SD, the incidence of adverse renal events in T1DM patients increased, with an HR of 1.40 (95% CI: 1.23–1.59, p< 0.00001). In patients with type 2 diabetes mellitus (T2DM), the coefficient of variation (CV), SD, and high HbA1c variability score (HVS) of HbA1c were all associated with the mortality of adverse renal events, and all HbA1c variability indicators [CV, CV-per 1% increase, SD, SD-per 1% increase, hemoglobin glycation index (HGI), and HVS] were associated with an increased risk of adverse renal events in this population. For the meta-analysis of the association between HbA1c variability and retinopathy, HbA1c-CV was associated with the risk of retinopathy in T1DM patients, with an HR of 1.15 (95% CI: 1.08–1.22, p< 0.0001); HbA1c-SD was also significantly associated with the risk of retinopathy in T1DM, with an HR of 1.83 (95% CI: 1.28–1.63, p = 0.001) and an OR of 4.89 (95% CI: 1.64–14.65, p = 0.005); in T2DM patients, both HbA1c-CV and SD were significantly associated with the risk of retinopathy, with HRs of 1.12 (95% CI: 1.07–1.17, p< 0.00001) and 1.19 (95% CI: 1.06–1.34, p = 0.003), respectively.

**Conclusion:**

HbA1c variability is positively associated with the risks of adverse renal events and retinal diseases in diabetic patients. Therefore, HbA1c variability may play an important and promising role in guiding blood glucose control targets for diabetic patients and predicting the progression of adverse renal events or retinal diseases.

**Systematic Review Registration:**

https://www.crd.york.ac.uk/prospero/, identifier CRD420251133099.

## 1 Introduction

Diabetes mellitus (DM) has emerged as one of the most severe and prevalent chronic diseases of our time, leading to life-threatening, disabling, and costly complications while shortening life expectancy (1). According to the estimates from the 10th Edition of the Diabetes Atlas released by the International Diabetes Federation (IDF) (2), there were 537 million people living with diabetes worldwide in 2021. It is projected that by 2045, the absolute number of people with diabetes will increase by more than 46%, resulting in irreversible damage to human health. According to the data from the *White Paper on Diabetic Complications Research* (3), the proportion of diabetic patients with complications is as high as 61.7%, among which diabetic cardiovascular disease, diabetic nephropathy, diabetic retinopathy, and diabetic foot are the most common (4). Notably, diabetic microangiopathy is the earliest-onset and most prevalent complication of diabetes. Its typical features include impaired microvascular endothelial function, thickened basement membrane, and microthrombus formation—this pathological process further exacerbates damage to patients’ kidneys, eyes, and peripheral nervous system. Existing studies have shown that although hyperglycemia-induced damage to the cardiovascular, cerebrovascular, and other macrovascular systems is the main cause of death in diabetic patients, microangiopathy is more widespread in its harm and exerts a more significant impact on patients’ quality of life (5). Diabetic kidney disease (DKD), as one of the main causes of chronic kidney disease (CKD) and end-stage renal disease (ESRD), has core pathological mechanisms including renal tubular fibrosis, mesangial hypertrophy and expansion, inflammatory cell infiltration, extracellular matrix (ECM) accumulation, and podocyte autophagy. Moreover, patients with DKD are often complicated by diabetic retinopathy (DR). DR develops because persistent hyperglycemia disrupts the homeostatic regulatory mechanisms of the body’s microenvironment. Retinal microvascular endothelial cells trigger the breakdown of the blood–retinal barrier, vascular endothelial dysfunction, increased vascular permeability, and microvascular occlusion through a series of intracellular events, ultimately leading to the onset of the disease (6–8). Based on this, the present study focuses on DKD and DR as the core research objects, aiming to explore the pathogenesis and related rules of diabetic microangiopathy in greater depth and provide a theoretical reference for clinical prevention and treatment.

Glycated hemoglobin (HbA1c) is a product formed by the non-enzymatic binding of glucose to the N-terminal valine of the β-chain of hemoglobin in red blood cells, accounting for approximately 60% to 70% of total hemoglobin (9). As a core indicator for evaluating long-term average blood glucose levels, elevated HbA1c is commonly observed in patients with diabetes and individuals in the prediabetic stage. Given that the lifespan of red blood cells is approximately 120 days, HbA1c can stably reflect the average blood glucose (BG) level over the past 2 to 3 months, without being interfered with by transient increases or decreases in single blood glucose measurements. Meanwhile, its test results show no significant correlation with blood sampling time, insulin (Ins) use, or fasting status. Therefore, HbA1c holds irreplaceable clinical value in the overall condition assessment of diabetic patients. A large body of studies has confirmed that reducing HbA1c levels can significantly decrease the risk of developing microvascular complications, such as DKD and DR, or delay the onset of these complications (10–13). Furthermore, there is sufficient evidence indicating that diabetic patients with comorbid CKD who have poor HbA1c control will face a significantly increased risk of all-cause mortality (14). However, in clinical practice, it has been found that the traditional model of blood glucose management relying solely on HbA1c has obvious limitations, namely, its inability to reflect the fluctuating characteristics of long-term blood glucose. It should be noted that HbA1c is less affected by short-term factors such as diet, medication, and mood, giving it significant advantages over fasting plasma glucose (FPG). Additionally, some studies suggest that when HbA1c is used alone for diabetes diagnosis, the detected prevalence rate is higher than that obtained when fasting plasma glucose is used alone for diagnosis (15, 16).

Previous studies (17, 18) have confirmed that HbA1c variability indicators can effectively predict the blood glucose control efficacy, the risk of microalbuminuria, and the progression trend of kidney disease in diabetic patients. However, within the current body of evidence, there remains a lack of clear conclusions regarding the association between HbA1c variability and DR, and critical gaps persist in the research data on the correlation between these two factors. In terms of study population coverage, previous meta-analyses on the association between HbA1c variability and diabetic microvascular complications have obvious limitations: most studies only included patients with type 2 diabetes mellitus (T2DM), while relevant research on patients with type 1 diabetes mellitus (T1DM) remains extremely limited. Although some studies have focused on the impact of early blood glucose control on long-term complications in patients with childhood-onset T1DM and found that HbA1c levels exhibit a “tracking effect” from the initial diagnosis stage and are associated with the risk of long-term vascular complications, these studies did not conduct an in-depth analysis of the specific association pattern between HbA1c variability and complications (19, 20). Thus, they fail to fill the research gap regarding HbA1c variability in the T1DM population (21–24). At the level of assessment indicators, a variety of quantification methods for HbA1c variability have been developed, including standard deviation (SD), coefficient of variation (CV), HbA1c variability score (HVS), and hemoglobin glycation index (HGI) (25). However, there is a lack of consistency in the application of these indicators across existing studies. Some studies (22) have only verified the association between SD and CV with kidney disease and peripheral neuropathy, while the association between emerging indicators such as HVS and HGI with microvascular complications, especially retinopathy and neuropathy, has not yet been systematically verified. Additionally, differences in the predictive efficacy of different indicators remain unclear. This research status makes it difficult to identify the optimal HbA1c variability assessment indicator in clinical practice, thereby limiting its application in the risk stratification of complications.

Therefore, this meta-analysis aims to systematically address the following key issues: to clarify the strength of the association between different HbA1c variability indicators and the occurrence and progression of DKD and DR in diabetic patients, and to compare the differences in the predictive value of HbA1c variability for complication risk between patients with T1DM and T2DM, thereby providing a more precise theoretical basis for clinical blood glucose management and complication prevention and control.

## 2 Research design and methods

### 2.1 Protocol and registration

This study protocol has been registered in advance in the International Prospective Register of Systematic Reviews (26) (PROSPERO, registration number: CRD420251133099). This meta-analysis was conducted strictly in accordance with the guidelines of the Preferred Reporting Items for Systematic Reviews and Meta-Analyses (PRISMA) statement. Additionally, since all included studies are cohort studies (observational studies), they also adhered to the guidelines of the Meta-Analysis of Observational Studies in Epidemiology (MOOSE) (27).

### 2.2 Search strategy

A comprehensive search was conducted across English databases, including PubMed, Embase, Web of Science, and Cochrane Library, with no language restrictions applied. The search covered the period from the inception of each database up to August 5, 2025. For the search strategy, Medical Subject Headings (MeSH) terms (28) were combined with text words related to HbA1c variability and microangiopathy progression. The search terms include the following: 1) Glycated Hemoglobin, Glycated Hemoglobin A1c, HbA1c, HbA (1c) variability, and HbA (1c) variation; 2) Kidney Diseases, Renal Disease, Proteinuria, Albuminuria, Nephropath, Glomerulosclerosis, Kimmelstiel-Wilson Syndrome, Renal Insufficiency, and Kidney Insufficiency; 3) Retinopathy, Retinal Diseases, and Diabetic Retinopathies; and 4) Diabetes Mellitus, Diabetes Insipidus, Diet, Diabetic, Prediabetic State, Scleredema Adultorum, Glucose Intolerance, Gastroparesis, and Glycation End Products. To supplement the collection of unpublished study results, an additional search was conducted on the ClinicalTrials.gov registry (website: www.clinicaltrials.gov). Meanwhile, by searching gray literature (including unpublished dissertations, conference proceedings, research reports, etc.) and manually reviewing the reference lists of included studies, the scope of literature collection was further expanded to reduce literature omission. During the literature screening stage, two reviewers (C.W and A.J.L) independently completed the initial screening of all literature titles and abstracts. For literature that was deemed potentially eligible for inclusion after the initial screening, full texts were obtained for secondary screening. If the two reviewers have disagreements during the screening process, the disputes will be resolved through discussion and negotiation. If no consensus can be reached through negotiation, a third researcher (Q.Y.Z) will be consulted to determine the final screening result. All retrieved literature will be managed using the EndNote X20 software.

### 2.3 Selection of studies (PICOS)

P: Inclusion criteria were as follows: 1) studies investigating HbA1c variability indicators (including SD, CV, HVS, and HGI); 2) adult patients (aged ≥18 years) with a confirmed diagnosis of diabetes mellitus; 3) studies that included patients without DKD or DR at baseline; and 4) studies from which hazard ratios (HRs), relative risks (RRs), or odds ratios (ORs) and their 95% confidence intervals (CIs) can be extracted. The full texts of potentially relevant studies were downloaded and reviewed for inclusion. Exclusion criteria were as follows: 1) patients with gestational diabetes mellitus, those with diabetes-related renal function decline or retinopathy at baseline, those with a life expectancy shorter than the follow-up period, or those with an insufficient number of HbA1c measurements during the follow-up period; 2) reviews, case reports, practice guidelines, commentaries, *in vitro* or animal studies, analyses after randomized controlled trials, or analyses unrelated to the current research topic; 3) non-English articles; 4) duplicate articles; if the same literature is identified, only one article will be included; and 5) articles from which full texts cannot be obtained, no relevant valid data can be extracted, or there are obvious errors in the data.

I: High levels of HbA1c variability. SD, adjusted standard deviation (Adj-SD), and per 1% increase in SD; coefficient of variation (CV = SD/Mean) and per 1% increase in CV; HVS: HbA1c variability score; HGI: hemoglobin glycation index.

C: The control group consisted of a patient population with low HbA1c variability. Studies typically compared the risk differences between the highest quartile group and the lowest quartile group. Comparison condition: logistic or Cox regression analysis for outcome risk prediction.

O: Occurrence of diabetes-related microangiopathy. Primary outcome: diabetes-related microangiopathy (mainly including diabetic kidney disease and diabetic retinopathy). Secondary outcome: diabetes-related microvascular mortality.

S: Prospective cohort studies or retrospective cohort studies.

### 2.4 Quality assessment

The risk of bias assessment was also independently conducted by two researchers (C.W and A.J.L). For the included cohort studies and subsequent analyses, the Newcastle–Ottawa Scale (NOS) was used to evaluate the study quality in accordance with the recommended standards of the Cochrane Collaboration. This scale uses a maximum 9-star rating system and conducts assessments from three specific dimensions: selection of participants (rating range, 0–4 stars), comparability of study groups (rating range, 0–2 stars), and determination of outcome indicators (the original expression “decision to withdraw” has been optimized; rating range, 0–3 stars). Based on the final rating results, the risk of bias of the included studies is categorized into three levels: studies with a rating of ≥8 stars are defined as low risk of bias, those with a rating of 6–7 stars as moderate risk of bias, and those with a rating of ≤5 stars as high risk of bias.

### 2.5 Data analysis and synthesis

The meta-analysis was performed using Review Manager (RevMan) Version 5.3. Stratified analyses were conducted based on variations in data regarding HbA1c variability indicators (CV, SD, HVS, and HGI) and effect size types (HR or OR) across the included studies. The results of subgroup analyses and pooled values were presented separately. Given the methodological differences between HR and OR, independent analyses were performed for each. A random-effects model was used for data pooling.

Results were visualized as forest plots using the inverse variance method. Data were entered into RevMan as the natural logarithm of HR or OR and their corresponding standard errors. When necessary, the standard error was derived from the CI using the following formula: (ln upper limit of CI − ln lower limit of CI)/(2 × 1.96). The I^2^ statistic was calculated using a random-effects model to assess heterogeneity, with the following criteria: 0%–25% indicating very low heterogeneity, 25%–50% indicating low heterogeneity, 50%–75% indicating moderate heterogeneity, and >75% indicating high heterogeneity. Subgroup analyses were conducted based on dimensions including HbA1c variability indicators, sample size, region, study design, follow-up duration for HbA1c variability, and comparison level of HbA1c variability to identify the sources of heterogeneity. Sensitivity analyses were performed to assess the robustness of the results by excluding low-quality studies, removing studies that only reported RRs, excluding studies with short average follow-up duration or unclear follow-up duration, and re-analyzing using a fixed-effects model. Publication bias was evaluated using Egger’s test and funnel plots. If publication bias existed, the trim-and-fill method was used to estimate the impact of missing studies. A p-value<0.05 was considered the threshold for statistical significance in all analyses.

### 2.6 Clinical definitions

SD was calculated as $\sqrt{\Sigma_{k=1}^{n}\frac{{\left(x_{i}-\bar{x}\right)}^{2}}{n-1}}$, and adjusted SD was calculated as $SD/\sqrt{\frac{n}{n-1}}\cdot CV$ was calculated as $SD/\bar{X}$, and the adjusted CV was calculated as $CV/\sqrt{\frac{n}{n-1}}$, where n = total number of HbA1c measurements, $X_{i}=$ serially measured HbA1c, and $\bar{X}=$ mean of HbA1c. HVS was the number of HbA1c changes >0.5% over the total number of HbA1c measurements. HGI was calculated as measured HbA1c minus predicted HbA1c from fasting blood glucose (FBG) levels.

SD is the most commonly used indicator for HbA1c variability, which reflects the degree of dispersion of HbA1c test results around the mean value. CV is a relative variability index derived from the standardization of SD against the mean HbA1c level; it eliminates the impact of mean values on outcome evaluation, thereby enabling horizontal comparison of variability across different populations or studies. HVS can directly reflect the fluctuation frequency of HbA1c, yielding more intuitive results that are better aligned with the practical needs of clinical management (25). HGI reflects the discrepancy between the actual glycation level and the glycation level predicted by fasting blood glucose. HGI often indicates the influence of non-glycemic factors on HbA1c, including biological differences such as interindividual red blood cell lifespan and glycation rate, and thus can reduce the individual variability of HbA1c (29). For a detailed comparison of these indicators, refer to **Appendix C Table 1**.

The diagnostic criteria for T1DM were as follows (30): 1) FBG ≥ 7.0 mmol/L, 2) 2-h oral glucose tolerance test (OGTT) glucose level or casual plasma glucose level ≥11.1 mmol/L (accompanied by typical symptoms of diabetes such as polyuria, polydipsia, polyphagia, and rapid weight loss, or diabetic ketoacidosis), 3) HbA1c ≥ 6.5% (detected by a method certified and traceable to international standard, 4) positive islet autoantibodies [including at least one of glutamic acid decarboxylase antibody (GAD-Ab), islet cell antibody (ICA), insulin autoantibody (IAA), and zinc transporter 8 antibody (ZnT8-Ab)], 5) significantly reduced islet function (e.g., fasting C-peptide<0.3 nmol/L and peak C-peptide<0.6 nmol/L during OGTT), and 6) prior diagnosis of T1DM.

The diagnostic criteria for T2DM were as follows (31): 1) FBG ≥ 7.0 mmol/L, 2) 2-h oral glucose tolerance test or casual plasma glucose level ≥11.1 mmol/L, 3) HbA1c ≥ 6.5%, or 4) prior diagnosis of T2DM.

DKD is mainly based on renal function indicators and urinary protein levels. Renal function assessment uses the estimated Glomerular Filtration Rate (eGFR) calculation formulas recommended by Modification of Diet in Renal Disease (MDRD), Chronic Kidney Disease Epidemiology Collaboration (CKD-EPI), or the Japanese Society of Nephrology (JSN). DKD is defined as follows (32–34): eGFR< 60 mL/min/1.73 m^2^, eGFR< 15 mL/min/1.73 m^2^, annual decline rate of eGFR ≥ 5 mL/min/1.73 m^2^, or progression to the renal replacement therapy (RRT) stage. Urinary protein classification is based on the urine albumin-to-creatinine ratio (UACR): normal albuminuria (UACR< 30 mg/g Cr), microalbuminuria (30 ≤ UACR< 300 mg/g Cr), and macroalbuminuria (UACR ≥ 300 mg/g Cr). The term “proteinuria” is a general designation for microalbuminuria or macroalbuminuria.

DR (35, 36) grading is based on fundus examination findings, including the following: mild non-proliferative diabetic retinopathy (NPDR; microaneurysms only), moderate NPDR (microaneurysms accompanied by non-severe intraretinal hemorrhages/hard exudates), severe NPDR [intraretinal hemorrhages in four quadrants, venous beading in two quadrants, and intraretinal microvascular abnormalities (IRMAs) in one quadrant], and proliferative diabetic retinopathy (PDR; presence of neovascularization, vitreous hemorrhage, or preretinal hemorrhage).

## 3 Results

### 3.1 Characteristics of included studies

**Figure 1:**
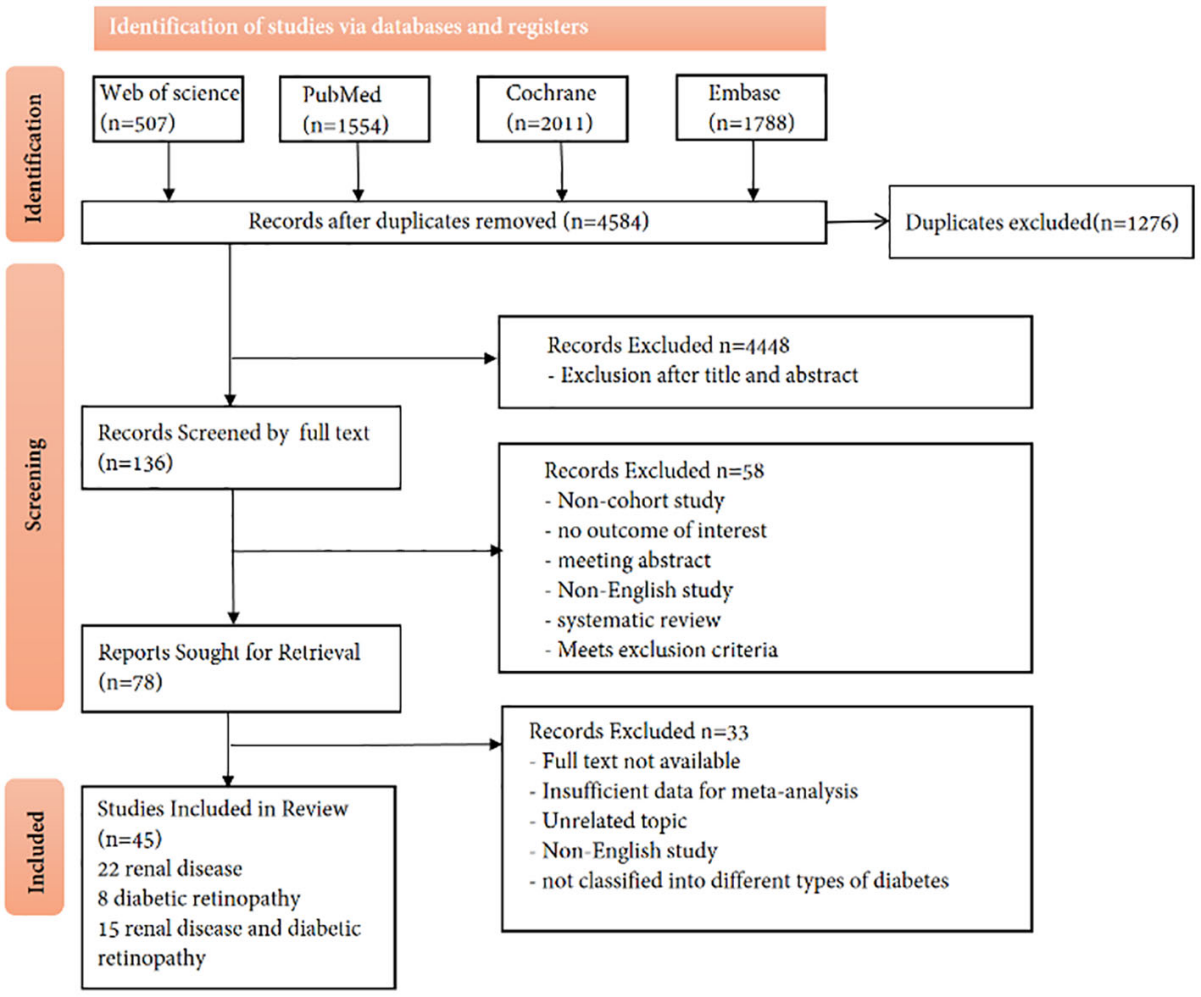
PRISMA flow diagram outlining the selection process that was undertaken for the systematic review and meta-analysis. PRISMA, Preferred Reporting Items for Systematic Reviews and Meta-Analyses.

A total of 4,584 articles were retrieved using the search methods described above. Among these, 1,276 duplicate articles were excluded. After a preliminary review of titles and abstracts, 4,448 articles that did not align with the research topic were excluded, resulting in 136 articles after the initial screening. Subsequently, a detailed full-text review was conducted, and 58 articles were excluded, including non-cohort studies, those without relevant results, conference abstracts, non-English studies, and systematic reviews, leaving 78 articles. Finally, articles that could not be downloaded and had incomplete data were excluded, resulting in 45 articles. The search process is shown in **Figure 1**.

Among the 45 included cohort studies, there were 22 studies focusing on diabetic kidney disease events (18, 37–57), eight studies on diabetic retinopathy events (58–65), and 15 studies (12, 66–79) covering both of these two outcomes. The studies involved 172,111 participants from 20 countries and regions. Among them, a larger number of studies were conducted in Europe and Asia, while only one study (50) was from Africa. Regarding the study population, 31 studies (12, 18, 39–41, 43, 44, 46–49, 52–56, 59, 62–65, 67, 68, 70–72, 74–77, 79) included patients with T2DM, 13 studies (37, 38, 42, 45, 50, 57, 58, 60, 61, 66, 69, 73, 78) included patients with T1DM, and one study (51) did not specify the type of diabetes.

For the outcome of diabetic kidney disease, a total of 37 studies (12, 18, 37–57, 66–79) reported data on a total sample size of 123,722 participants from 18 countries and regions, with the sample size ranging from 201 to 40,622. Among these studies, 10 focused on patients with type 1 diabetes mellitus, 26 on patients with type 2 diabetes mellitus, and one did not specify the type of diabetes mellitus. Twenty-five studies reported SD values (19 reported original SD values, and six reported adjusted SD values), 20 studies reported CV values, two studies reported HGI values, and five studies reported HVS values. Additionally, 26 studies reported HRs, and 11 studies reported ORs. The average HbA1c level ranged from 8.6% to 11.0%, and the follow-up duration ranged from 3 to 16.4 years.

For the outcome of diabetic retinopathy, a total of 23 studies (12, 58–79) reported data on a total sample size of 109,193 participants from 14 countries and regions, with the sample size ranging from 201 to 40,622. Among these studies, seven focused on patients with type 1 diabetes mellitus and 16 on patients with type 2 diabetes mellitus. Thirteen studies reported SD values (10 reported original SD values, and three reported adjusted SD values), and 15 studies reported CV values. Additionally, 15 studies reported HRs, and seven studies reported ORs. The average HbA1c level ranged from 4.3% to 8.8%, and the follow-up duration ranged from 3 to 28 years.

Meanwhile, the NOS was used to evaluate the quality of the included literature. Among the 45 articles, the NOS scores ranged from 6 to 8. A total of 15 articles (38, 42, 45, 46, 50, 61, 64–67, 69, 70, 72, 73, 77) scored 6, 21 articles (12, 18, 37, 39, 41, 44, 47–49, 51, 54, 56–60, 62, 63, 71, 75, 76, 78) scored 7, and eight articles (40, 43, 52, 53, 55, 68, 74, 79) scored 8. All articles were classified as having low to moderate risk of bias. The NOS scores of the included articles are presented in **Tables 1**, **2**.

**Table 1:** Characteristics of the studies considered in the meta-analysis(DN).

Study(Author, year)	Design(Type of study)	Number(male%)	Age at enrolment	Area (follow-up time, years)	Inclusion criteria	HbA1c variability and follow-up time	Mean HbA1c(%)	Outcome	Variable adjustment	NOS score
Wadén J et al.(2009) (37)	observational cohort study	2107(53.2%)	36.4±11.8	Finland(median follow-up period of 5.7 years)	patients with T1DM	SD:NA, median number of HbA1c measurements:13(7-20), measurements per year:2.3	8.5±1.5	progression in renal status	Adjusted for duration of diabetes, sex, SBP, TC, ever smoking, intrapersonal mean of serial HbA1c measurements, and number of HbA1c measurements	7
Marcovecchio ML et al.(2011) (38)	observational cohort study	1232(55.44%)	9.2(5.7-11.7)	UK(NA)	418: children with T1DM 814: adolescents with T1DM	SD:1.05, NA	9.5	microalbuminuria	Adjusted for sex, age at diagnosis, and chronologic age	6
Sugawara A et al.(2012) (41)	observational cohort study	812(68.72%)	54.9±10.4	Japan(4.3±2.7 years)	patients with T2DM	SD:NA, NA	11.0(5-12)	the development of microalbuminuria	Adjusted for age, sex, duration of diabetes, SBP, BMI, TC, HDL-C, ever smoker	7
Hsu CC et al.(2012) (39)	prospective cohort study	821(46.1%)	NA	Taiwan, China(6.2±0.7 years)	patients with T2DM	Adjusted SD:1.03±0.51, number of HbA1c measurements: 9.0±2.7	8.2±1.8	progression to microalbuminuria	Adjusted for age at diabetes onset, sex, education, diabetes duration, smoking status, waist circumference, triacylglycerol and HDL-C levels, mean HbA1c and BP	7
Rodrı´guez-Segade S et al.(2012) (40)	prospective cohort study	2103(47.7%)	59.2(10.6)	Spain(6.6 years)	patients with T2DM	SD:NA, median number of HbA1c measurements:10(6-14), measurements per patient per year: 1.4	7.5(2.0)	progression of nephropathy	Adjusted for age, duration of diabetes, use of insulin, HbA1c, BMI, retinopathy status, use of anti- hypertensive agents, smoking status, cholesterol and triglyce-Rides, sex, cohort, the number of HbA1c measurements and updated mean HbA1c	8
Luk AO et al.(2013) (18)	prospective cohort study	8439(47.0%)	57.6±13.2	Hong Kong, China(median follow-up period of 7.2 years)	patients with T2DM	Adjusted SD:NA, median number of HbA1c measurements:10(5–17), median frequency of HbA1c measurement per year: 2.0(1.1-2.8)	NA	CKD	Adjusedt for age, gender, smoking history, diabetes duration, BMI, waist circumference, SBP/DBP, LDL-C, HDL-C, log triglyceride, log urine ACR, eGFR, haemoglobin and baseline medication use including the use of ACEI/ARB, antihypertensive drugs, lipid-lowering drugs, oral hypoglycaemic drugs and insulin	7
Nazim J et al.(2014) (42)	prospective cohort study	438(55.02%)	NA	Poland(9.2±3.4 years)	children and adolescents with T1DM	CV:8.86±5.99, at least 4 times a year per person	7.89±1.39	microalbuminuria	NA	6
Yang YF et al.(2015) (43)	observational cohort study	31841(NA)	NA	Taiwan, China(8.23 years)	patients with T2DM	CV:NA, 3-6 months	HbA1c-CV≤5.7: 7.59(1.65), HbA1c-CV 5.7-9.7: 7.76(1.64), HbA1c-CV 9.7-14.9: 8.02(1.68), HbA1c-CV 14.9-24.4: 8.37(1.86), HbA1c-CV>24.4: 9.35(2.41)	ESRD incident, all-cause mortality	Adjusted for age, sex, smoking, alcohol consumption, duration of diabetes, type of hypoglycemic antihypertensive drugs (RAS, BBs, CCBs, diuretics), albuminuria, obesity, coronary artery disease, congestive heart failure, cancer, hyperlipidemia, hypertension, atrial fibrillation, chronic hepatitis, chronic obstructive pulmonary disease, stroke, hypoglycemia, eGFR, SBP, DBP, FG, and HbA1c	8
Low S et al.(2016) (44)	prospective cohort study	967(55.2%)	60.1±11.1	Singapore(median follow-up period of 5.3(3.9–6.9) years)	patients with T2DM and CKD	CV:11.7(8.2–17.5), NA	8.4±2.0	decline in eGFR category , development of albuminuria, occurrence of eGFR 60 ml/min/1.73 m^2^ and/or development of albuminuria, annual rate of eGFR decline N 5ml/min/1.73m^2^/year	NA	6
Raman S et al.(2016) (45)	retrospective cohort study	1195(53%)	14.7±3.5	USA(NA)	children with T1DM	SD:1.67±0.88, number of HbA1c measurements: 14(8-21)	8.8±1.3	microalbuminuria	Adjusted for sex, race/ethnicity, and age at T1DM diagnosis	6
Virk SA et al.(2016) (66)	prospective cohort study	1706(47%)	NA	Canada(median follow-up period of 8.1 years)	patients with T1DM	SD:NA, CV:NA, number of HbA1c measurements: 22(14-29), measurements per patient per year: 2.7	NA	albuminuria, Log10 AER	Adjusted for age, sex, diabetes duration, SBP, DBP, cholesterol , height, BMI, and socioeconomic disadvantage	6
Shen ZZ et al.(2017) (47)	observational cohort study	402(46.52%)	Short term intensive diabetic education: 57.1±11.1, Controls:56.4±12.8	China(10 years)	patients with T2DM	SD:NA, 2-6 months	short term intensive diabetic education: 8.46±1.92, Controls: 9.52±2.10	nephropathy progress	NA	7
LOW S et al.(2017) (46)	retrospective cohort study	1628(57.9%)	NA	Singapore(median follow-up period of 5.5(4.2-7.0) years)	patients with T2DM	CV:10.5(7.3-15.8), number of HbA1c measurements: 8(6-11)	8.0(7.3-9.1)	eGFR decline	Adjusted for age at entry, gender, duration of DM, ethnicity, SBP≥140 mmHg, ln-transformed baseline eGFR, ACR group, LDL-C≥2.6 mmol/L, ln-transformed number of HbA1c measurements, and the use of RAS inhibitor(s).	7
Takao T et al.(2017) (67)	retrospective cohort study	243(78.6%)	55.9±9.3	Japan(NA)	patients with T2DM	CV:NA, NA	8.0±1.7	microalbuminuria	Adjusted for mean HbA1c, mean SBP, number of visits, age, sex, diabetes duration, BMI, TC/HDL-C, baseline smoking status, baseline alcohol intake, baseline use of insulin, and baseline use of ACEI	6
Lee MY et al.(2018) (48)	observational cohort study	388(60.31%)	65.7±10.9	Taiwan, China(median follow-up period of 3.5(0.5-9.3) years)	patients with T2DM	SD:NA, NA	NA	progression to dialysis	Adjusted for age, sex, hypertension, coronary artery disease and cerebrovascular disease, mean HbA1C, triglyceride, TC, baseline eGFR, calcium-phosphorous product, uric acid and ACEI and/or ARB use	7
Teliti M et al.(2018) (49)	observational cohort study	900(57.2%)	66.96±10.00	Italy(NA)	patients with T2DM	SD:0.46±0.36, CV:0.06±0.04 NA	7.0±0.9	nephropathy	Adjusted for age, sex, disease duration, BMI, level of total cholesterol, HDL-C, triglycerides, eGFR value, smoking habit, hypertension, dyslipidemia, presence of macro-vascular events, presence of retinopathy and peripheral neuropathy and treatment with anti-diabetic drugs	7
Cardoso CRL et al.(2018) (68)	prospective cohort study	654(38.1%)	60.1(9.6)	Brazil(median follow-up period: 9.3(5.2-10.8) years)	adults with T2DM	SD:NA, NA	8.1(1.9)	composite renal outcome, microalbuminuria (incident), renal failure	Adjusted for age, sex and number of HbA1c or FG measurements, diabetes duration, BMI, smoking status, physical inactivity, arterial hypertension, number of anti-hypertensive drugs in use, ambulatory 24-h SBP, presence of micro- and macrovascular complications at baseline, serum mean HDL-C and LDL-C, and use of insulin, statins and aspirin, mean FG and ­HbA1c	8
Rosa LCGFD et al.(2019) (69)	retrospective cohort study	220(40%)	29.6±10.1	Brazil(>10 years)	adults with T1DM	Adjust-SD:1.24±0.88, CV:1.38±0.63, NA	8.3±1.5	albuminuria, eGFR<60	Adjusted for age, sex, T1DM duration, presence of hypertension, and mean LDL-C levels	6
Slieker RC et al.(2019) (70)	prospective cohort study	6780(NA)	NA	Netherlands(NA)	patients with T2DM	CV:NA, NA	NA	eGFR stage, CKD stage, microalbuminuria, macroalbuminuria, all–cause mortality	Adjusted for sex, BMI, HDL, age at diagnosis, triglycerides, HbA1c at baseline, oral glucose lowering drugs, insulin use and eGFR	6
Song KH et al.(2019) (70)	retrospective cohort study	604(54.5%)	60.7±10.8	Korea(3 years)	patients with T2DM	SD:NA, 3-6 months	7.32±1.04	the progression of DN	Adjusted for eGFR, triglyceride HDL-C ratio, the presence of DR, and use of an ACEI or ARB	7
Wakasugi S et al.(2021) (74)	prospective cohort study	999(60.9%)	64.6±9.6	Japan(NA)	patients with T2DM	SD:2.04±0.63, CV:2.62±5.79, NA	7.1±0.8	albuminuria severity	Adjusted for age, gender, BMI, and duration of diabetes, SBP, TC, HDL-C, logarithm of triglycerides, serum uric acid, eGFR, smoker, alcohol consumption, presence of DR, use of insulin therapy, use of ACEI and/or ARB, use of statins, and use of antiplatelet agents and HbA1c	8
Bille N et al.(2021) (50)	observational cohort study	471(43.10%)	NA	Rwanda(NA)	patients with T1DM	SD:NA, Adjust-SD:NA, CV:NA, HVS:NA, NA	NA	nephropathy	Adjusted for sex, region, age at diagnosis, disease duration, BMI, SBP, DBP and insulin dosage	6
Lee S et al.(2021) (72)	retrospective cohort study	3137(NA)	NA	Hong Kong, China(10 years)	patients with T2DM	SD:1.1± 0.71, CV:13.6 ± 7.6, HVS:NA, number of HbA1c measurements: 11.9±4.8	8.1±1.8	renal complications, microalbuminuria and macroalbuminuria,proteinuria	NA	6
Romero-Aroca P et al.(2021) (73)	prospective cohort study	366(NA)	NA	Spain(12 years)	patients with T1DM	SD:NA, CV:NA, NA	NA	microalbuminuria	Adjusted for current age, arterial hypertension, eGFR and mean-HbA1c.	6
Afghahi H et al.(2022) (51)	observational cohort study	325(71%)	65.9±13	Swenden(3.0±3.2years)	patients with DM	CV:NA, number of HbA1c measurements: 2-12	6.8±2.4 (median 6.6, range: 4.2-19.7)	all-cause mortality	Adjusted for age, sex, MAP, CRP, serum albumin and CVD	7
Ma C et al.(2022) (75)	observational cohort study	2161(38.45%)	NA	China(NA)	patients with T2DM	Adjust-SD:NA, CV:NA, NA	NA	renal events	Adjusted for gender, age, duration of T2DM, BMI, smoking, baseline concomitant disease, triglycerides, LDL-C, BP, anti-hyperglycemic therapy, and ACEI or ARB treatment, average HbA1c	7
Zhou YL et al.(2022) (54)	retrospective cohort study	2397(59.2%)	58.5(48.9,67.1)	China(median follow-up period of 4.7(3.1-6.3) years	patients with T2DM	HVS: NA, 1.9(1.3, 2.7)years	7.2(6.7,8.3)	a rapid eGFR annual decline	NA	7
Sun B et al.(2022) (76)	retrospective cohort study	855(NA)	NA	China(median follow-up period of 4.8 years)	patients with T2DM	CV:NA, at 3, 6, 12, 18, 24 months, and every 6 months thereafter	NA	new or worsening nephropathy	Adjusted for age, duration of diabetes, gender, BMI, current smoking status, SBP and DBP, TC, triglycerides, HDL-C and LDL-C, baseline use of insulin and mean HbA1c during the first 24 months, history of MACE and microvascular diseases	7
Wu TE et al.(2022) (12)	prospective cohort study	1869(50.4%)	63.2±12.7	Taiwan, China(median follow-up period of 9.5 years)	patients with T2DM	SD:0.728±0.528, number of HbA1c measurements: 19, 10 to 42	8.06±1.77	UACR>300 mg/g, doubling of serum creatinine, all-cause mortality	Adjusted with HbA1c-mean, age, sex, diabetes duration, BP, BMI, TC, HDL-C, triglyceride, and smoking status	7
Lin CH et al.(2022) (52)	retrospective cohort study	780(53%)	60(53-66)	Taiwan,China(median follow-up period of 7.3 years)	patients with T2DM	HGI:NA, NA	7(6.6-7.6)	average eGFR decline rate> 3ml/min/1.73m^2^/year, average eGFR decline rate> 3ml/min/1.73m^2^/year and resultant eGFR< 60 ml/ min/1.73 m^2^, average eGFR decline rate> 5 ml/min/1.73m^2^/year, average eGFR decline rate> 5 ml/min/1.73m^2^/year and resultant eGFR< 60 ml/ min/1.73 m^2^, onset of macroalbuminuria	Adjusted for baseline age, sex, BMI, hypertension, use of RAAS blocker, use of diuretic, eGFR and HbA1c quartiles	8
Yan Y et al.(2022) (53)	retrospective cohort study	699(68.24%)	56.1±10.4	Japan(median follow-up period of 9.9 years)	patients with T2DM	CV:NA, SD:NA, HVS:NA, number of HbA1c measurements:28±10	7.7±1.5	incidence of microalbuminuria, eGFR	Adjusted for age, sex, mean HbA1c over the 3-year period, duration of diabetes, SBP, LDL-C, (eGFR), and presence of ischemic heart disease and heart failure at baseline	8
Ma Y et al.(2023) (77)	retrospective cohort study	387(61.5%)	48.85±9.42	China(median follow-up period of 4.5 years)	patients with T2DM	CV:9.17(6.54,12.55), NA	7.31±1.57	glomerular lesions	NA	6
Suh J et al.(2023) (78)	retrospective cohort study	201(43.8%)	NA	Korea(median follow-up period of 16.4 years)	children and adolescents with T1DM	Adjust-SD:1.04±0.58, 3 months	NA	DKD	Adjusted for age, duration of disease, and sex	7
Zhang F et al.(2023) (55)	retrospective cohort study	820(51.7%)	56.9±14.6	China(median follow-up period of 3.67(2.25,5.83) years)	peritoneal dialysis patients with T2DM	HVS:NA, NA	7.0±2.3	all-cause death	Adjusted for time-weighted average ­HbA1c and age, sex, CVD history, BMI, hemoglobin, albumin and CRP	8
Cardoso CRL et al.(2024) (56)	prospective cohort study	687(38.4%)	60.1±9.5	Brazil(median follow-up period of 10.6 years)	patients with T2DM	HGI:0±1.6, NA	8.0±1.9	new microalbuminuria development or progression to macroalbuminuria, advanced renal failure development	Adjusted for age and sex, BMI , physical activity, smoking status, diabetes duration, pre-existent macrovascular and microvascular complications, SBP, serum LDL-C, use of insulin, aspirin and statins, and number of antihypertensive drugs in use, HGI and HbA1c parameters	7
Muthukumar A et al.(2024) (57)	observational cohort study	3466(50%)	35(26-46)	London,UK(median follow-up period of 8.2(4.2–11.6) years)	patients with T1DM	SD:NA, CV:NA, NA	8.9(4.4)	time to DKD progression	Adjusted for age, gender, IMD deciles, SBP, DBP, Log-10-transformed urinary ACR, ethnicity and baseline HbA1c	7
Teh XR et al.(2025) (79)	retrospective cohort study	40662(38.3%)	57.2(13.9)	Thailand(10 years)	patients with T2DM	SD:0.67(0.87), CV:0.07(0.08), 3-6 months	7.7(2.0)	CKD	Adjusted for age, gender, insurance scheme, BMI, TC, LDL, HDL, triglyceride, haemoglobin, SBP/DBP, hypertension, dyslipidemia, presence of T2DM complications (CVD, DR, or CKD) prior to the outcome of interest, medication use in terms of drug classes (biguanides, sulphonylurea, insulin, alpha- glucosidase inhibitors, DPP-4i, GLP1-RA, TZD, SGLT2i, meglitinides, statins) and the number of antihypertensive drugs	8

DN, diabetic nephropathy; HbA1c, glycated hemoglobin A1C; NOS, Newcastle-Ottawa Scale; T1DM, Type 1 Diabetes Mellitus; SD, standard deviation; SBP, systolic blood pressure; TC, total cholesterol; NA, not available; T2DM, Type 2 Diabetes Mellitus; BMI, mean body mass index; HDL-C, high-density lipoprotein cholesterol; BP, blood pressure; LDL-C, low-density lipoprotein cholesterol; CKD, chronic kidney disease; ACR, albumin/creatinine ratio; eGFR, estimated glomerular filtration rate; DBP, diastolic blood pressure; ACEI, angiotensin-converting enzyme inhibitor; ARB, angiotensin-converting enzyme receptor blocker; CV, coefficient of variation; ESRD, end-stage renal disease; AER, albumin excretion rate; RAS, renin-angiotensin system; BBs, beta-blockers; CCBs, calcium channel blockers; FG, fasting glucose; DM, Diabetes Mellitus; HDL, high-density lipoprotein; DR, diabetes retinopathy; HVS, HbA1c variability score; MAP, mean arterial pressure; CRP, C-reactive protein; CVD, cardiovascular disease; MACE,major adverse cardiovascular events; UACR, urine albumin-to-creatinine ratio; HGI, hemoglobin glycation index; RAAS, renin-angiotensin-aldosterone system; DKD, diabetic kidney disease; IMD, inherited metabolic diseases; LDL, low-density lipoprotein; DPP-4i, dipeptidyl peptidase-4 inhibitors; GLP1-RA, glucagon-like peptide-1 agonists; TZD, thiazolidinedione; SGLT2i, sodium-glucose cotrans-porter-2 inhibitors.

Studies are presented in chronological order of publication.

**Table 2:** Characteristics of the studies considered in the meta-analysis(DR).

Study(Author, year)	Design(Type of study)	Number(male%)	Age at enrolment	Area (follow-up time, years)	Inclusion criteria	HbA1c variability and follow-up time	Mean HbA1c(%)	Outcome	Variable adjustment	NOS score
Hietala K et al.(2013) (58)	observational cohort study	1346(52.08%)	38.7±11.7	Finland(NA)	adults patients with T1DM	CV:0.084±0.044, number of HbA1c measurements: 10(3-18)	8.5±1.2	proliferative retinopathy	Adjusted for renal status, diabetes duration, mean HbA1c, blood pressure, sex and number of HbA1c measurements.	7
Penno G et al.(2013) (59)	prospective cohort study	8290(NA)	NA	Italia(NA)	caucasian patients with T2DM	SD:NA, NA	4.52±0.76	nonadvanced retinopathy	Adjusted for age, BMI, sex, known disease duration, smoking habits, TG, HDL-C, hypertension, dyslipidemia, previous major CVD events, specific treatments, and eGFR and albuminuria categories	7
Hermann JM et al.(2014) (60)	prospective cohort study	35891(52.3%)	16.2(13.1-18.0)	Germany(NA)	patients with T1DM	CV: 17.9(12.7–25.1), NA	4.3 (3.5–5.3)	development of DR	Adjusted for gender, age at diagnosis and median HbA1c	7
Virk SA et al.(2016) (66)	prospective cohort study	1706(47%)	NA	Canada(median follow-up period of 8.1 years)	patients with T1DM	SD:NA, CV:NA, number of HbA1c measurements: 22(14–29)	NA	retinopathy	Adjusted for age, sex, diabetes duration, SBP, DBP, cholesterol, height, BMI, and socioeconomic disadvantage.	6
Takao T et al.(2017) (67)	retrospective cohort study	486(83.3%)	55.4±9.3	Japan(NA)	patients with T2DM	CV:NA, NA	7.9±1.7	the development of mild-to-moderate NPDR	Adjusted for mean HbA1c, mean SBP, number of visits, age, sex, diabetes duration, BMI, TC/HDL-C, baseline smoking status, baseline alcohol intake, baseline use of insulin, and baseline use of ACEI.	6
Cardoso CRL et al.(2018) (68)	prospective cohort study	654(38.1%)	60.1(9.6)	Brazil(median follow-up period of 9.3 years (5.2–10.8))	adults patients with T2DM	SD:NA, NA	8.1(1.9)	retinopathy	Adjusted for age, sex and number of HbA1c or FG measurements, diabetes duration, BMI, smoking status, physical inactivity, arterial hypertension, number of anti-hypertensive drugs in use, ambulatory, 24-h SBP, presence of micro- and macrovascular complications at baseline, serum mean HDL-C and LDL-C, and use of insulin, statins and aspirin, mean fasting glycemia and HbA1c	8
Schreur V et al.(2018) (61)	observational cohort study	415(46.99%)	NA	Netherlands(7-65 years, median follow-up period of 29 years)	patients with T1DM	CV:NA, NA	NA	DR	NA	6
Rosa LCGFD et al.(2019) (69)	retrospective cohort study	220(40%)	29.6±10.1	Brazil(>10 years)	adults patients with T1DM	Adjust-SD:1.24±0.88, CV:1.38±0.63, NA	8.3±1.5	retinopathy	Adjusted for age, sex, T1DM duration, presence of hypertension, and mean LDL-C levels	6
Slieker RC et al.(2019) (70)	prospective cohort study	3898(NA)	NA	Netherlands(NA)	patients with T2DM	CV:NA, NA	NA	retinopathy	Adjusted for sex, BMI, HDL, age at diagnosis, TG, HbA1c at baseline, oral glucose lowering drugs, insulin use and eGFR.	6
Song KH et al.(2019) (71)	retrospective cohort study.	604(54.5%)	60.7±10.8	Korea(3 years)	patients with T2DM	SD:NA, 3-6 months	7.32±1.04	the progression of DR (worsening of the stage of DR)	Adjusted for eGFR, TG to HDL-C ratio, the presence of DR, and use of ACEI or ARB.	7
Romero-Aroca P et al.(2021) (73)	prospective cohort study	366(NA)	NA	Spain(12 years)	patients with T1DM	SD:NA, CV:NA, NA	NA	DR/ DR severity	Adjusted for current age, arterial hypertension, eGFR and mean-HbA1c.	6
Dai D et al.(2021) (62)	prospective cohort study	315(60.6%)	58.0±10.1	China(NA)	patients with T2DM	CV:6.92±5.12, NA	7.67±1.32	DR	Adjusted for diabetes duration, smoking status, SBP, UACR, TG, fibrates using, and mean glycated albumin.	7
Hu J et al.(2021) (63)	observational cohort study	3152(52.0%)	NA	Taiwan, China(median follow-up period of 3.95 years(2-5))	patients with T2DM	SD:NA, NA	DR:9.1 ± 2.1 No DR:8.5 ± 2.0	DR	Adjused for age, sex, diabetes duration, cataract prevalence, mean-HbA1c, HbA1c-SD, BMI, WHR, SBP, DBP, TC, TG, LDL, HDL	7
Kim HU et al.(2021) (64)	retrospective cohort study	434(54.84%)	NA	Korea(NA)	patients with T2DM	CV: No DR development: 9.5±4.6, DR development: 11.4±5.9, NA	No DR development:7.3±0.8 DR development:8.±1.0	any DR development/ moderate NPDR or worse DR	Adjused for age, diabetes duration, insulin, SGLT-2 inhibitor, hemoglobin, TG, mean HbA1c, HbA1c ARV	6
Lee S et al.(2021) (72)	retrospective cohort study	3137(NA)	NA	Hong Kong, China(10 years)	patients with T2DM	SD:1.1±0.71, CV:13.6±7.6, the average number of HbA1c measurements: 11.9±4.8	8.1±1.8	Ophthalmological complications	NA	6
Wakasugi S et al.(2021) (74)	prospective cohort study	999(60.9%)	64.6±9.6	Japan(NA)	patients with T2DM, age≥30 years and≤80 years	SD:2.04±0.63, NA	7.1±0.8	DR severity	Adjusted for age, gender, BMI, and duration of diabetes, SBP, TC, HDL-C, logarithm of TG, serum uric acid, eGFR, logarithm of urinary albumin excretion, smoker, alcohol consumption, use of insulin therapy, use of ACEI and/or ARB, use of statins, and use of antiplatelet agents and HbA1c	8
Ma C et al.(2022) (75)	observational cohort study	2161(38.45%)	NA	China(NA)	patients with T2DM	Adjust-SD:NA, CV:NA, NA	NA	diabetic eye disease events	Adjusted for gender, age, duration of T2DM, BMI, smoking, baseline concomitant disease, TG, LDL-C, blood pressure, anti-hyperglycemic therapy, and ACEI or ARB treatment, average HbA1c	7
Wu TE et al.(2022) (12)	prospective cohort study	1869(50.4%)	63.2±12.7	Taiwan, China(median follow-up period of 9.5 years)	patients with T2DM	SD:0.728 ± 0.528, the average number of HbA1c measurements: 19, 10 to 42	8.06±1.77	any retinopathy/advanced retinopathy	Adjusted for HbA1c-mean , age, sex, diabetes duration, blood pressure, BMI, TC, HDL-C, TG, and smoking status	7
Sun B et al.(2022) (76)	retrospective cohort study	855(NA)	NA	China(median follow-up period of 4.8 years)	patients with T2DM	CV:NA, at 3, 6, 12, 18, 24 months, and every 6 months thereafter	NA	New or worsening retinopathy	Adjusted for age, duration of diabetes, gender, BMI, current smoking status, SBP and DBP, TC, TG, HDL-C and LDL-C, baseline use of insulin and mean HbA1c during the first 24 months, history of major macrovascular diseases and microvascular diseases	7
Ma Y et al.(2023) (77)	retrospective cohort study	387(61.5%)	48.85±9.42	China(median follow-up period of 4.5 years)	patients with T2DM	CV:9.17(6.54,12.55), NA	7.31±1.57	retinopathy	NA	6
Suh J et al.(2023) (78)	retrospective cohort study	201(43.8%)	NA	Korea(median follow-up period of 16.4 years)	children and adolescents with T1DM	Adjust-SD:1.04±0.58, 3 months	NA	retinopathy	Adjusted for age, duration of disease, and sex	7
Dehghani Firouzabadi F et al.(2024) (66)	prospective cohort study	1145(50.04%)	NA	Iran(10 years)	patients with T2DM	CV:NA, 3 months	Developed retinopathy: 7.85±0.82; Did not develop Retinopathy: 7.54±0.83	incidence of retinopathy	NA	6
Teh XR et al.(2025) (79)	retrospective cohort study	40662(38.3%)	57.2(13.9)	Thailand(10 years)	patients with T2DM	SD:0.67(0.87), CV:0.07(0.08), 3-6 months	7.7(2.0)	DR	Adjusted for age, gender, insurance scheme, BMI, TC, LDL, HDL, TG, haemoglobin, SBP/DBP, hypertension, dyslipidemia, presence of T2DM complications (CVD, DR, or CKD) prior to the outcome of interest, medication use in terms of drug classes (biguanides, sulphonylurea, insulin, alpha-glucosidase inhibitors, DPP-4i, GLP1-RA, TZD, SGLT-2 inhibitors , meglitinides, statins) and the number of antihypertensive drugs	8

DR, diabetes retinopathy; HbA1c, glycated hemoglobin A1C; NOS, Newcastle-Ottawa Scale; T1DM, Type 1 Diabetes Mellitus; NA, not available; CV, coefficient of variation; T2DM, Type 2 Diabetes Mellitus; SD, standard deviation; BMI, mean body mass index; HDL-C, high-density lipoprotein cholesterol; CVD, cardiovascular disease; eGFR, estimated glomerular filtration rate; SBP, systolic blood pressure; DBP, diastolic blood pressure; NPDR, non-proliferative diabetic retinopathy; TC, total cholesterol; ACEI, angiotensin-converting enzyme inhibitor; FG, fasting glucose; LDL-C, low-density lipoprotein cholesterol; HDL, high-density lipoprotein; ARB, angiotensin-converting enzyme receptor blocker; UACR, urine albumin-to-creatinine ratio; TG, triglyceride; NDR, non-diabetic renal disease; WHR, waist-to-hip ratio; LDL, low-density lipoprotein; SGLT-2, sodium-glucose cotransporter-2; ARV; DM, diabetes mellitus; CKD, chronic kidney disease; DPP-4i, dipeptidyl peptidase-4 inhibitors; GLP1-RA, glucagon-like peptide-1 agonists; TZD, thiazolidinedione.

Studies are presented in chronological order of publication.

### 3.2 Study on the association between HbA1c variability and diabetic kidney disease

#### 3.2.1 HbA1c variability and type 1 diabetic kidney disease

##### 3.2.1.1 HbA1c-CV and incidence of kidney disease

**Figure 2:**
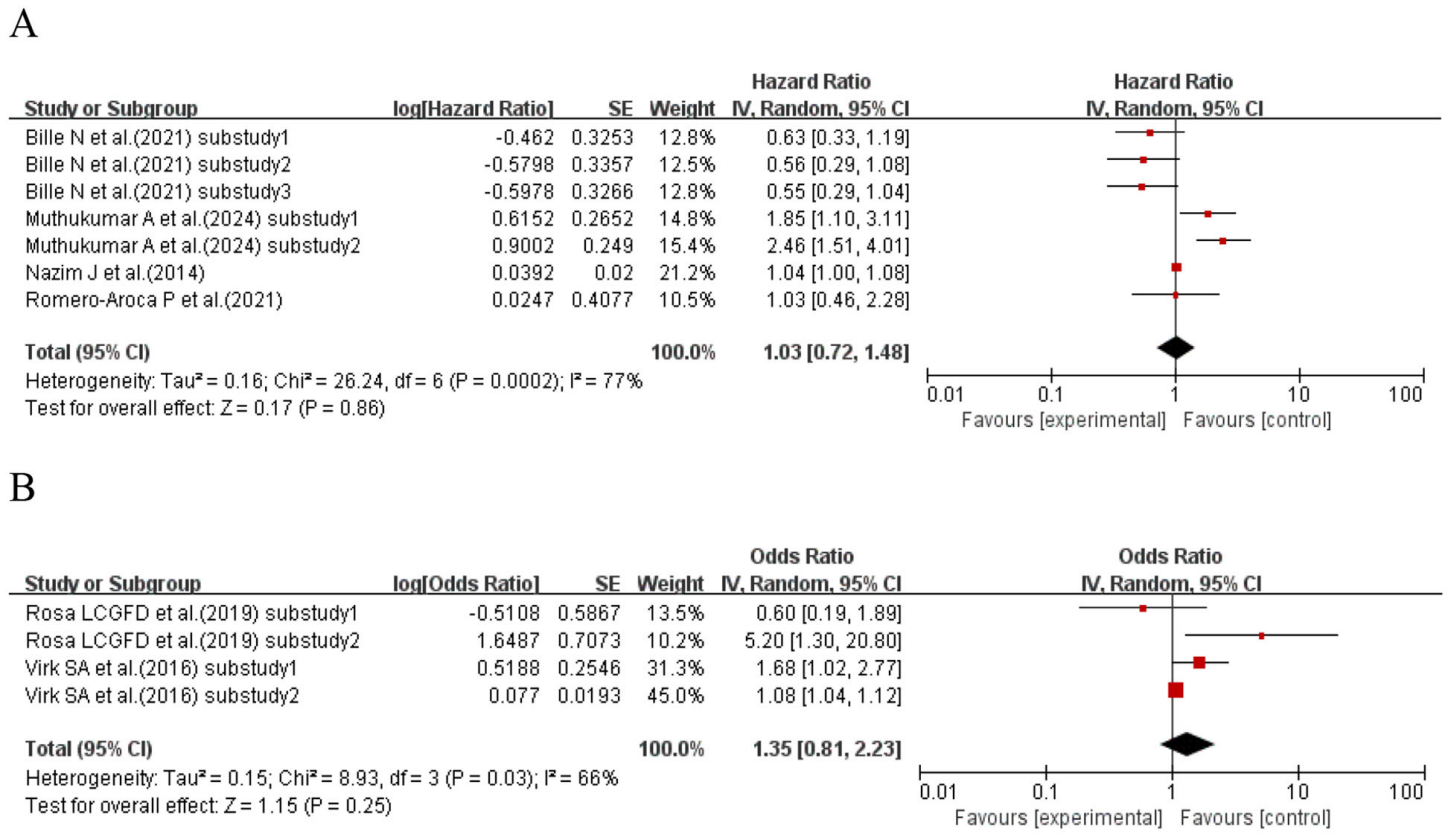
Forest plot showing the incidence of diabetic kidney disease, including **(A)** Hazard Ratio (HR) and **(B)** Odds Ratio (OR) for HbA1c variability measured by HbA1c-CV in patients with T1DM based on published reports.

When the effect size was HR, four studies (42, 50, 57, 73) (seven sub-studies total) explored the association between CV and kidney disease in T1DM patients. Heterogeneity existed among studies (I^2^ = 77%, p = 0.0002), so a random-effects model was used. Meta-analysis results did not support an association between CV and kidney disease risk in T1DM patients (HR = 1.03, 95% CI: 0.72–1.48, p = 0.86 > 0.05). When the effect size was OR, two studies (66, 69) (four sub-studies total) explored the association between CV and kidney disease in T1DM patients. Heterogeneity existed among studies (I^2^ = 66%, p = 0.03), so a random-effects model was used. Meta-analysis results showed no significant association between CV and kidney disease risk in T1DM patients (OR = 1.35, 95% CI: 0.81–2.23, p = 0.25 > 0.05). See **Figure 2** for details.

##### 3.2.1.2 HbA1c-SD and incidence of kidney disease

**Figure 3:**
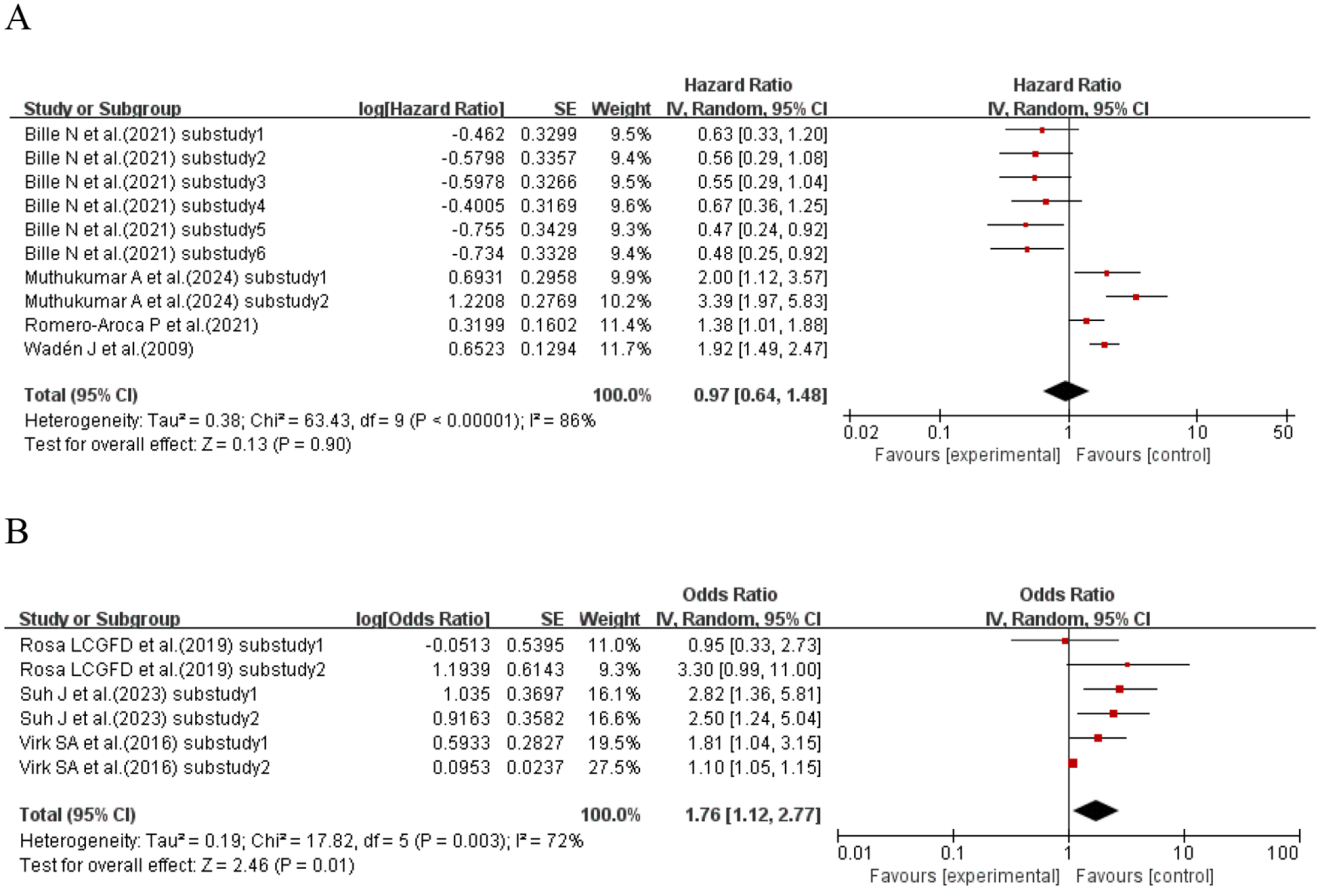
Forest plot showing the incidence of diabetic kidney disease, including **(A)** Hazard Ratio (HR) and **(B)** Odds Ratio (OR) for HbA1c variability measured by HbA1c-SD in patients with T1DM based on published reports.

When the effect size was HR, a total of four studies (37, 50, 57, 73) (comprising 10 sub-studies) explored the association between SD and kidney disease in patients with T1DM. Heterogeneity was observed among the studies (I^2^ = 86%, p< 0.00001), so a random-effects model was used for analysis. The meta-analysis results failed to support an association between SD and kidney disease risk in T1DM patients (HR = 0.97, 95% CI: 0.64–1.48, p = 0.90 > 0.05). When the effect size was OR, a total of three studies (66, 69, 78) (comprising six sub-studies) explored the association between SD and kidney disease in T1DM patients. Heterogeneity existed among the studies (I^2^ = 72%, p = 0.003), and a random-effects model was adopted for analysis. The meta-analysis results indicated that SD was a risk factor for kidney disease in T1DM patients (OR = 1.76, 95% CI: 1.12–2.77, p = 0.01). See **Figure 3** for details.

##### 3.2.1.3 HbA1c-HVS and incidence of kidney disease

**Figure 4:**
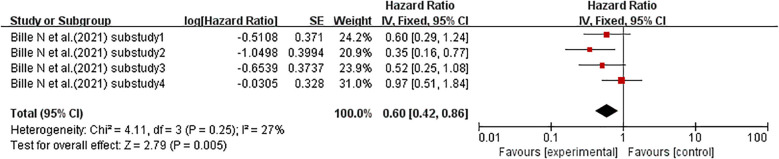
Forest plot of data on the incidence of diabetic kidney disease. HR for HbA1c-HVS based on published reports of T1DM. HR, hazard ratio; OR, odds ratio; HbA1c, glycated hemoglobin; HVS, HbA1c variability score; T1DM, type 1 diabetes mellitus.

When the effect size was HR, one study (50) (with four sub-studies total) explored the association between HVS and kidney disease in T1DM patients. There was no significant heterogeneity among the sub-studies (I^2^ = 27%, p = 0.25), so a fixed-effects model was used for analysis. The meta-analysis results showed no significant association between HVS and kidney disease risk in T1DM patients (HR = 0.60, 95% CI: 0.42–0.86, p = 0.005). See **Figure 4** for details.

##### 3.2.1.3 HbA1c-per 1% increase in SD and incidence of kidney disease

**Figure 5:**
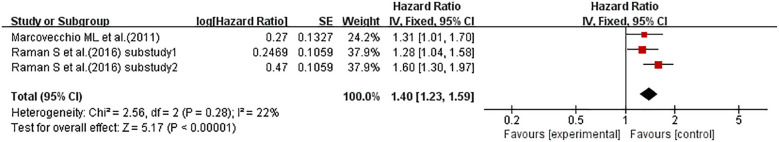
Forest plot of data on the incidence of diabetic kidney disease. HR for HbA1c (per 1% increase in SD) based on published reports of T1DM. HR, hazard ratio; HbA1c, glycated hemoglobin; T1DM, type 1 diabetes mellitus.

When the effect size was HR, two studies (38, 45) (with a total of three sub-studies) explored the association between HVS and kidney disease in T1DM patients. The study heterogeneity was low (I^2^ = 22%, p = 0.28), so a fixed-effects model was used for analysis. The meta-analysis results showed that a per 1% increase in SD was significantly associated with the risk of kidney disease in T1DM patients (HR = 1.40, 95% CI: 1.23–1.59, p< 0.00001). See **Figure 5** for details.

#### 3.2.2 HbA1c Variability and type 2 diabetic kidney disease

##### 3.2.2.1 HbA1c-CV and incidence of kidney disease

**Figure 6:**
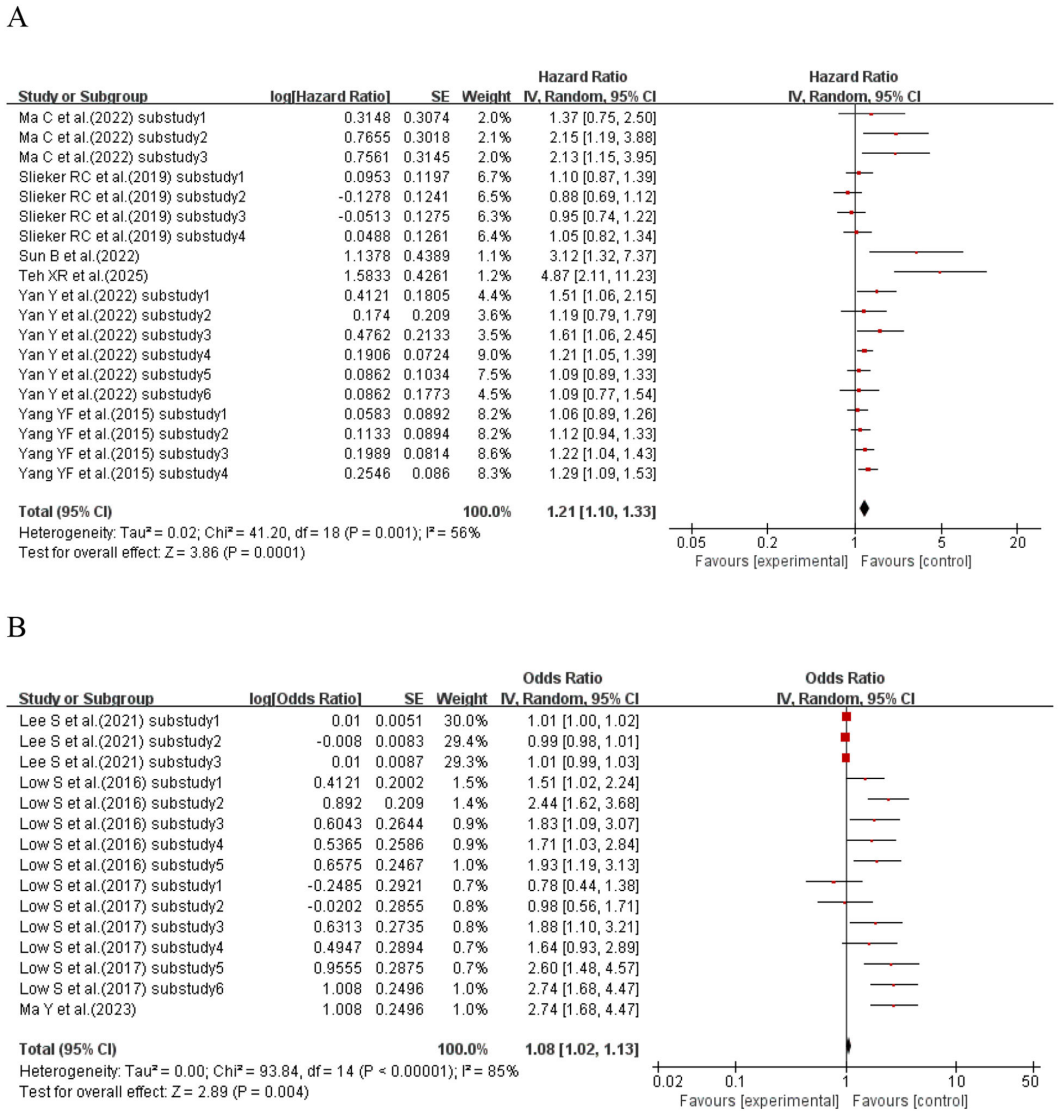
Forest plot showing the incidence of diabetic kidney disease, including **(A)** Hazard Ratio (HR) and **(B)** Odds Ratio (OR) for HbA1c variability measured by HbA1c-CV in patients with T2DM based on published reports.

When the effect size was HR, six studies (43, 53, 70, 75, 76, 79) (19 sub-studies) explored CV and kidney disease in T2DM patients. Heterogeneity existed (I^2^ = 56%, p = 0.001); a random-effects model was used. Meta-analysis showed a significant association between CV and kidney disease risk in T2DM patients (HR = 1.21, 95% CI: 1.10–1.33, p = 0.0001). When the effect size was OR, four studies (44, 46, 72, 77) (15 sub-studies) explored CV and kidney disease in T2DM patients. Heterogeneity existed (I^2^ = 85%, p< 0.00001); a random-effects model was used. Meta-analysis showed a weak association between higher CV and kidney disease risk in T2DM patients (OR = 1.08, 95% CI: 1.02–1.13, p = 0.004). See **Figure 6** for details.

##### 3.2.2.2 HbA1c-SD and incidence of kidney disease

**Figure 7:**
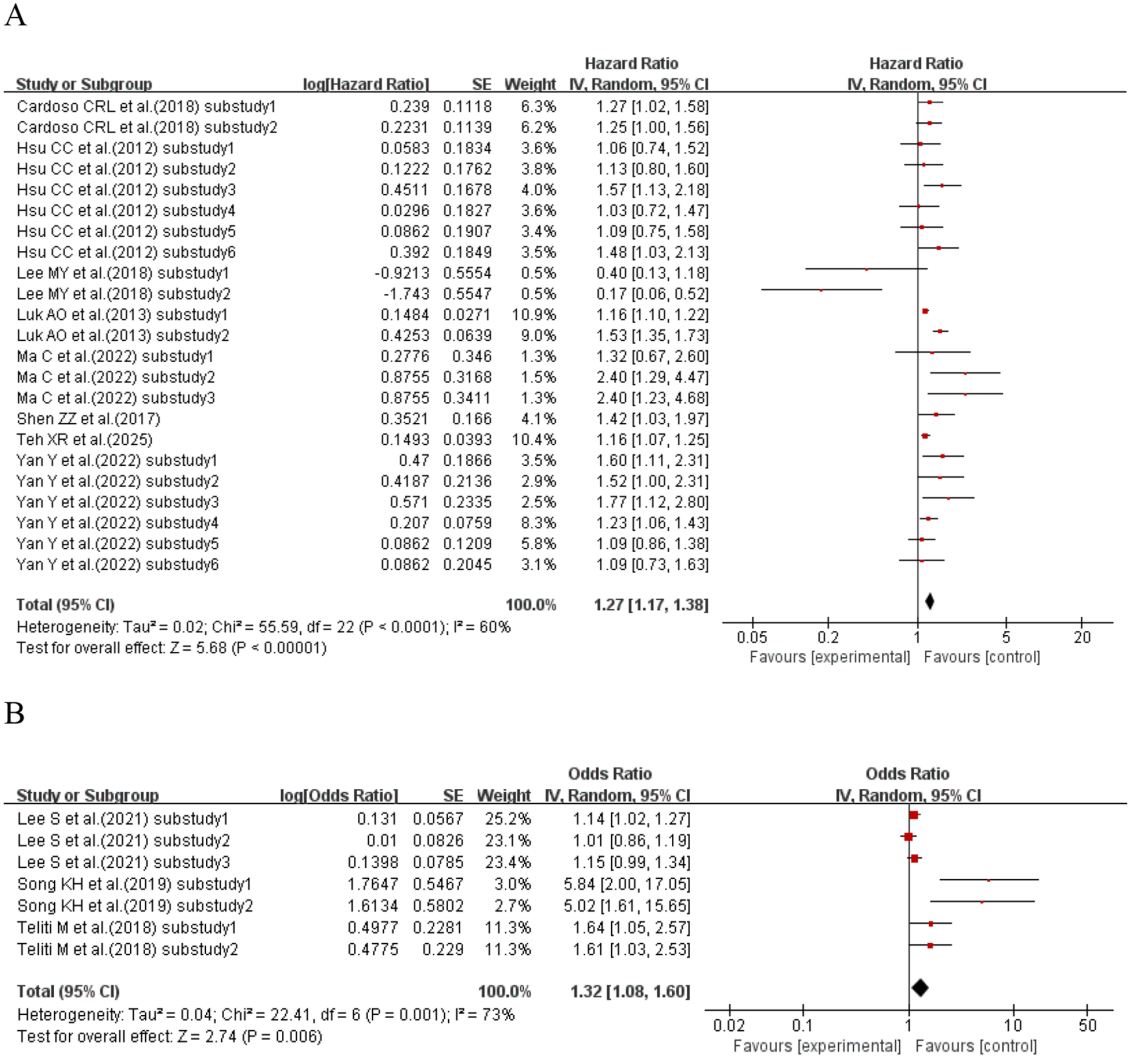
Forest plot showing the incidence of diabetic kidney disease, including **(A)** Hazard Ratio (HR) and **(B)** Odds Ratio (OR) for HbA1c variability measured by HbA1c-SD in patients with T2DM based on published reports.

When the effect size was HR, eight studies (18, 39, 47, 48, 53, 68, 75, 79) (23 sub-studies) explored SD and kidney disease in T2DM patients. Heterogeneity existed (I^2^ = 60%, p< 0.0001); a random-effects model was used. Meta-analysis showed that T2DM patients with higher SD had 27% increased kidney disease risk vs. those with lower SD (HR = 1.27, 95% CI: 1.17–1.38, p< 0.00001). When the effect size was OR, three studies (49, 71, 72) (seven sub-studies) explored SD and kidney disease in T2DM patients. Heterogeneity existed (I^2^ = 73%, p = 0.001); a random-effects model was used. Meta-analysis showed that higher SD was a risk factor for kidney disease in T2DM patients (OR = 1.32, 95% CI: 1.08–1.60, p = 0.006). See **Figure 7** for details.

##### 3.2.2.3 HbA1c-HGI and incidence of kidney disease

**Figure 8:**
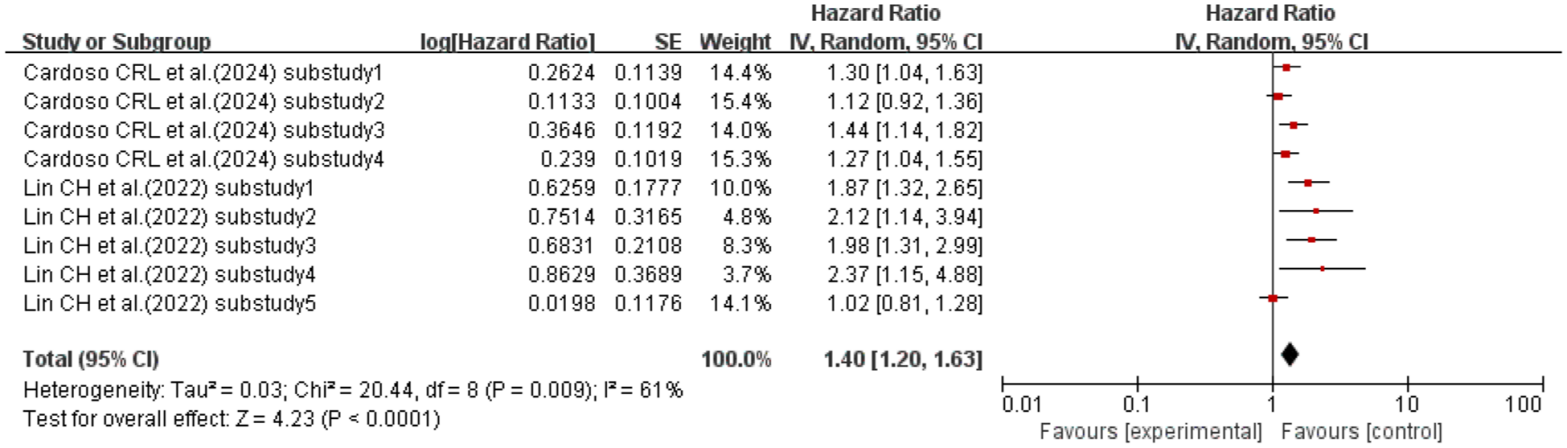
Forest plot of diabetic kidney disease incidence data. HR for HbA1c-HGI based on published reports of T2DM. HR, hazard ratio; HbA1c, glycated hemoglobin; HGI, hemoglobin glycation index; T2DM, type 2 diabetes mellitus.

When the effect size was HR, two studies (52, 56) (with a total of nine sub-studies) explored the association between HGI and kidney disease in T2DM patients. Heterogeneity existed among the studies (I^2^ = 61%, p = 0.009), so a random-effects model was used for analysis. The meta-analysis results showed that compared with T2DM patients with lower HGI, those with higher HGI had a 40% increased risk of kidney disease (HR = 1.40, 95% CI: 1.20–1.63, p< 0.0001). See **Figure 8** for details.

##### 3.2.2.4 HbA1c-HVS and incidence of kidney disease

**Figure 9:**
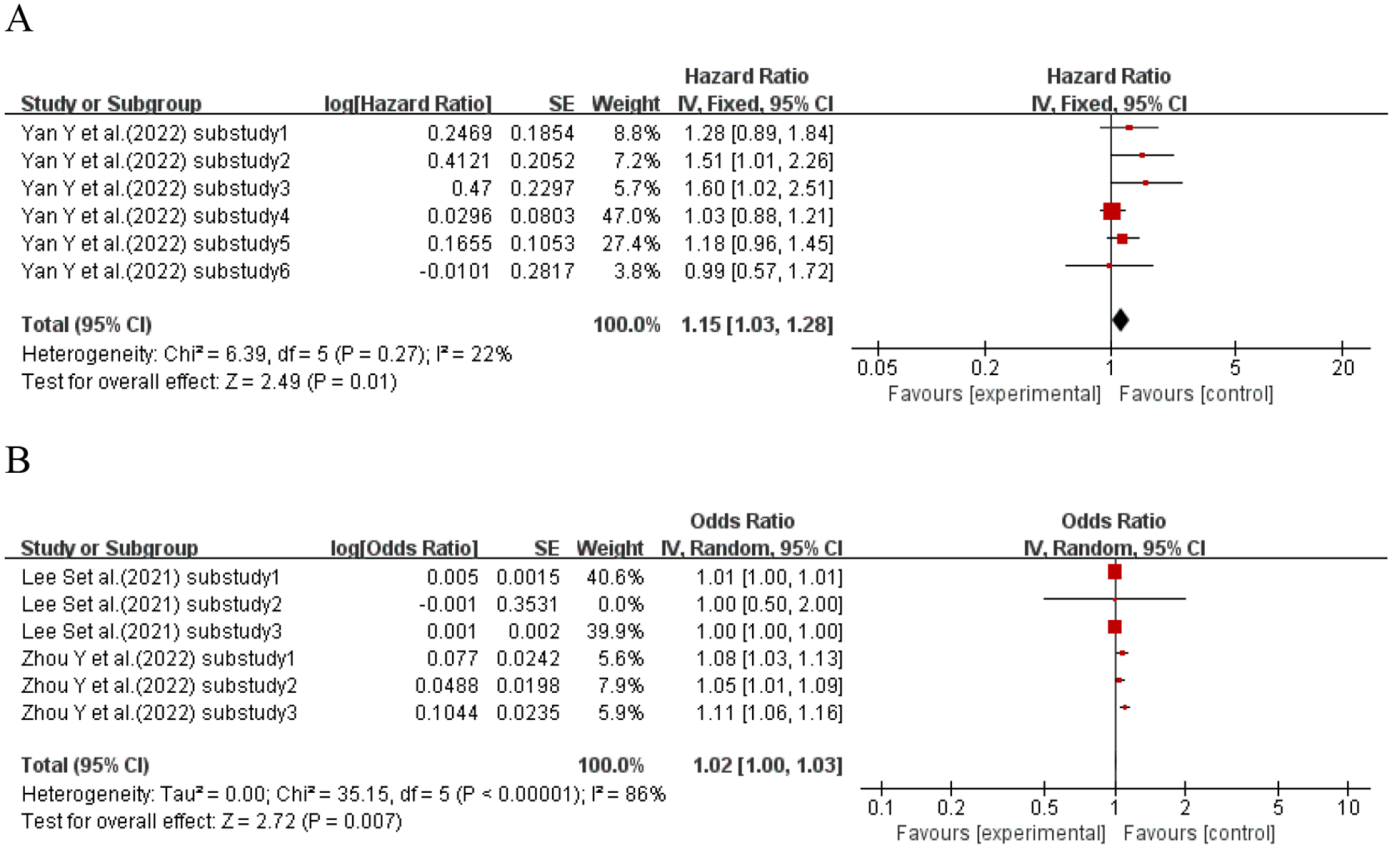
Forest plot showing the incidence of diabetic kidney disease, including **(A)** Hazard Ratio (HR) and **(B)** Odds Ratio (OR) for HbA1c variability measured by HbA1c-HVS in patients with T2DM based on published reports.

When the effect size was HR, one study (53) (six sub-studies) explored HVS and kidney disease in T2DM patients. No significant heterogeneity existed (I^2^ = 22%, p = 0.27); a fixed-effects model was used. Meta-analysis showed that T2DM patients with higher HVS had 15% increased kidney disease risk vs. those with lower HVS (HR = 1.15, 95% CI: 1.03–1.28, p = 0.01). When the effect size was OR, two studies (54, 72) (six sub-studies) explored HVS and kidney disease in T2DM patients. Meta-analysis showed no significant association between HVS and kidney disease risk in T2DM patients (OR = 1.02, 95% CI: 1.00–1.03, p = 0.007). See **Figure 9** for details.

##### 3.2.2.5 HbA1c-per 1% increase in CV and incidence of kidney disease

**Figure 10:**
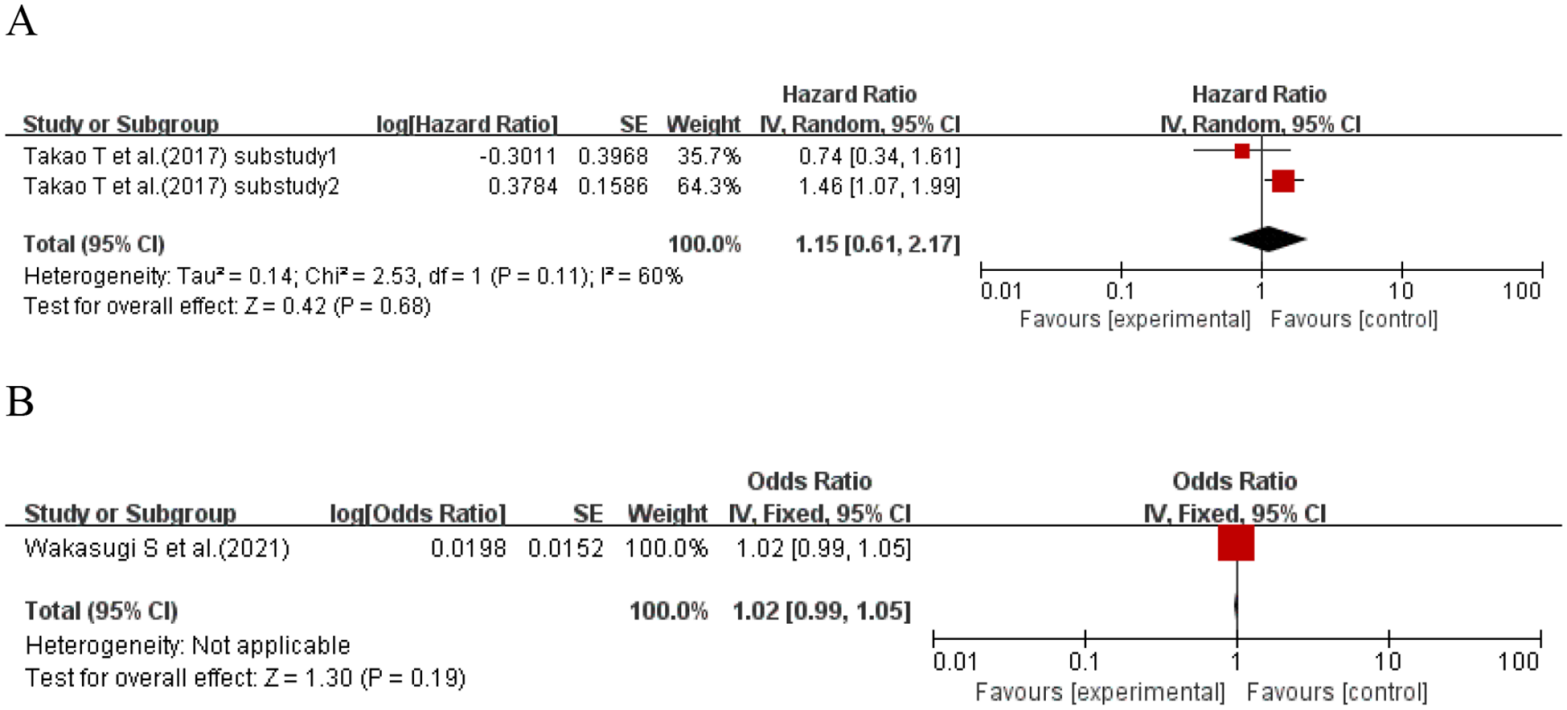
Forest plot showing the incidence of diabetic kidney disease, including **(A)** Hazard Ratio (HR) and **(B)** Odds Ratio (OR) for HbA1c-per 1% increase in CV in patients with T2DM based on published reports.

One study (67) (two sub-studies) explored the per 1% increase in CV and kidney disease in T2DM patients. Meta-analysis showed no significant association between a per 1% increase in CV and kidney disease risk in T2DM patients (HR = 1.15, 95% CI: 0.61–2.17, p = 0.68 > 0.05). One study (74) explored the per 1% increase in CV and kidney disease in T2DM patients. Meta-analysis failed to support an association between a per 1% increase in CV and kidney disease risk in T2DM patients (OR = 1.02, 95% CI: 0.99–1.05, p = 0.19 > 0.05). See **Figure 10** for details.

##### 3.2.2.6 HbA1c-per 1% increase in SD and incidence of kidney disease

**Figure 11:**
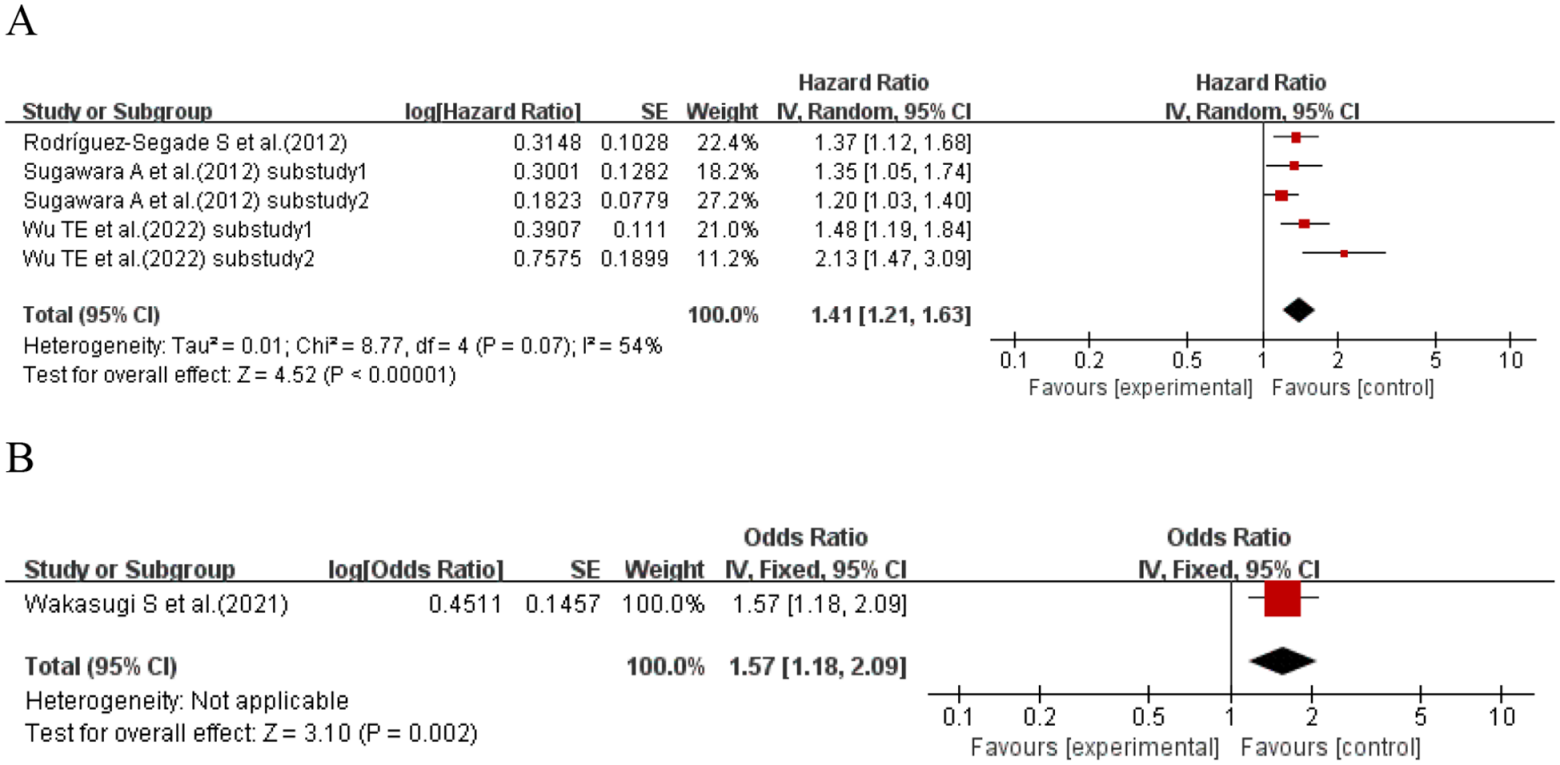
Forest plot showing the incidence of diabetic kidney disease, including **(A)** Hazard Ratio (HR) and **(B)** Odds Ratio (OR) for HbA1c-per 1% increase in SD in patients with T2DM based on published reports.

Three studies (12, 40, 41) (five sub-studies) explored the per 1% increase in SD and kidney disease in T2DM patients. Heterogeneity existed (I^2^ = 54%, p = 0.07); a random-effects model was used. Meta-analysis showed a significant association between a per 1% increase in SD and kidney disease progression risk in T2DM patients (HR = 1.41, 95% CI: 1.21–1.63, p< 0.00001). One study (74) explored the per 1% increase in SD and kidney disease in T2DM patients. Meta-analysis showed that a per 1% increase in SD was a risk factor for kidney disease in T2DM patients (OR = 1.57, 95% CI: 1.18–2.09, p = 0.002). See **Figure 11** for details.

##### 3.2.2.7 HbA1c-CV and mortality of kidney disease

**Figure 12:**
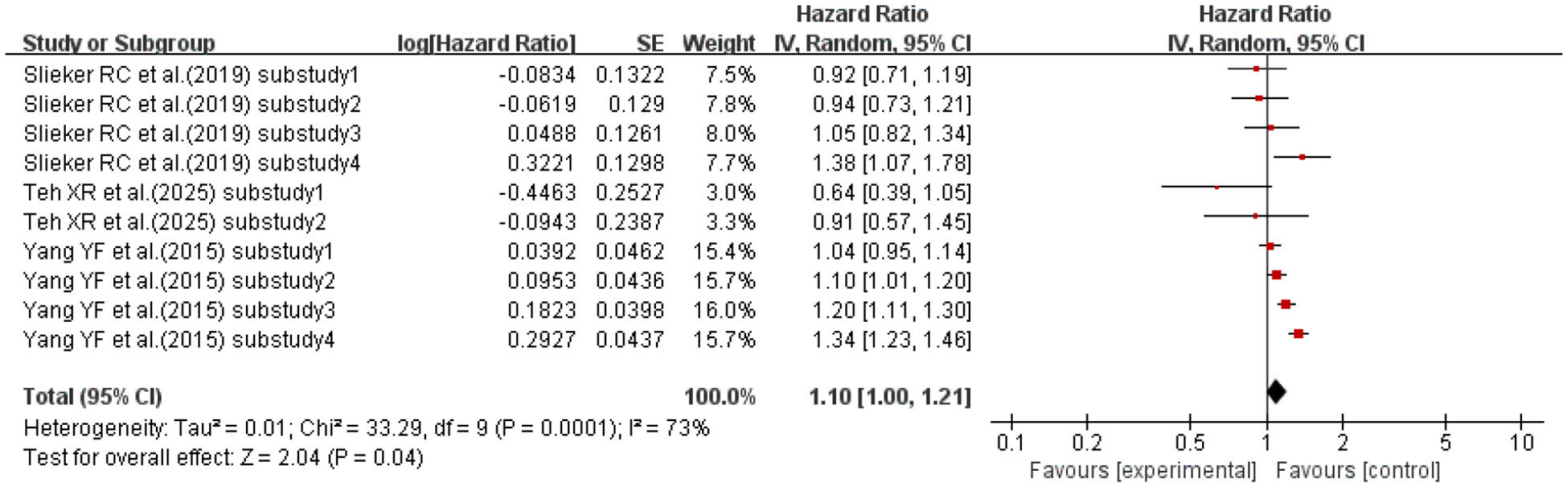
Forest plot of mortality data in diabetic kidney disease. HR for HbA1c-CV based on published reports of T2DM. HR, hazard ratio; HbA1c, glycated hemoglobin; CV, coefficient of variation; T2DM, type 2 diabetes mellitus.

When the effect size was HR, three studies (43, 70, 79) (with a total of 10 sub-studies) explored the association between CV and mortality in T2DM patients. The meta-analysis results showed that compared with T2DM patients with lower CV, those with higher CV had a 10% increased risk of mortality (HR = 1.10, 95% CI: 1.00–1.21, p = 0.04). See **Figure 12** for details.

##### 3.2.2.8 HbA1c-SD and mortality of kidney disease

**Figure 13:**
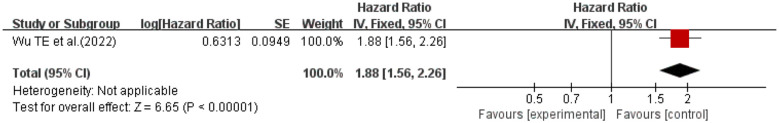
Forest plot of mortality data in diabetic kidney disease. HR for HbA1c-SD based on published reports of T2DM. HR, hazard ratio; HbA1c, glycated hemoglobin; SD, standard deviation; T2DM, type 2 diabetes mellitus.

When the effect size was HR, one study (12) explored the association between SD and mortality in T2DM patients, using a fixed-effects model for analysis. The meta-analysis results showed that compared with T2DM patients with lower SD, those with higher SD had an 88% increased risk of mortality (HR = 1.88, 95% CI: 1.56–2.26, p< 0.00001). See **Figure 13** for details.

##### 3.2.2.9 HbA1c-HVS and mortality of kidney disease

**Figure 14:**
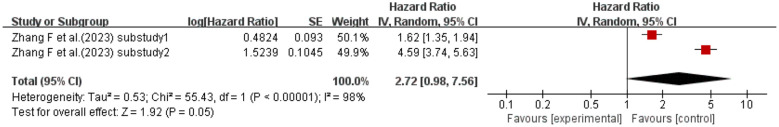
Forest plot of mortality data in diabetic kidney disease. HR for HbA1c-HVS based on published reports of T2DM. HR, hazard ratio; HbA1c, glycated hemoglobin; HVS, HbA1c variability score; T2DM, type 2 diabetes mellitus.

When the effect size was HR, one study (55) (with a total of two sub-studies) explored the association between HVS and mortality in T2DM patients. The meta-analysis results showed that compared with T2DM patients with lower HVS, those with higher HVS had a 172% increased risk of mortality (HR = 2.72, 95% CI: 0.98–7.56, p< 0.00001). See **Figure 14** for details.

#### 3.2.3 HbA1c variability and type 1 and type 2 diabetic kidney disease

**Figure 15:**
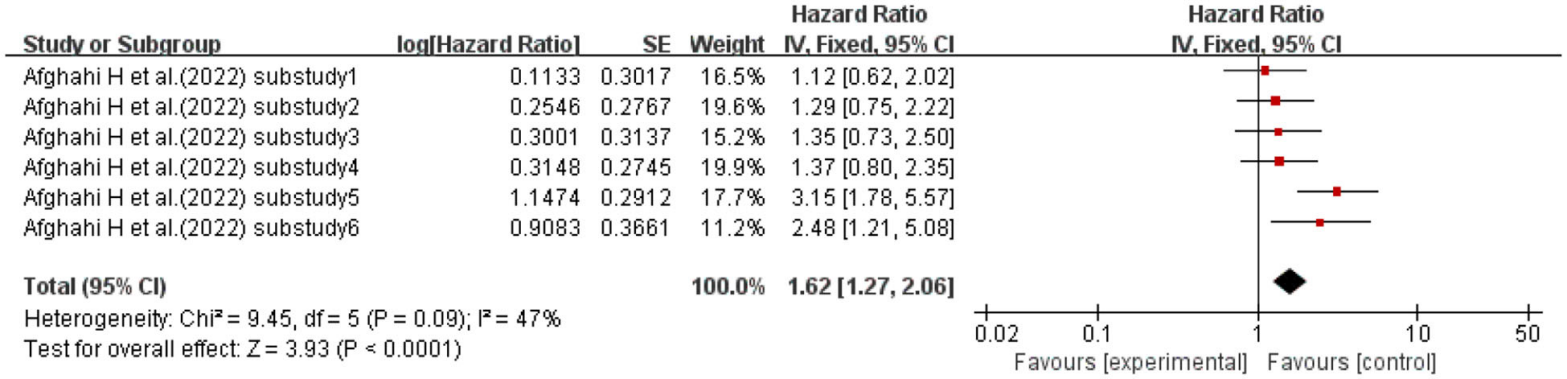
Forest plot of diabetic kidney disease mortality data. HR for HbA1c-CV based on published reports of DM. HR, hazard ratio; HbA1c, glycated hemoglobin; CV, coefficient of variation; DM, diabetes mellitus.

When the effect size was HR, one study (51) (six sub-studies, diabetic participants) explored the association between CV and mortality in diabetic patients. No significant heterogeneity existed (I^2^ = 47%, p = 0.09); a fixed-effects model was used. Meta-analysis showed that diabetic patients with higher CV had a 62% increased mortality risk vs. those with lower CV (HR = 1.62, 95% CI: 1.27–2.06, p< 0.0001). See **Figure 15** for details.

### 3.3 Study on the association between HbA1c variability and DR outcomes

#### 3.3.1 HbA1c variability and type 1 DR outcomes

##### 3.3.1.1 HbA1c-CV and incidence of DR

**Figure 16:**
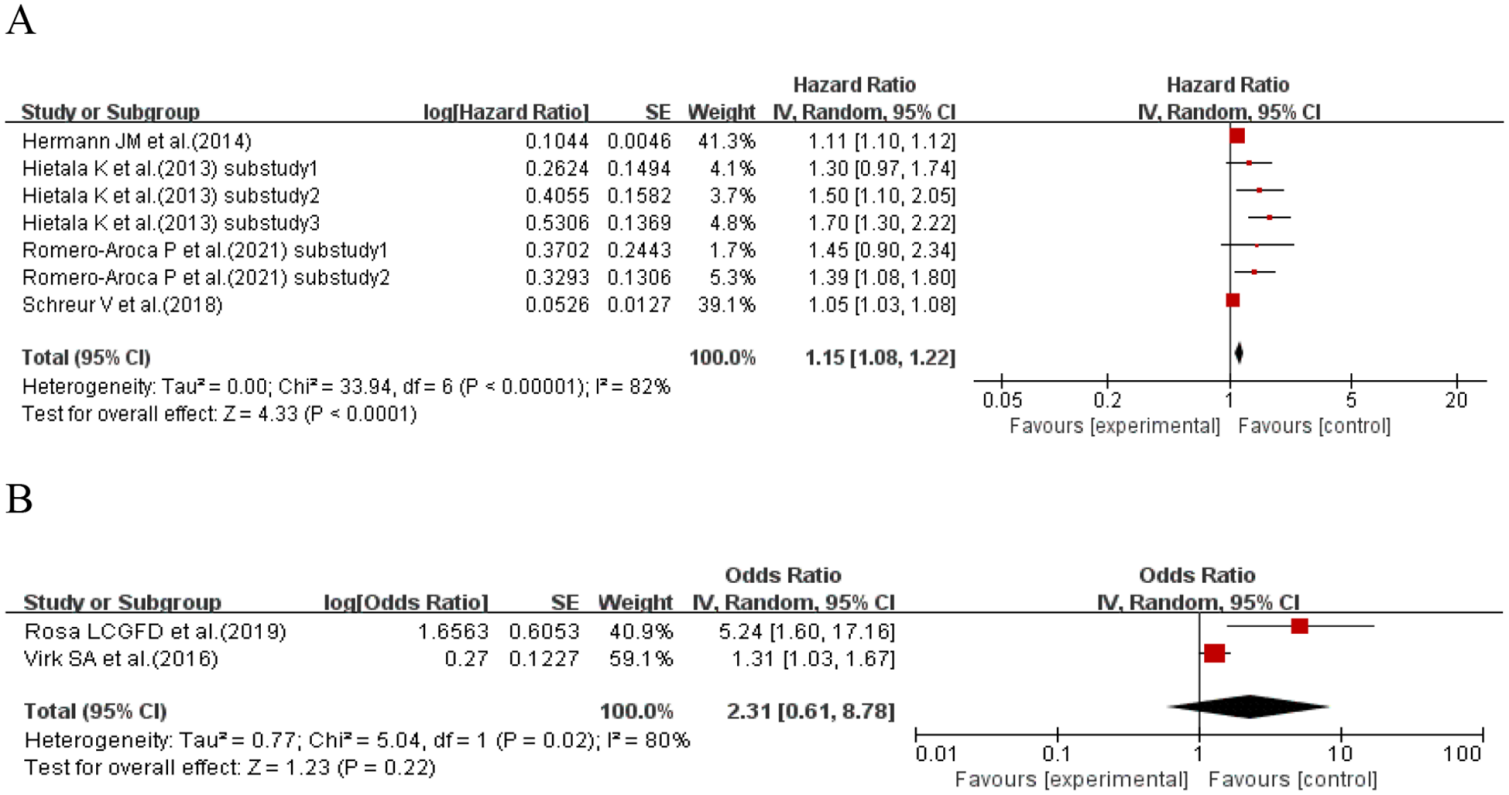
Forest plot showing the incidence of diabetic retinopathy, including **(A)** Hazard Ratio (HR) and **(B)** Odds Ratio (OR) for HbA1c variability measured by HbA1c-CV in patients with T1DM based on published reports.

When the effect size was HR, four studies (58, 60, 61, 73) (seven sub-studies) explored CV and retinopathy in T1DM patients. Meta-analysis showed that T1DM patients with higher CV had a 15% increased retinopathy risk vs. those with lower CV (HR = 1.15, 95% CI: 1.08–1.22, p< 0.0001). When the effect size was OR, two studies (66, 69) explored CV and retinopathy in T1DM patients. Meta-analysis showed no significant association between CV and retinopathy risk in T1DM patients (OR = 2.31, 95% CI: 0.61–8.78, p = 0.22 > 0.05). See **Figure 16** for details.

##### 3.3.1.2 HbA1c-SD and incidence of DR

**Figure 17:**
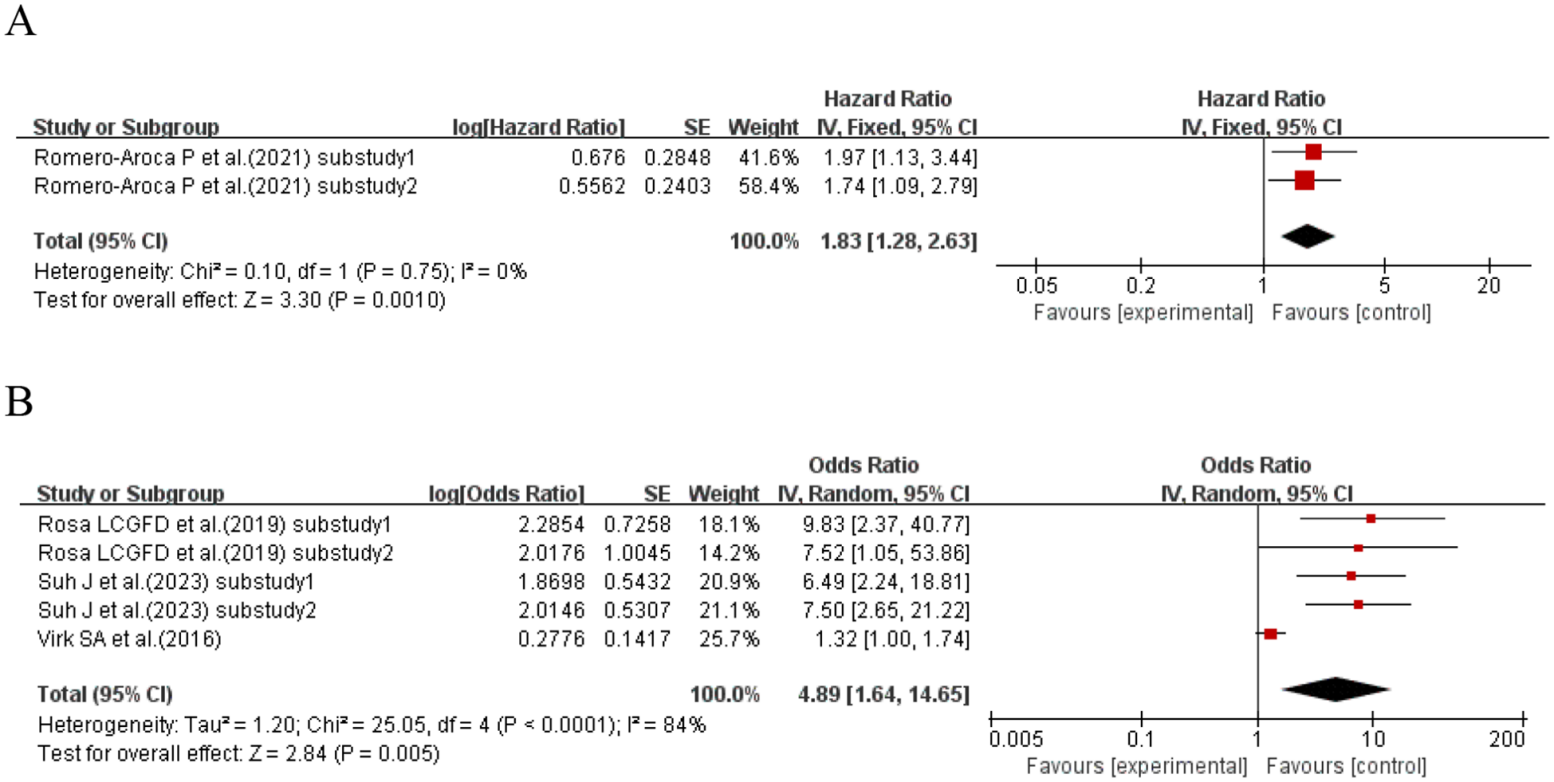
Forest plot showing the incidence of diabetic retinopathy, including **(A)** Hazard Ratio (HR) and **(B)** Odds Ratio (OR) for HbA1c variability measured by HbA1c-SD in patients with T1DM based on published reports.

When the effect size was HR, one study (73) (with a total of two sub-studies) explored the association between SD and retinopathy in T1DM patients. The meta-analysis results showed that compared with T1DM patients with lower SD, those with higher SD had an 83% increased risk of developing retinopathy (HR = 1.83, 95% CI: 1.28–2.63, p = 0.001). When the effect size was OR, three studies (66, 69, 78) (with a total of five sub-studies) explored the association between SD and retinopathy in T1DM patients. The meta-analysis results showed that higher SD was a risk factor for retinopathy in T1DM patients (OR = 4.89, 95% CI: 1.64–14.65, p = 0.005). See **Figure 17** for details.

#### 3.3.2 HbA1c variability and type 2 DR outcomes

##### 3.3.2.1 HbA1c-CV and incidence of DR

**Figure 18:**
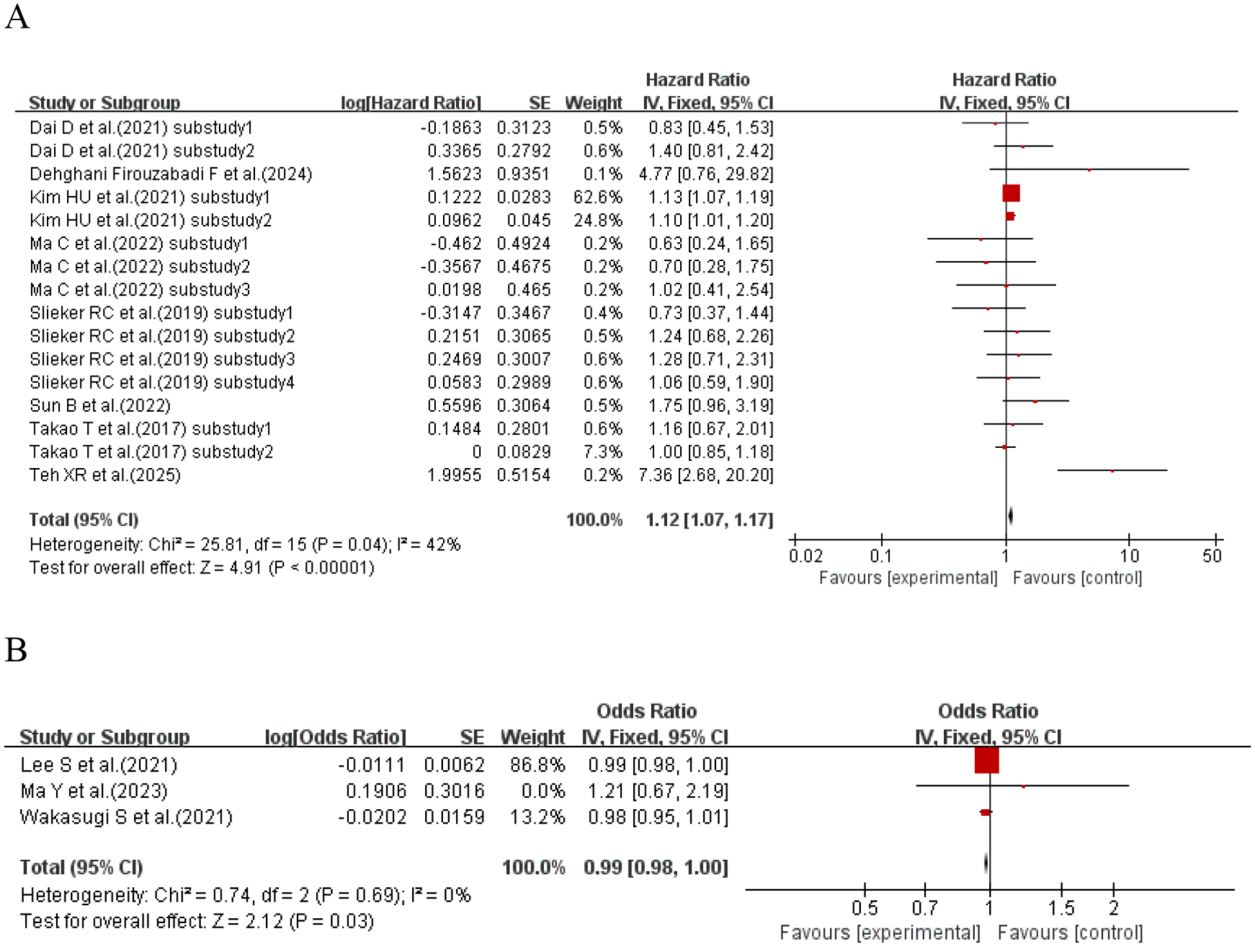
Forest plot showing the incidence of diabetic retinopathy, including **(A)** Hazard Ratio (HR) and **(B)** Odds Ratio (OR) for HbA1c variability measured by HbA1c-CV in patients with T2DM based on published reports.

When the effect size was HR, eight studies (62, 64, 65, 67, 70, 75, 76, 79) (with a total of 16 sub-studies) explored the association between CV and retinopathy in T2DM patients. The meta-analysis results showed that compared with T2DM patients with lower CV, those with higher CV had a 12% increased risk of developing retinopathy (HR = 1.12, 95% CI: 1.07–1.17, p< 0.00001). When the effect size was OR, three studies (72, 74, 77) explored the association between CV and retinopathy in T2DM patients. The meta-analysis results showed no significant association between CV and retinopathy risk in T2DM patients (OR = 0.99, 95% CI: 0.98–1.00, p = 0.03). See **Figure 18** for details.

##### 3.3.2.2 HbA1c-SD and incidence of DR

**Figure 19:**
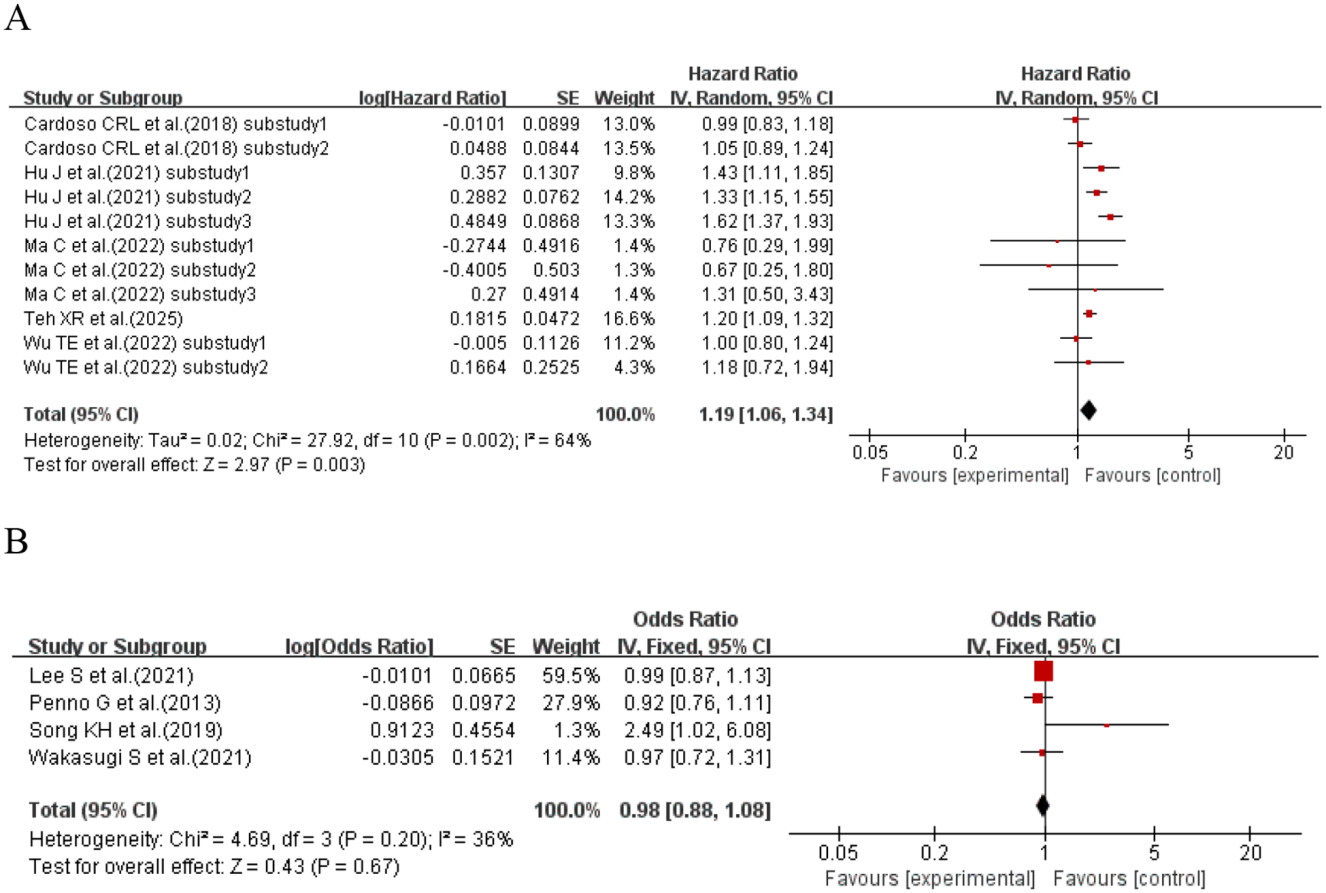
Forest plot showing the incidence of diabetic retinopathy, including **(A)** Hazard Ratio (HR) and **(B)** Odds Ratio (OR) for HbA1c variability measured by HbA1c-SD in patients with T2DM based on published reports.

When the effect size was HR, five studies (12, 63, 68, 75, 79) (with a total of 11 sub-studies) explored the association between SD and retinopathy in T2DM patients. The results showed that compared with T2DM patients with lower SD, those with higher SD had a 19% increased risk of developing retinopathy (HR = 1.19, 95% CI: 1.06–1.34, p = 0.003). When the effect size was OR, four studies (59, 71, 72, 74) explored the association between SD and retinopathy in T2DM patients, and the results failed to support an association between SD and retinopathy risk in T2DM patients (OR = 0.98, 95% CI: 0.88–1.08, p = 0.67). See **Figure 19** for details.

### 3.4 Subgroup analyses

Subgroup analysis of diabetic kidney disease showed that in T1DM patients, both CV and SD of HbA1c variability were significantly associated with the incidence of kidney disease. This association was more consistent in subgroups with proteinuria occurrence, eGFR deterioration outcomes, longer follow-up duration, and retrospective study design. In T2DM patients, higher HbA1c variability was significantly associated with the risk of kidney disease occurrence and mortality. The HR/OR values corresponding to SD and CV were all >1 across different subgroups, indicating robust results. When grouped by sample size, the SD-HR was 1.31 (95% CI: 1.18–1.45) in studies with ≥1,000 cases and 1.17 (95% CI: 1.00–1.38) in studies with<1,000 cases. When grouped by kidney disease outcomes, both SD and CV were significantly associated with the risk of proteinuria occurrence and eGFR deterioration; moreover, the risk of kidney disease-related mortality in the highest CV quantile (Q4/Q5) was significantly higher than that in the lowest quantile (Q1). Subgroup analysis of DR showed that in T1DM patients, the adjusted SD was significantly associated with DR risk, and the consistency was stronger in subgroups of the Asian population, retrospective studies, and sample size<1,000 cases. In T2DM patients, the unadjusted SD was associated with DR risk (HR = 1.21, 95% CI: 1.07–1.37); the association was more significant in the Asian population, and the risk in the highest CV quantile (Q4/Q1) was higher (HR = 1.61). After stratification by follow-up duration, subgroup analysis of diabetic nephropathy outcomes showed that for patients with T1DM and T2DM, the heterogeneity statistic of HRs or ORs corresponding to each HbA1c variability indicator (SD, CV, HVS, and HGI) exhibited a decreasing trend. Meanwhile, in the analysis of diabetic retinopathy outcomes, for patients with T2DM, after stratifying the SD-HR indicator by follow-up duration, the I^2^ values of both subgroups decreased from 64% to approximately 30%, with a significant reduction in research heterogeneity.

Subgroup analysis revealed that the heterogeneity of the study mainly stemmed from differences in clinical factors and methodologies. Disparities in follow-up duration, outcome indicators, and study types across included studies led to substantial variations in study designs; after homogenizing study types across different dimensions, the heterogeneity was reduced. In addition, stratification by region also resulted in decreased heterogeneity, which may be attributed to significant differences in genetic backgrounds, lifestyles, and blood glucose management standards among populations from different regions.

### 3.5 Sensitivity analysis

Sensitivity analysis for kidney disease incidence in T1DM patients showed that after excluding a single study, the heterogeneity of the association between glucose variability and T1DM kidney disease risk could be reduced. Sensitivity analysis for kidney disease incidence in T2DM patients indicated that the association between glucose variability and kidney disease progression risk was generally robust. After excluding specific studies or adjusting statistical models, there was no substantial change in the direction and significance of the pooled effect size, and the 95% CI did not include 1. Furthermore, after excluding studies with short follow-up duration, the heterogeneity was significantly reduced. In the sensitivity analysis of CV and kidney disease mortality in DM patients, after excluding Sub-study 5 of the sub-study of Afghahi H et al., with an abnormally elevated HR, there was no significant heterogeneity in the study (I^2^ = 0%, p = 0.54). Although the HR fluctuated slightly, the 95% CI of the two results showed a high degree of overlap, indicating good stability. In addition, the sensitivity analysis of hemoglobin A1c variability and diabetic retinopathy risk showed that the results remained robust after excluding a single study. See **Appendix A Tables 4–6, Appendix B Table 2** for details.

### 3.6 Publication bias

Publication bias in this study was assessed using the funnel plot and Egger’s test, with the trim-and-fill method applied for bias correction. The analysis showed no significant publication bias in the SD-HR for T2DM renal progression and CV-HR for T2DM kidney mortality rate (Egger’s test, p = 0.389/0.160 > 0.05).

Publication bias existed in the CV-HR for T2DM renal progression (Egger’s test, p = 0.009). It was estimated that four studies with weaker treatment effects may not have been included in the analysis. After supplementing these potentially missing studies, the confidence interval of the pooled effect size widened, leading to increased uncertainty in the results. Although the point estimate after filling in the missing data still showed statistical significance, its effect direction was inconsistent with the expectation of the preset model, and the resulting stability needs further verification. In the future, it is necessary to expand the sample size and include unpublished studies to improve the robustness of the conclusion. Publication bias existed in the CV-OR for T2DM renal progression (Egger’s test, p< 0.05). After imputing two missing studies, the pooled odds ratio decreased from 0.439 to 0.352, and the 95% CI changed from (0.214, 0.663) to (0.119, 0.585). Although publication bias increased uncertainty, it did not alter the overall conclusion that “elevated CV increases the risk of renal progression in T2DM patients”. Publication bias existed in the SD-HR for T1DM renal progression (Egger’s test, p = 0.036). After imputing one missing study, the pooled effect size changed from −0.021 to 0.038. The 95% CI included 0 both before and after adjustment, and the main conclusion remained unchanged. See **Appendix A Tables 7, 8** for details.

## 4 Discussion

This meta-analysis aimed to systematically evaluate the association between HbA1c variability and diabetes, as well as its potential clinical significance. The core value of this study lies in the fact that HbA1c variability, as a modifiable risk factor, is expected to provide new quantitative indicators and a theoretical basis for optimizing the management strategies of patients with DM, thereby addressing the limitations of traditional static blood glucose assessment. The analysis results showed that regardless of whether SD, CV, HVS, or HGI was used as the quantitative indicator, the long-term variability of HbA1c levels was significantly associated with the risk of microvascular-related diseases in DM patients. This suggests that blood glucose fluctuations may be one of the important driving factors for microvascular damage.

A review of previous studies shows that there has been extensive exploration into the association between HbA1c variability (measured by SD and CV) and T2DM-related complications: a meta-analysis involving 12 studies (21) and 44,662 T2DM patients confirmed that higher HbA1c-SD and HbA1c-CV were significantly associated with an increased risk of retinopathy in patients. Another comprehensive analysis covering 23 studies (23) further indicated that long-term HbA1c variability (SD/CV) was positively associated with macrovascular complications, microvascular complications, and all-cause mortality in T2DM patients. These findings fully highlight the clinical value of HbA1c variability in predicting T2DM-related adverse outcomes. Therefore, HbA1c-SD and HbA1c-CV have strong universality in evaluating blood glucose and related complications in diabetic patients. This study also indicates that for every 1% increase in the SD of HbA1c (i.e., HbA1c-per 1% increase in SD), both the risk of kidney disease development and mortality risk in diabetic patients will increase accordingly.

In the present study, DKD and DR were selected as the research objects for the risk prediction of HbA1c variability, while diabetic peripheral neuropathy (DPN), another type of diabetic microangiopathy, was excluded. The reasons for this selection are as follows: first, in clinical practice, the diagnostic criteria for DPN exhibit substantial variability with a lack of unified quantitative standards, which not only hinders data pooling but also may introduce more uncontrollable research heterogeneity; a study focusing on the predictors of DPN demonstrated that the 27 included studies adopted diverse definitions of DPN, which mainly consisted of comprehensive clinical assessments of symptoms and signs, monofilament testing, standardized rating scales, and nerve conduction function tests (80). Second, existing research has indicated that the levels of objective clinical indicators, such as the neutrophil-to-lymphocyte ratio (NLR), are higher in patients with T2DM complicated by DPN than in those without DPN, suggesting that NLR has predictive value for DPN risk, yet inconsistent conclusions have been reported across different regions (81). In contrast, the clinical screening and monitoring pathways for DKD and DR are relatively standardized: specifically, DKD can be clearly defined using objective laboratory parameters such as the eGFR and urine albumin-to-creatinine ratio, while DR allows for standardized grading via fundus examinations, and clarifying the association between these two complications and HbA1c variability is conducive to the early prevention and control of DKD and DR.

HGI is defined as the difference between the observed HbA1c and the predicted HbA1c in the linear regression equation fitted based on FPG (82). HGI can relatively intuitively reflect the blood glucose fluctuation in patients and quantify the change in the relationship between HbA1c and blood glucose concentration (83). Multiple studies have shown that HGI can predict the risk of diabetic complications, including mortality and microvascular complications (82–85). Furthermore, high HGI is closely associated with the risk of developing diabetic microangiopathy in the population. In this study, we evaluated the correlation between HGI and the incidence of kidney disease in patients with T2DM. The results showed that high HGI was closely associated with decreased renal function in diabetic patients, suggesting that HGI may be an independent risk factor for patients with diabetic kidney disease. In clinical practice, a large amount of data can be used to further calculate HGI and explore its correlation. HVS is a new method for evaluating HbA1c variability proposed by Forbes et al. in 2018. HVS is calculated as the percentage of all individual HbA1c measurements where the change in HbA1c level exceeds 0.5% (5.5 mmol/mol) (86). This HbA1c measurement indicator has higher clinical translatability; therefore, using HVS offers several advantages over SD and CV. It can well reflect the frequency of HbA1c variability, but it tends to overlook the magnitude of variability. In this study, SD, CV, HGI, and HVS were combined to systematically demonstrate the impact of HbA1c variability on outcomes.

In this study, the association between HbA1c variability and diabetic kidney disease, as well as diabetic retinopathy, reached a significant level. Given that most of the included literature supports the relevance to vascular injury risk, the significance of the overall effect is thus reflected. For patients with T1DM, however, this study showed that the associations between blood glucose variability (measured by SD, CV, and HVS) and diabetic complications were relatively weak. This is considered to be related to the insufficient reserve of literature data on the association between HbA1c variability and T1DM complications in previous studies. The sample size and effect size information provided by existing studies are limited, which cannot meet the needs of further in-depth analysis, and ultimately leads to the limitation of statistical test power for the association effect in this population.

In this study, OR for HbA1c variability was estimated using datasets, and a significant overall effect was observed. However, when assessing HbA1c-related risks, there were notable differences between the results of OR and HR, with the association between HR and outcomes being particularly more significant. OR is used to measure the strength of the association between exposure and outcome. Although it can reflect the overall effect, it tends to overestimate the actual risk and exhibits a static nature, making it unable to capture the temporal dynamic changes in event incidence rates (87). In contrast, HR focuses on the temporal differences in event occurrence and more intuitively reflects the impact of exposure on the timing of event onset. Typically estimated via the Cox proportional hazards model, HR is suitable for analyzing the effect of covariates on the “time to first event” and can characterize the dynamic process of risk changes over time. It is a measure of instantaneous risk intensity (88).

This study is the first meta-analysis to explore the association between multiple HbA1c variability indicators (SD, CV, HVS, and HGI) and cardiovascular disease-related risk from multiple perspectives. A total of 45 cohort studies were included, with the NOS quality scores ranging from 6 to 8. This indicates that the included studies have an overall high methodological quality, which enhances the credibility of the results. Observation of the data distribution in the forest plots showed that although heterogeneity existed in some studies, the effect sizes of the vast majority of studies fell outside the null effect line (HR = 1). This distribution characteristic suggests that, overall, there is consistency in the association between the exposure factor (HbA1c variability) and the increased outcome risk among the study subjects. Furthermore, it is important to note that during the data inclusion process of this study, effect estimates derived from different statistical models within the same original study were also considered. While this data inclusion method enriched the analysis sample size, it may also increase the dispersion of the overall data to a certain extent, thereby affecting the heterogeneity results. Given that the onset of DKD and DR is closely associated with the natural progression of diabetes, varying follow-up durations across studies will lead to the observation of different clinical outcomes. Inconsistencies in the definitions, measurement tools, or cutoff values of the outcome indicators used across included studies directly result in the incomparability of effect sizes, thus contributing to high levels of heterogeneity. This study incorporated both retrospective and prospective cohort studies. Retrospective studies rely on existing historical data, which may fail to capture key confounding variables, thereby introducing a higher risk of measurement errors and residual confounding. This could potentially lead to systematic deviations between the effect sizes estimated by retrospective studies and those derived from prospective studies. To reduce heterogeneity, subgroup analyses were performed in this study, which demonstrated that stratification by region, sample size, study type, follow-up duration, outcome indicators, and blood glucose variability quartiles could effectively reduce research heterogeneity.

The results of the subgroup analysis showed that when the HbA1c variability rate was in the high quantile (Q4/Q1), the risk of diabetic complications increased significantly. From a physiological mechanism perspective, a high quantile of HbA1c variability essentially reflects greater amplitude and higher frequency of blood glucose fluctuations. Such repeated blood glucose fluctuations cause cumulative damage to target tissues such as blood vessels and nerves, and the degree of pathological harm is even greater than that of a persistently stable hyperglycemic state. Ultimately, this leads to a significant increase in the risk of diabetic complications (89). Further analysis revealed that high-quantile HbA1c variability can directly impair the normal physiological function of vascular endothelial cells while activating signaling pathways related to oxidative stress (90, 91). This mechanism is particularly prominent in diabetic retinopathy, driving the progression of the disease from the early stage to the middle and advanced stages (92, 93). Furthermore, persistent exposure to high HbA1c variability percentiles can induce the “metabolic memory effect”, which refers to a phenomenon where pathological changes persist even after blood glucose levels return to normal following a hyperglycemic episode. These pathological alterations increase the risk of long-term complications (94). Even if blood glucose levels are effectively controlled through subsequent interventions, studies have shown that the incidence of severe nephropathy and retinopathy decreases within 10 years after the end of intensive treatment, whereas the “metabolic memory effect” can persist for approximately 10 years (95). As an indicator reflecting the characteristics of long-term glycemic fluctuations, HbA1c variability may exert its association with the development and progression of DKD and DR through the molecular mechanisms underlying the “metabolic memory effect”. DNA methylation is a key mediating mechanism of the “metabolic memory effect” in T1DM, and the methylation status of key CpG sites can perpetuate the pathological manifestations induced by previous hyperglycemia over the long term (96).

Compared with microangiopathy, a well-recognized diabetic complication, the extensive impact of diabetes on the systemic vascular system is also a key factor contributing to the elevated risk of adverse outcomes in patients. As an important component of metabolic syndrome—a cluster of metabolic disorders characterized by central obesity, dyslipidemia, hypertension, and insulin resistance (97)—diabetes presents a pathological feature of gradient superposition of effects. Specifically, the greater the severity of abnormalities in each component and the larger the number of involved components, the more pronounced the disruption to the body’s metabolic homeostasis, which in turn synergistically increases the risk of obesity, cardiovascular, and cerebrovascular diseases (98). Studies have indicated (99) that during the progression of T2DM, hyperinsulinemia acts as a key pathological driver of disease advancement. Sustained hyperinsulinemia promotes the accumulation of advanced glycation end products (AGEs), which in turn induce excessive production of reactive oxygen species (ROS). Excessive ROS triggers oxidative stress responses, causing tissue damage and vascular endothelial dysfunction and, ultimately, greatly increasing the susceptibility to ischemic stroke.

Based on this, proactive preventive strategies are of great importance. Interventions, including optimizing sleep patterns, developing relevant sleep protectants, regulating blood–brain barrier permeability, and inhibiting systemic and local inflammatory responses, can effectively target the hyperinsulinemia–AGE–ROS pathway, thereby reducing the risk of stroke in T2DM patients complicated with metabolic syndrome. At the clinical level, clinicians need to formulate individualized management plans based on patients’ metabolic characteristics, including core measures such as continuous monitoring of insulin resistance levels and targeted regulation of insulin concentrations. Meanwhile, at the community level, the key lies in optimizing treatments through four core measures. First, population screening and risk stratification can be carried out to identify individuals with prediabetes and those at high risk of metabolic syndrome at an early stage. Second, health education and lifestyle interventions should be promoted to improve residents’ awareness of knowledge related to metabolic health. Third, a collaborative network and data-sharing platform within medical consortia can be established to facilitate the practical implementation of individualized treatment plans. Fourth, a long-term follow-up and compliance management system should be refined to ensure the sustainability of intervention measures. Through the synergistic collaboration between clinical practice and community care, full-process coverage from screening of high-risk groups and early intervention to long-term management can be achieved, thereby maximizing the reduction of the incidence risk of metabolic syndrome-related diabetes and stroke.

This study has the following limitations: first, differences existed in the detection frequency, time interval, and measurement equipment/methods of HbA1c across the original studies, which may have introduced study heterogeneity. Second, some potential confounding factors were fully adjusted for, which may have interfered with the effect estimation. Third, all included evidence was derived from observational studies, so the results of this paper only revealed a statistical association rather than a causal relationship.

In conclusion, this study demonstrates that HbA1c variability is positively correlated with the incidence of microvascular-related diseases and mortality progression in diabetic patients. Individualized treatment based on HbA1c variability is expected to become a key component in the practice of precision diabetes care, providing important references for optimizing the prevention and management of microvascular complications and improving patient prognosis.

## 5 Conclusion

This study confirmed through a meta-analysis that HbA1c variability is positively correlated with the risk of adverse renal events and retinal diseases in diabetic patients. Therefore, HbA1c variability may play an important and promising role in guiding blood glucose control targets for diabetic patients and predicting the progression of microvascular complications.

## Data Availability

The original contributions presented in the study are included in the article/**Supplementary Material**. Further inquiries can be directed to the corresponding authors.
