# Supplementary material for: Association between glycated hemoglobin variability and risk of diabetic kidney disease and diabetic retinopathy in diabetic patients: a systematic review and meta-analysis
Source: Front Endocrinol (Lausanne). 2026 Jan 30;17:1703190. doi: 10.3389/fendo.2026.1703190 (PMC12901347; doi:10.3389/fendo.2026.1703190)
Supplement: Supplementary file 3 [file DataSheet3.pdf]

Pubmed

(((("Diabetic Retinopathy"[Mesh]) OR (((Diabetic Retinopathies[Title/Abstract]) OR (Retinopathies, Diabetic[Title/Abstract])) OR (Retinopathy, Diabetic[Title/Abstract]))) OR ((("Retinal Diseases"[Mesh]) OR (((Disease, Retinal[Title/Abstract]) OR (Diseases, Retinal[Title/Abstract])) OR (Retinal Disease[Title/Abstract]))) AND (("Diabetes Mellitus"[Mesh]) OR (((((((Diabetes Insipidus[Title/Abstract]) OR (Diet, Diabetic[Title/Abstract])) OR (Prediabetic State[Title/Abstract])) OR (Scleredema Adultorum[Title/Abstract])) OR (Glycation End Products, Advanced[Title/Abstract])) OR (Glucose Intolerance[Title/Abstract])) OR (Gastroparesis[Title/Abstract]))))) AND (((Glycated Hemoglobin[MeSH]) OR ((Glycated Hemoglobin*[Title/Abstract]) OR (Hemoglobin*, Glycated[Title/Abstract]) OR (Hemoglobin*, Glycosylated[Title/Abstract]) OR (Glycosylated Hemoglobin*[Title/Abstract]) OR (Glycated Hemoglobin A1c[Title/Abstract]) OR (Hemoglobin A1c, Glycated[Title/Abstract]) OR (Glycosylated Hemoglobin A1c[Title/Abstract]) OR (Hemoglobin A1c, Glycosylated[Title/Abstract]) OR (Glycated Hemoglobin A[Title/Abstract]) OR (Hemoglobin A, Glycated[Title/Abstract]) OR (Glycosylated Hemoglobin A[Title/Abstract]) OR (Hemoglobin A, Glycosylated[Title/Abstract]) OR (HbA1c[Title/Abstract]) OR (Glycohemoglobin*[Title/Abstract]) OR (Glycohemoglobin A[Title/Abstract]) OR (Fructated Hemoglobin*[Title/Abstract]) OR (Hemoglobin*, Fructated[Title/Abstract]))) AND ((HbA(1c) variability) OR (HbA(1c) variation) OR (variability) OR (variation)))) OR (((("Kidney Diseases"[Mesh]) OR ("Proteinuria"[Mesh])) OR ((Diabetic Nephropath*[Title/Abstract]) OR (Nephropath*, Diabetic[Title/Abstract]) OR (Diabetic Kidney Disease*[Title/Abstract]) OR (Kidney Disease*, Diabetic[Title/Abstract]) OR (Diabetic Glomerulosclerosis[Title/Abstract]) OR (Glomerulosclerosis, Diabetic[Title/Abstract]) OR (Intracapillary Glomerulosclerosis[Title/Abstract]) OR (Nodular Glomerulosclerosis[Title/Abstract]) OR (Glomerulosclerosis, Nodular[Title/Abstract]) OR (Kimmelstiel-Wilson Syndrome[Title/Abstract]) OR (Kimmelstiel Wilson Syndrome[Title/Abstract]) OR (Syndrome, Kimmelstiel-Wilson[Title/Abstract]) OR (Kimmelstiel-Wilson Disease[Title/Abstract]) OR (Kimmelstiel Wilson Disease[Title/Abstract]) OR (Renal Insufficienc*[Title/Abstract]) OR (Kidney Insufficienc*[Title/Abstract]) OR (Kidney Disease*[Title/Abstract]) OR (Renal Disease*[Title/Abstract]) OR (Albuminuria[Title/Abstract]) OR (Proteinuria[Title/Abstract]))) AND (((Glycated Hemoglobin[MeSH]) OR ((Glycated Hemoglobin*[Title/Abstract]) OR (Hemoglobin*, Glycated[Title/Abstract]) OR (Hemoglobin*, Glycosylated[Title/Abstract]) OR (Glycosylated Hemoglobin*[Title/Abstract]) OR (Glycated Hemoglobin A1c[Title/Abstract]) OR (Hemoglobin A1c, Glycated[Title/Abstract]) OR (Glycosylated Hemoglobin A1c[Title/Abstract]) OR (Hemoglobin A1c, Glycosylated[Title/Abstract]) OR (Glycated Hemoglobin A[Title/Abstract]) OR (Hemoglobin A, Glycated[Title/Abstract]) OR (Glycosylated Hemoglobin A[Title/Abstract]) OR (Hemoglobin A, Glycosylated[Title/Abstract]) OR (HbA1c[Title/Abstract]) OR (Glycohemoglobin*[Title/Abstract]) OR (Glycohemoglobin A[Title/Abstract]) OR (Fructated Hemoglobin*[Title/Abstract]) OR (Hemoglobin*, Fructated[Title/Abstract]))) AND ((HbA(1c) variability) OR (HbA(1c) variation) OR (variability) OR (variation))))

Total：1554

Web of science

1: TS=(((glycated hemoglobin*) OR (hemoglobin*, glycated) OR (hemoglobin*, glycosylated) OR (glycosylated hemoglobin*) OR (glycated hemoglobin a1c) OR (hemoglobin a1c, glycated) OR (glycosylated hemoglobin a1c) OR (hemoglobin a1c, glycosylated) OR (glycated hemoglobin a) OR (hemoglobin a,glycated) OR (glycosylated hemoglobin a) OR (hemoglobin a, glycosylated) OR (hba1c) OR (glycohemoglobin*) OR (glycohemoglobin a) OR (frustated hemoglobin*) OR (hemoglobin*, frustated)))

2: TS=(((HbA(1c) variability) OR (HbA(1c) variation) OR (variability) OR (variation)))

3: (#1) AND #2

8：TS=((diabetic nephropath*) OR (nephropath*, diabetic) OR (diabetic kidney disease*) OR (kidney disease*, diabetic) OR (diabetic glomerulosclerosis) OR (glomerulosclerosis, diabetic) OR (intracapillary glomerulosclerosis) OR (nodular glomerulosclerosis) OR (glomerulosclerosis, nodular) OR (kimmelstiel-wilson syndrome) OR (kimmelstiel wilson syndrome) OR (syndrome, kimmelstiel-wilson) OR (kimmelstiel-wilson disease) OR (kimmelstiel wilson disease) OR (renal insufficienc*) OR (kidney insufficienc*) OR (kidney disease*) OR (renal disease*) OR (albuminuria) OR (proteinuria))

9：(((TS=(retinal disease)) OR TS=(Disease, Retinal)) OR TS=(Diseases, Retinal)) OR TS=(Retinal Disease)

10: (((((((TS=(Diabetes Mellitus*)) OR TS=(Diabetes Insipidus)) OR TS=(Diet, Diabetic)) OR TS=(Prediabetic State)) OR TS=(Scleredema Adultorum)) OR TS=(Glycation End Products, Advanced)) OR TS=(Glucose Intolerance)) OR TS=(Gastroparesis)

11: (#9) AND #10

12: (((TS=(Diabetic Retinopathy*)) OR TS=(Diabetic Retinopathies)) OR TS=(Retinopathies, Diabetic)) OR TS=(Retinopathy, Diabetic)

13: (#11) OR #12

14：(#13) AND #3

15：(#8) AND #3

16：#14OR#15

Total：507

Cochorane library

#1 MeSH descriptor: [Glycated Hemoglobin] explode all trees

#2 (((glycated hemoglobin*) OR (hemoglobin*, glycated) OR (hemoglobin*, glycosylated) OR (glycosylated hemoglobin*) OR (glycated hemoglobin a1c) OR (hemoglobin a1c, glycated) OR (glycosylated hemoglobin a1c) OR (hemoglobin a1c, glycosylated) OR (glycated hemoglobin a) OR (hemoglobin a,glycated) OR (glycosylated hemoglobin a) OR (hemoglobin a, glycosylated) OR (hba1c) OR (glycohemoglobin*) OR (glycohemoglobin a) OR (fructated hemoglobin*) OR (hemoglobin*, fructated))):ti,ab,kw (Word variations have been searched)

#3 #1 or #2

#4 ((HbA(1c) variability) OR (HbA(1c) variation) OR (variability) OR (variation))

#5 #3 and #4

#6 MeSH descriptor: [Diabetes Mellitus] explode all trees

#7 ((Diabetes Insipidus) OR (Diet, Diabetic) OR (Prediabetic State) OR (Scleredema Adultorum) OR (Glycation End Products, Advanced) OR (Glucose Intolerance) OR (Gastroparesis)):ti,ab,kw (Word variations have been searched)

#8 #6 or #7

#15 MeSH descriptor: [Glycated Hemoglobin] explode all trees

#16 MeSH descriptor: [Proteinuria] explode all trees

#17 MeSH descriptor: [Diabetic Nephropathies] explode all trees

#18 (((glycated hemoglobin*) OR (hemoglobin*, glycated) OR (hemoglobin*, glycosylated) OR (glycosylated hemoglobin*) OR (glycated hemoglobin a1c) OR (hemoglobin a1c, glycated) OR (glycosylated hemoglobin a1c) OR (hemoglobin a1c, glycosylated) OR (glycated hemoglobin a) OR (hemoglobin a,glycated) OR (glycosylated hemoglobin a) OR (hemoglobin a, glycosylated) OR (hba1c) OR (glycohemoglobin*) OR (glycohemoglobin a) OR (fructated hemoglobin*) OR (hemoglobin*, fructated))):ti,ab,kw (Word variations have been searched)

#19 #15 or #16 or #17 or #18

#20 MeSH descriptor: [Retinal Diseases] explode all trees

#21 MeSH descriptor: [Diabetic Retinopathy] explode all trees

#22 ((Disease, Retinal) OR (Diseases, Retinal) OR (Retinal Disease)):ti,ab,kw (Word variations have been searched)

#23 #20 OR #22

#24 ((Diabetic Retinopathies) OR (Retinopathies, Diabetic) OR (Retinopathy, Diabetic)):ti,ab,kw (Word variations have been searched)

#25 #21 OR #24

#26 #23 AND #8

#27 #25 OR #26

#28 #19 AND #5

#29 #27 AND #5

#30 #28 OR #29

Total：2011

Embase
#1. 'glycated hemoglobin'/exp
#2. 'glycated hemoglobin*':ti,ab,kw OR 'hemoglobin*, glycated':ti,ab,kw OR 'hemoglobin*, glycosylated':ti,ab,kw OR 'glycosylated hemoglobin*':ti,ab,kw OR 'glycated hemoglobin a1c':ti,ab,kw OR 'hemoglobin a1c, glycated':ti,ab,kw OR 'glycosylated hemoglobin a1c':ti,ab,kw OR 'hemoglobin a1c, glycosylated':ti,ab,kw OR 'glycated hemoglobin a':ti,ab,kw OR 'hemoglobin a, glycated':ti,ab,kw OR 'glycosylated hemoglobin a':ti,ab,kw OR 'hemoglobin a, glycosylated':ti,ab,kw OR 'hba1c':ti,ab,kw OR 'glycohemoglobin*':ti,ab,kw OR 'glycohemoglobin a':ti,ab,kw OR 'fructated hemoglobin*':ti,ab,kw OR 'hemoglobin*, fructated':ti,ab,kw
#3. #1 OR #2
#4. (hba:ti,ab,kw AND 1c:ti,ab,kw AND variability:ti,ab,kw OR hba:ti,ab,kw) AND 1c:ti,ab,kw AND variation:ti,ab,kw OR 'variability':ti,ab,kw OR 'variation':ti,ab,kw
#5. #3 AND #4
#6. 'diabetes mellitus'/exp
#7. 'diabetes':ti,ab,kw OR 'diabetic':ti,ab,kw OR 'diabets':ti,ab,kw OR 'unspecified diabetes mellitus':ti,ab,kw OR 'diabetes mellitus':ti,ab,kw OR 'diabetes insipidus':ti,ab,kw OR 'diet, diabetic':ti,ab,kw OR 'prediabetic state':ti,ab,kw OR 'scleredema adultorum':ti,ab,kw OR 'glycation end products, advanced':ti,ab,kw OR 'glucose intolerance':ti,ab,kw OR 'gastroparesis':ti,ab,kw
#8. #6 OR #7
#16. 'kidney disease'/exp
#17. 'proteinuria'/exp
#18. 'diabetic nephropathy'/exp
#19. 'diabetic nephropath*':ti,ab,kw OR 'nephropath*, diabetic':ti,ab,kw OR 'diabetic kidney disease*':ti,ab,kw OR 'kidney disease*, diabetic':ti,ab,kw OR 'diabetic glomerulosclerosis':ti,ab,kw OR 'glomerulosclerosis, diabetic':ti,ab,kw OR 'intracapillary glomerulosclerosis':ti,ab,kw OR 'nodular glomerulosclerosis':ti,ab,kw OR 'glomerulosclerosis, nodular':ti,ab,kw OR 'kimmelstiel-wilson syndrome':ti,ab,kw OR 'kimmelstiel wilson syndrome':ti,ab,kw OR 'syndrome, kimmelstiel-wilson':ti,ab,kw OR 'kimmelstiel-wilson disease':ti,ab,kw OR 'kimmelstiel wilson disease':ti,ab,kw OR 'renal insufficienc*':ti,ab,kw OR 'kidney insufficienc*':ti,ab,kw OR 'kidney disease*':ti,ab,kw OR 'renal disease*':ti,ab,kw OR 'albuminuria':ti,ab,kw OR 'proteinuria':ti,ab,kw
#20. #16 OR #17 OR #18 OR #19
#21. 'retina disease'/exp
#22. 'retinal diseases':ti,ab,kw OR 'retina disease':ti,ab,kw OR 'disease, retinal':ti,ab,kw OR 'diseases, retinal':ti,ab,kw
#23. #21 OR #22
#24. #8 AND #23
#25. 'diabetic retinopathy'/exp
#26. 'diabetes mellitus retinopathy':ti,ab,kw OR 'diabetes retinopathy':ti,ab,kw OR 'diabetic retinitis':ti,ab,kw OR 'diabetic retinopathies':ti,ab,kw OR 'retinopathia diabetica':ti,ab,kw OR 'retinopathy in diabetes':ti,ab,kw OR 'diabetic retinopathy':ti,ab,kw OR 'retinopathies, diabetic':ti,ab,kw OR 'retinopathy, diabetic':ti,ab,kw

#27. #25 OR #26
#28. #24 OR #27
#29 #20 AND #5
#30 #28 AND #5
#31 #29 OR #30
Total：1788
