# Supplementary material for: Association between glycated hemoglobin variability and risk of diabetic kidney disease and diabetic retinopathy in diabetic patients: a systematic review and meta-analysis
Source: Front Endocrinol (Lausanne). 2026 Jan 30;17:1703190. doi: 10.3389/fendo.2026.1703190 (PMC12901347; doi:10.3389/fendo.2026.1703190)
Supplement: Supplementary file 4 [file DataSheet4.docx]

**List of Abbreviation**

| **Abbreviation** | **Full name** |
| --- | --- |
| Adj-SD | Adjusted Standard Deviation |
| CI | Confidence Interval |
| CIs | Confidence Intervals |
| CKD | Chronic Kidney Disease |
| CKD-EPI | Chronic Kidney Disease Epidemiology Collaboration |
| CV | Coefficient of Variation |
| DKD | Diabetic Kidney Disease |
| DM | Diabetes Mellitus |
| DR | Diabetic Retinopathy |
| ECM | Extracellular Matrix |
| eGFR | Estimated Glomerular Filtration Rate |
| Embase | Excerpta Medica DataBASE |
| ESRD | End-Stage Renal Disease |
| FBG | Fasting Blood Glucose |
| FPG | Fasting Plasma Glucose |
| GAD-Ab | Glutamic Acid Decarboxylase Antibody |
| HbA1c | Glycated Hemoglobin |
| HGI | Hemoglobin Glycation Index |
| HR | Hazard Ratio |
| HRs | Hazard Ratios |
| HVS | Glycemic Variability Score |
| IAA | Insulin Autoantibody |
| ICA | Islet Cell Antibody |
| IDF | International Diabetes Federation |
| Ins | Insulin |
| IRMAs | Intraretinal Microvascular Abnormalities |
| JSN | Japanese Society of Nephrology |
| MDRD | Modification of Diet in Renal Disease |
| MeSH | Medical Subject Headings |
| NOS | Newcastle-Ottawa Scale |
| NPDR | Non-Proliferative Diabetic Retinopathy |
| OGTT | Oral Glucose Tolerance Test |
| OR | Odds Ratio |
| ORs | Odds Ratios |
| PDR | Proliferative Diabetic Retinopathy |
| PubMed | Public Medicine |
| RRs | Relative Risks |
| RRT | Renal Replacement Therapy |
| SD | Standard Deviation |
| T1DM | Type 1 Diabetes |
| T2DM | Type 2 Diabetes |
| UACR | Urine Albumin-to-Creatinine Ratio |
| Web of Science | Web of Science Core Collection |
| ZnT8-Ab | Zinc Transporter 8 Antibody |
