# Supplementary material for: Association between glycated hemoglobin variability and risk of diabetic kidney disease and diabetic retinopathy in diabetic patients: a systematic review and meta-analysis
Source: Front Endocrinol (Lausanne). 2026 Jan 30;17:1703190. doi: 10.3389/fendo.2026.1703190 (PMC12901347; doi:10.3389/fendo.2026.1703190)
Supplement: Supplementary file 5 [file Table1.docx]

**Appendix A**

**Table 1**  Subgroup Analysis of the CV for Kidney Mortality Rate

| Subgroup | Studies | Pooled HR (95% CI) | *I^2^* (*p* value) |
| --- | --- | --- | --- |
| ***CV-T2DM*** | | | |
| **Sample Size** | | | |
| ＜10000 | Yang YF et al.(2015) substudy1/  Yang YF et al.(2015) substudy2/  Yang YF et al.(2015) substudy3/  Yang YF et al.(2015) substudy4/  Slieker RC et al.(2019) substudy1/  Slieker RC et al.(2019) substudy2/  Slieker RC et al.(2019) substudy3/  Slieker RC et al.(2019) substudy4 | 1.13 (1.03, 1.24） | *I^2^* = 74%（*P* < 0.01） |
| ≥10000 | Teh XR et al.(2025) substudy1/  Teh XR et al.(2025) substudy2 | 0.77 (0.55, 1.09) | *I^2^* = 2%（*P=*0.31） |
| **Time** | | | |
| median of 9 years | Slieker RC et al.(2019) substudy1/  Slieker RC et al.(2019) substudy2/  Slieker RC et al.(2019) substudy3/  Slieker RC et al.(2019) substudy4 | 1.17 (1.05, 1.29） | *I^2^* = 84%（*P*＜0.01） |
| ≧median of 9 years | Teh XR et al.(2025) substudy1/  Teh XR et al.(2025) substudy2 | 0.77 (0.55, 1.09） | *I^2^* = 2%（*P* = 0.31） |
| **Quartile** | | | |
| Q2/Q1 | Teh XR et al.(2025) substudy1/  Slieker RC et al.(2019) substudy1/  Yang YF et al.(2015) substudy1 | 0.94 (0.77, 1.15） | *I^2^* = 52%（*P* = 0.13） |
| Q3/Q1 | Teh XR et al.(2025) substudy2/  Slieker RC et al.(2019) substudy2/  Yang YF et al.(2015) substudy2 | 1.08 (0.99, 1.17） | *I^2^* =0%（*P* =0.40） |
| Q4/Q1 | Slieker RC et al.(2019) substudy3/  Yang YF et al.(2015) substudy3 | 1.18 (1.10, 1.28） | *I^2^* =2%（*P* = 0.31） |
| Q5/Q1 | Slieker RC et al.(2019) substudy4/  Yang YF et al.(2015) substudy4 | 1.34 (1.24, 1.46） | *I^2^* =0%（*P* = 0.83） |

CV: coefficient of variation; HR: hazard ratio

**Table 2**  Subgroup analyses for the SD and CV of renal outcomes in patients with T1DM

| Subgroup | Studies | Pooled HR/OR (95% CI) | *I^2^* (*p* value) |
| --- | --- | --- | --- |
| ***SD-HR*** | | | |
| **Index** | | | |
| Adjusted SD | Bille N et al.(2021) substudy4/  Bille N et al.(2021) substudy5/  Bille N et al.(2021) substudy6 | 0.54 (0.37, 0.78） | *I^2^* = 0%（*P* = 0.69） |
| SD | Wadén J et al.(2009)/  Romero-Aroca P et al.(2021)/  Bille N et al.(2021) substudy1/  Bille N et al.(2021) substudy2/  Bille N et al.(2021) substudy3/  Muthukumar A et al.(2024) substudy1/  Muthukumar A et al.(2024) substudy2 | 1.24 (0.80, 1.94） | *I^2^* = 84%（*P* < 0.01） |
| **Renal Outcome** | | | |
| incidence of albuminuria | Romero-Aroca P et al.(2021)/  Wadén J et al.(2009) | 1.65 (1.19, 2.28） | *I^2^* = 62%（*P* = 0.11） |
| incidence of eGFR exacerbation | Muthukumar A et al.(2024) substudy1/  Muthukumar A et al.(2024) substudy2 | 2.63 (1.57, 4.41） | *I^2^* = 41%（*P* = 0.19） |
| composite outcomes | Bille N et al.(2021) substudy1/  Bille N et al.(2021) substudy2/  Bille N et al.(2021) substudy3/  Bille N et al.(2021) substudy4/  Bille N et al.(2021) substudy5/  Bille N et al.(2021) substudy6 | 0.56 (0.43, 0.73） | *I^2^* = 0%（*P* = 0.97） |
| **Sample Size** | | | |
| <1000 | Bille N et al.(2021) substudy1/  Bille N et al.(2021) substudy2/  Bille N et al.(2021) substudy3/  Bille N et al.(2021) substudy4/  Bille N et al.(2021) substudy5/  Bille N et al.(2021) substudy6/  Romero-Aroca P et al.(2021) | 0.66 (0.45, 0.98） | *I^2^* = 69%（*P* < 0.01） |
| ≥1000 | Wadén J et al.(2009)/  Muthukumar A et al.(2024) substudy1/  Muthukumar A et al.(2024) substudy2 | 2.23 (1.60, 3.12） | *I^2^* = 43%（*P* = 0.17） |
| **Area** | | | |
| Europe | Wadén J et al.(2009)/  Muthukumar A et al.(2024) substudy1/  Muthukumar A et al.(2024) substudy2/  Romero-Aroca P et al.(2021) | 1.95 (1.41, 2.71） | *I^2^* = 64%（*P* = 0.04） |
| Africa | Bille N et al.(2021) substudy1/  Bille N et al.(2021) substudy2/  Bille N et al.(2021) substudy3/  Bille N et al.(2021) substudy4/  Bille N et al.(2021) substudy5/  Bille N et al.(2021) substudy6 | 2.61 (1.54, 4.41） | *I^2^* = 88%（*P* < 0.01） |
| **Design** |  |  |  |
| prospective | Wadén J et al.(2009)/  Romero-Aroca P et al.(2021)/  Bille N et al.(2021) substudy1/  Bille N et al.(2021) substudy2/  Bille N et al.(2021) substudy3/  Bille N et al.(2021) substudy4/  Bille N et al.(2021) substudy5/  Bille N et al.(2021) substudy6 | 0.76 (0.49, 1.20） | *I^2^* = 85%（*P* < 0.01） |
| retrospective | Muthukumar A et al.(2024) substudy1/  Muthukumar A et al.(2024) substudy2 | 2.63 (1.57, 4.41） | *I^2^* = 41%（*P* = 0.19） |
| **Time** | | | |
| median of 8 years | Wadén J et al.(2009)/  Romero-Aroca P et al.(2021) | 1.65 (1.19, 2.28） | *I^2^* = 62%（*P* = 0.11） |
| ≧median of 8 years | Muthukumar A et al.(2024) substudy1/  Muthukumar A et al.(2024) substudy2 | 2.63 (1.57, 4.41） | *I^2^* = 41%（*P* = 0.19） |
| **Quartile** | | | |
| Q2/Q1 | Bille N et al.(2021) substudy1/  Bille N et al.(2021) substudy4/  Muthukumar A et al.(2024) substudy1 | 0.95 (0.45, 2.03） | *I^2^* = 78%（*P* = 0.01） |
| Q3/Q1 | Bille N et al.(2021) substudy2/  Bille N et al.(2021) substudy5/  Muthukumar A et al.(2024) substudy2 | 0.97 (0.26, 3.62） | *I^2^* =92%（*P* < 0.01） |
| Q4/Q1 | Bille N et al.(2021) substudy3/  Bille N et al.(2021) substudy6 | 0.51 (0.33, 0.81） | *I^2^* =0%（*P* = 0.77） |
| **SD-OR** |  |  |  |
| **Index** |  |  |  |
| Adjusted SD | Rosa LCGFD et al.(2019) substudy1/  Rosa LCGFD et al.(2019) substudy2 | 1.71 (0.51, 5.78） | *I^2^* = 57%（*P* = 0.13） |
| SD | Virk SA et al.(2016) substudy1/  Virk SA et al.(2016) substudy2/  Suh J et al.(2023) substudy1/  Suh J et al.(2023) substudy2 | 1.79 (1.06, 3.03） | *I^2^* = 79%（*P* < 0.01） |
| **Renal Outcome** |  |  |  |
| incidence of albuminuria | Virk SA et al.(2016) substudy1/  Rosa LCGFD et al.(2019) substudy1 | 1.54 (0.90, 2.66） | *I^2^* = 11%（*P* = 0.29） |
| incidence of eGFR exacerbation | Virk SA et al.(2016) substudy2/  Rosa LCGFD et al.(2019) substudy2 | 1.60 (0.58, 4.46） | *I^2^* = 69%（*P* = 0.07） |
| composite outcomes | Suh J et al.(2023) substudy1/  Suh J et al.(2023) substudy2 | 2.65 (1.60, 4.38） | *I^2^* = 0%（*P* = 0.82） |
| **Sample Size** |  |  |  |
| <1000 | Suh J et al.(2023) substudy1/  Suh J et al.(2023) substudy2/  Rosa LCGFD et al.(2019) substudy1/  Rosa LCGFD et al.(2019) substudy2 | 2.29 (1.45, 3.62） | *I^2^* = 11%（*P* = 0.34） |
| ≥1000 | Virk SA et al.(2016) substudy1/  Virk SA et al.(2016) substudy2 | 1.30 (0.82, 2.07） | *I^2^* = 68%（*P* = 0.08） |
| **Area** |  |  |  |
| Non-Asia | Rosa LCGFD et al.(2019) substudy1/  Rosa LCGFD et al.(2019) substudy2/  Virk SA et al.(2016) substudy1/  Virk SA et al.(2016) substudy2 | 1.38 (0.90, 2.10） | *I^2^* = 53%（*P* = 0.10） |
| Asia | Suh J et al.(2023) substudy1/  Suh J et al.(2023) substudy2 | 2.65 (1.60, 4.38） | *I^2^* = 0%（*P* = 0.82） |
| **Design** |  |  |  |
| prospective | Virk SA et al.(2016) substudy1/  Virk SA et al.(2016) substudy2 | 1.30 (0.82, 2.07） | *I^2^* = 68%（*P* = 0.08） |
| retrospective | Suh J et al.(2023) substudy1/  Suh J et al.(2023) substudy2/  Rosa LCGFD et al.(2019) substudy1/  Rosa LCGFD et al.(2019) substudy2 | 2.29 (1.45, 3.62） | *I^2^* = 11%（*P* = 0.34） |
| **Time** |  |  |  |
| median of 8 years | Rosa LCGFD et al.(2019) substudy1/  Rosa LCGFD et al.(2019) substudy2 | 1.71 (0.51, 5.78） | *I^2^* = 57%（*P* = 0.13） |
| ≧median of 8 years | Virk SA et al.(2016) substudy1/  Virk SA et al.(2016) substudy2/  Suh J et al.(2023) substudy1/  Suh J et al.(2023) substudy2 | 1.79 (1.06, 3.03） | *I^2^* =79%（*P* <0.01） |
| ***CV-HR*** | | | |
| **Index** | | | |
| Adjusted CV | / | / | */* |
| CV | / | / | */* |
| **Renal Outcome** | | | |
| incidence of albuminuria | Romero-Aroca P et al.(2021)/  Nazim J et al.(2014)/ | 1.04 (1.00, 1.08） | *I^2^* = 0%（*P* = 0.97） |
| incidence of eGFR exacerbation | Muthukumar A et al.(2024) substudy1/  Muthukumar A et al.(2024) substudy2 | 2.15 (1.51, 3.07） | *I^2^* = 0%（*P* = 0.43） |
| composite outcomes | Bille N et al.(2021) substudy1/  Bille N et al.(2021) substudy2/  Bille N et al.(2021) substudy3 | 0.58 (0.40, 0.84） | *I^2^* = 0%（*P* = 0.95） |
| **Sample Size** | | | |
| <1000 | Romero-Aroca P et al.(2021)/  Nazim J et al.(2014)/  Bille N et al.(2021) substudy1/  Bille N et al.(2021) substudy2/  Bille N et al.(2021) substudy3 | 0.78 (0.55, 1.10） | *I^2^* = 58%（*P=* 0.05） |
| ≥1000 | Muthukumar A et al.(2024) substudy1/  Muthukumar A et al.(2024) substudy2 | 2.15 (1.51, 3.07） | *I^2^* = 0%（*P* = 0.43） |
| **Area** |  |  |  |
| Non-Asia | Romero-Aroca P et al.(2021)/  Nazim J et al.(2014)/  Muthukumar A et al.(2024) substudy1/  Muthukumar A et al.(2024) substudy2 | 1.48 (0.91, 2.41） | *I^2^* = 82%（*P* <0.01） |
| Asia | Bille N et al.(2021) substudy1/  Bille N et al.(2021) substudy2/  Bille N et al.(2021) substudy3 | 0.58 (0.40, 0.84） | *I^2^* = 0%（*P* = 0.95） |
| **Design** | | | |
| prospective | Romero-Aroca P et al.(2021)/  Nazim J et al.(2014)/  Bille N et al.(2021) substudy1/  Bille N et al.(2021) substudy2/  Bille N et al.(2021) substudy3 | 0.78 (0.55, 1.10） | *I^2^* = 58%（*P=* 0.05） |
| retrospective | Muthukumar A et al.(2024) substudy1/  Muthukumar A et al.(2024) substudy2 | 2.15 (1.51, 3.07） | *I^2^* = 0%（*P* = 0.43） |
| **Time** | | | |
| median of 8 years | Romero-Aroca P et al.(2021)/  Bille N et al.(2021) substudy1/  Bille N et al.(2021) substudy2/  Bille N et al.(2021) substudy3 | 0.64 (0.46, 0.90） | *I^2^* = 0%（*P* = 0.63） |
| ≧median of 8 years | Nazim J et al.(2014)/  Muthukumar A et al.(2024) substudy1/  Muthukumar A et al.(2024) substudy2 | 1.62 (0.89, 2.94） | *I^2^* =88%（*P* <0.01） |
| **Quartile** | | | |
| Q2/Q1 | Muthukumar A et al.(2024) substudy1/  Bille N et al.(2021) substudy1 | 1.10 (0.38, 3.15） | *I^2^* = 85%（*P* = 0.01） |
| Q3/Q1 | Muthukumar A et al.(2024) substudy2/  Bille N et al.(2021) substudy2 | 1.19 (0.28, 5.09） | *I^2^* =92%（*P*＜0.01） |
| Q4/Q1 | Bille N et al.(2021) substudy3 | 0.55 (0.29, 1.04） | */* |
| Q5/Q1 | / | / | */* |
| **CV-OR** |  |  |  |
| **Index** |  |  |  |
| Adjusted CV | / | / | */* |
| CV | / | / | */* |
| **Renal Outcome** |  |  |  |
| incidence of albuminuria | Virk SA et al.(2016) substudy1/  Rosa LCGFD et al.(2019) substudy2 | 2.44 (0.86, 6.90） | *I^2^* = 56%（*P* = 0.13） |
| incidence of eGFR exacerbation | Virk SA et al.(2016) substudy2/  Rosa LCGFD et al.(2019) substudy1 | 1.08 (1.02, 1.14） | *I^2^* = 0%（*P* = 0.32） |
| composite outcomes | / | / | */* |
| **Sample Size** |  |  |  |
| ＜1000 | Rosa LCGFD et al.(2019) substudy1/  Rosa LCGFD et al.(2019) substudy2 | 1.70 (0.21, 14.12） | *I^2^* = 82%（*P* = 0.02） |
| ≥1000 | Virk SA et al.(2016) substudy1/  Virk SA et al.(2016) substudy2 | 1.25 (0.83, 1.88） | *I^2^* = 67%（*P* =0.08） |
| **Area** |  |  |  |
| South America | Rosa LCGFD et al.(2019) substudy1/  Rosa LCGFD et al.(2019) substudy2 | 1.70 (0.21, 14.12） | *I^2^* = 82%（*P* = 0.02） |
| North America | Virk SA et al.(2016) substudy1/  Virk SA et al.(2016) substudy2 | 1.25 (0.83, 1.88） | *I^2^* = 67%（*P* =0.08） |
| **Design** |  |  |  |
| prospective | Virk SA et al.(2016) substudy1/  Virk SA et al.(2016) substudy2 | 1.25 (0.83, 1.88） | *I^2^* = 67%（*P* =0.08） |
| retrospective | Rosa LCGFD et al.(2019) substudy1/  Rosa LCGFD et al.(2019) substudy2 | 1.70 (0.21, 14.12） | *I^2^* = 82%（*P* = 0.02） |
| **Time** |  |  |  |
| median of 8 years | Rosa LCGFD et al.(2019) substudy1/  Rosa LCGFD et al.(2019) substudy2 | 1.70 (0.21, 14.12） | *I^2^* = 82%（*P* = 0.02） |
| ≧median of 8 years | Virk SA et al.(2016) substudy1/  Virk SA et al.(2016) substudy2 | 1.25 (0.83, 1.88） | *I^2^* = 67%（*P* =0.08） |
| **Quartile** |  |  |  |
| Q2/Q1 | / | / | */* |
| Q3/Q1 | / | / | */* |
| Q4/Q1 | / | / | */* |
| Q5/Q1 | / | / | */* |

SD: standard deviation; CV: coefficient of variation; HR: hazard ratio; RR: relative ratio; OR: Odds Ratio.

**Table 3**  Subgroup analyses for SD, CV, HGI and HVS of renal outcomes in patients with T2DM

| Subgroup | Studies | Pooled HR/OR (95% CI) | *I^2^* (*p* value) |
| --- | --- | --- | --- |
| ***SD-HR*** | | | |
| **Index** | | | |
| Adjusted SD | Ma C et al.(2022) /  Luk AO et al.(2013) /  Hsu CC et al.(2012) | 1.32 (1.15, 1.52） | *I^2^* = 67%（*P* < 0.01） |
| SD | Yan Y et al.(2022)/  Teh XR et al.(2025)/  Shen ZZ et al.(2017)/  Lee MY et al.(2018)/  Cardoso CRL et al.(2018) | 1.23 (1.09, 1.38） | *I^2^* = 57%（*P* < 0.01） |
| **Renal Outcome** | | | |
| incidence of albuminuria | Yan Y et al.(2022) substudy1/  Yan Y et al.(2022) substudy2/  Yan Y et al.(2022) substudy3/  Shen ZZ et al.(2017)/  Hsu CC et al.(2012)/ | 1.33 (1.18, 1.50） | *I^2^* = 8%（*P* = 0.37） |
| incidence of eGFR exacerbation | Yan Y et al.(2022) substudy4/  Yan Y et al.(2022) substudy5/  Yan Y et al.(2022) substudy6 | 1.20 (1.09, 1.31） | *I^2^* = 0%（*P* = 0.61） |
| composite outcomes | Teh XR et al.(2025)/  Lee MY et al.(2018) substudy1/  Lee MY et al.(2018) substudy2/  Ma C et al.(2022) substudy1/  Ma C et al.(2022) substudy2/  Ma C et al.(2022) substudy3/  Luk AO et al.(2013) substudy1/  Luk AO et al.(2013) substudy2/  Cardoso CRL et al.(2018) substudy1/  Cardoso CRL et al.(2018) substudy2 | 1.21 (1.06, 1.38） | *I^2^* = 79%（*P* < 0.01） |
| **Sample Size** | | | |
| <1000 | Shen ZZ et al.(2017)/  Lee MY et al.(2018)/  Hsu CC et al.(2012)/  Cardoso CRL et al.(2018) | 1.17 (1.00, 1.38） | *I^2^* = 56%（*P* = 0.01） |
| ≥1000 | Yan Y et al.(2022)/  Teh XR et al.(2025)/  Ma C et al.(2022)/  Luk AO et al.(2013) | 1.31 (1.18, 1.45） | *I^2^* = 67%（*P* < 0.01） |
| **Area** | | | |
| Non-Asia | Cardoso CRL et al.(2018) substudy1/  Cardoso CRL et al.(2018) substudy2 | 1.26 (1.08, 1.47） | *I^2^* = 0%（*P* = 0.92） |
| Asia | Yan Y et al.(2022)/  Teh XR et al.(2025)/  Shen ZZ et al.(2017)/  Lee MY et al.(2018)/  Ma C et al.(2022)/  Luk AO et al.(2013)/  Hsu CC et al.(2012) | 1.27 (1.16, 1.39） | *I^2^* = 64%（*P* < 0.01） |
| **Design** |  |  |  |
| prospective | Lee MY et al.(2018)/  Ma C et al.(2022)/  Luk AO et al.(2013)/  Hsu CC et al.(2012)/  Cardoso CRL et al.(2018) | 1.26 (1.10, 1.44） | *I^2^* = 70%（*P* < 0.01） |
| retrospective | Yan Y et al.(2022)/  Teh XR et al.(2025)/  Shen ZZ et al.(2017)/ | 1.23 (1.13, 1.35） | *I^2^* = 23%（*P* = 0.25） |
| **Time** | | | |
| <median of 8 years | Teh XR et al.(2025)/  Shen ZZ et al.(2017)/  Lee MY et al.(2018)/  Luk AO et al.(2013)/  Hsu CC et al.(2012)/ | 1.21 (1.08, 1.36） | *I^2^* = 72%（*P* < 0.01） |
| ≧median of 8 years | Cardoso CRL et al.(2018)/  Yan Y et al.(2022) | 1.26 (1.15, 1.38） | *I^2^* =0%（*P* =0.47） |
| **Quartile** | | | |
| Q2/Q1 | Yan Y et al.(2022) substudy1/  Yan Y et al.(2022) substudy5/  Ma C et al.(2022) substudy1/  Hsu CC et al.(2012) substudy1/  Hsu CC et al.(2012) substudy4 | 1.13 (0.92, 1.39） | *I^2^* = 35%（*P* = 0.18） |
| Q3/Q1 | Yan Y et al.(2022) substudy2/  Yan Y et al.(2022) substudy6/  Ma C et al.(2022) substudy2/  Hsu CC et al.(2012) substudy2/  Hsu CC et al.(2012) substudy5 | 1.16 (0.99, 1.35） | *I^2^* =1%（*P* = 0.40） |
| Q4/Q1 | Yan Y et al.(2022) substudy3/  Ma C et al.(2022) substudy3/  Hsu CC et al.(2012) substudy3/  Hsu CC et al.(2012) substudy6 | 1.52 (1.24, 1.86） | *I^2^* =19%（*P* = 0.30） |
| ***SD-OR*** |  |  |  |
| **Index** |  |  |  |
| Adjusted SD | / | / | */* |
| SD | / | / | / |
| **Renal Outcome** |  |  |  |
| incidence of albuminuria | Lee Set al.(2021) substudy1/  Lee Set al.(2021) substudy2/  Lee Set al.(2021) substudy3 | 1.11 (1.03, 1.20） | *I^2^* = 0%（*P* = 0.42） |
| incidence of eGFR exacerbation | Song KH et al.(2019) substudy1/  Song KH et al.(2019) substudy2 | 5.44 (2.49, 11.86） | *I^2^* = 0%（*P* = 0.85） |
| composite outcomes | Teliti M et al.(2018) substudy1/  Teliti M et al.(2018) substudy2 | 1.63 (1.19, 2.24） | *I^2^* = 0%（*P* = 0.95） |
| **Sample Size** |  |  |  |
| <1000 | Song KH et al.(2019) substudy1/  Song KH et al.(2019) substudy2/  Teliti M et al.(2018) substudy1/  Teliti M et al.(2018) substudy2 | 2.39 (1.38, 4.13） | *I^2^* = 62%（*P* = 0.05） |
| ≥1000 | Lee Set al.(2021) substudy1/  Lee Set al.(2021) substudy2/  Lee Set al.(2021) substudy3 | 1.11 (1.03, 1.20） | *I^2^* = 0%（*P* = 0.42） |
| **Area** |  |  |  |
| Non-Asia | Teliti M et al.(2018) substudy1/  Teliti M et al.(2018) substudy2 | 1.63 (1.19, 2.24） | *I^2^* = 0%（*P* = 0.95） |
| Asia | Lee Set al.(2021) substudy1/  Lee Set al.(2021) substudy2/  Lee Set al.(2021) substudy3/  Song KH et al.(2019) substudy1/  Song KH et al.(2019) substudy2 | 1.23 (0.99, 1.53） | *I^2^* = 77%（*P* < 0.01） |
| **Design** |  |  |  |
| prospective | Teliti M et al.(2018) substudy1/  Teliti M et al.(2018) substudy2 | 1.63 (1.19, 2.24） | *I^2^* = 0%（*P* = 0.95） |
| retrospective | Lee Set al.(2021) substudy1/  Lee Set al.(2021) substudy2/  Lee Set al.(2021) substudy3/  Song KH et al.(2019) substudy1/  Song KH et al.(2019) substudy2 | 1.23 (0.99, 1.53） | *I^2^* = 77%（*P* < 0.01） |
| **Time** |  |  |  |
| <median of 8 years | Song KH et al.(2019) substudy1/  Song KH et al.(2019) substudy2 | 5.44 (2.49, 11.86） | *I^2^* = 0%（*P* = 0.85） |
| ≧median of 8 years | Lee Set al.(2021) substudy1/  Lee Set al.(2021) substudy2/  Lee Set al.(2021) substudy3 | 1.11 (1.03, 1.20） | *I^2^* = 0%（*P* = 0.42） |
| **Quartile** |  |  |  |
| Q2/Q1 | / | / | */* |
| Q3/Q1 | / | / | */* |
| Q4/Q1 | / | / | */* |
| ***HVS-HR*** | | | |
| **Renal Outcome** | | | |
| incidence of albuminuria | Yan Y et al.(2022) substudy1/  Yan Y et al.(2022) substudy2/  Yan Y et al.(2022) substudy3 | 1.43 (1.14, 1.81） | *I^2^* = 0%（*P* = 0.72） |
| incidence of eGFR exacerbation | Yan Y et al.(2022) substudy4/  Yan Y et al.(2022) substudy5/  Yan Y et al.(2022) substudy6 | 1.08 (0.95, 1.22） | *I^2^* = 0%（*P =* 0.56） |
| composite outcomes | / | / | */* |
| **Sample Size** | | | |
| <1000 | / | / | */* |
| ≥1000 | / | / | */* |
| **Area** | | | |
| Non-Asia | / | / | */* |
| Asia | / | / | */* |
| **Time** | | | |
| <median of 8 years | / | / | */* |
| ≧median of 8 years | / | / | */* |
| **Quartile** | | | |
| Q2/Q1 | Yan Y et al.(2022) substudy1/  Yan Y et al.(2022) substudy4 | 1.07 (0.92, 1.23） | *I^2^* = 14%（*P* = 0.28） |
| Q3/Q1 | Yan Y et al.(2022) substudy2/  Yan Y et al.(2022) substudy5 | 1.24 (1.03, 1.49） | *I^2^* =13%（*P* = 0.28） |
| Q4/Q1 | Yan Y et al.(2022) substudy3/  Yan Y et al.(2022) substudy6 | 1.32 (0.93, 1.87） | *I^2^* =43%（*P =* 0.19） |
| Q5/Q1 | / | / | */* |
| ***HVS-OR*** |  |  |  |
| **Renal Outcome** |  |  |  |
| incidence of albuminuria | Lee Set al.(2021) substudy2 | 1.00 (0.50, 2.00） | *I^2^* = 0%（*P* = 0.64） |
| incidence of eGFR exacerbation | Zhou Y et al.(2022) substudy1/  Zhou Y et al.(2022) substudy2/  Zhou Y et al.(2022) substudy3/ | 1.08 (1.04, 1.11） | *I^2^* = 40%（*P=*0.19） |
| composite outcomes | Lee Set al.(2021) substudy1/  Lee Set al.(2021) substudy3 | 1.00 (1.00, 1.01） | *I^2^* = 61%（*P=* 0.11） |
| **Sample Size** |  |  |  |
| ＜1000 | / | / | */* |
| ≥1000 | / | / | */* |
| **Area** |  |  |  |
| China | Zhou Y et al.(2022) substudy1/  Zhou Y et al.(2022) substudy2/  Zhou Y et al.(2022) substudy3 | 1.08 (1.04, 1.11） | *I^2^* = 40%（*P=*0.19） |
| HongKong, China | Lee Set al.(2021) substudy1/  Lee Set al.(2021) substudy2/  Lee Set al.(2021) substudy3 | 1.00(1.00, 1.01） | *I^2^* = 22%（*P* = 0.28） |
| **Time** |  |  |  |
| <median of 8 years | Zhou Y et al.(2022) substudy1/  Zhou Y et al.(2022) substudy2/  Zhou Y et al.(2022) substudy3 | 1.08 (1.04, 1.11） | *I^2^* = 40%（*P=*0.19） |
| ≧median of 8 years | Lee Set al.(2021) substudy1/  Lee Set al.(2021) substudy2/  Lee Set al.(2021) substudy3 | 1.00(1.00, 1.01） | *I^2^* = 22%（*P* = 0.28） |
| **Quartile** |  |  |  |
| Q2/Q1 | / | / | */* |
| Q3/Q1 | / | / | */* |
| Q4/Q1 | / | / | */* |
| Q5/Q1 | / | / | */* |
| ***CV-HR*** | | | |
| **Index** | | | |
| Adjusted CV | / | / | */* |
| CV | / | / | */* |
| **Renal Outcome** | | | |
| incidence of albuminuria | Yan Y et al.(2022)substudy1/  Yan Y et al.(2022)substudy2/  Yan Y et al.(2022)substudy3 | 1.43 (1.14, 1.79） | *I^2^* = 0%（*P* = 0.56） |
| incidence of eGFR exacerbation | Yang YF et al.(2015)/  Yan Y et al.(2022)substudy4/  Yan Y et al.(2022)substudy5/  Yan Y et al.(2022)substudy6 | 1.17 (1.09, 1.25） | *I^2^* = 0%（*P* = 0.70） |
| composite outcomes | Teh XR et al.(2025)/  Slieker RC et al.(2019)/  Ma C et al.(2022)/  Sun B et al.(2022) | 1.39 (1.07, 1.80） | *I^2^* = 75%（*P* < 0.11） |
| **Sample Size** | | | |
| <1000 | Yan Y et al.(2022) | 1.21 (1.09, 1.33） | *I^2^* = 0%（*P* = 0.46） |
| ≥1000 | Ma C et al.(2022)/  Sun B et al.(2022)/  Slieker RC et al.(2019)/  Yang YF et al.(2015)/  Teh XR et al.(2025) | 1.21 (1.06, 1.39） | *I^2^* = 67%（*P*＜0.01） |
| **Area** |  |  |  |
| Asia | Yang YF et al.(2015)/  Yan Y et al.(2022)/  Teh XR et al.(2025)/  Ma C et al.(2022)/  Sun B et al.(2022) | 1.29 (1.16, 1.43） | *I^2^* = 54%（*P<*0.01） |
| Non-Asia | Slieker RC et al.(2019) | 0.99 (0.88, 1.12） | *I^2^* = 0%（*P* = 0.57） |
| **Design** | | | |
| prospective | Yang YF et al.(2015)/  Slieker RC et al.(2019)/  Ma C et al.(2022) | 1.14 (1.03, 1.26） | *I^2^* = 48%（*P* =0.04） |
| retrospective | Yan Y et al.(2022)/  Teh XR et al.(2025)/  Sun B et al.(2022) | 1.38 (1.13, 1.69） | *I^2^* = 64%（*P* < 0.01） |
| **Time** | | | |
| <median of 8 years | Teh XR et al.(2025)/  Sun B et al.(2022) | 3.92 (2.16, 7.14） | *I^2^* = 0%（*P=*0.47） |
| ≧median of 8 years | Yang YF et al.(2015)/  Yan Y et al.(2022) | 1.19 (1.11, 1.27） | *I^2^* = 0%（*P* = 0.55） |
| **Quartile** | | | |
| Q2/Q1 | Yang YF et al.(2015) substudy1/  Yan Y et al.(2022) substudy1/  Yan Y et al.(2022) substudy4/  Slieker RC et al.(2019) substudy1/  Ma C et al.(2022) substudy1 | 1.17 (1.06, 1.28） | *I^2^* = 0%（*P* = 0.41） |
| Q3/Q1 | Yang YF et al.(2015) substudy2/  Yan Y et al.(2022) substudy2/  Yan Y et al.(2022) substudy5/  Slieker RC et al.(2019) substudy2/  Ma C et al.(2022) substudy2 | 1.11 (0.93, 1.32） | *I^2^* =52%（*P=* 0.08） |
| Q4/Q1 | Yang YF et al.(2015) substudy3/  Yan Y et al.(2022) substudy3/  Yan Y et al.(2022) substudy6/  Slieker RC et al.(2019) substudy3/  Ma C et al.(2022) substudy3 | 1.23 (1.00, 1.51） | *I^2^* =55%（*P* = 0.06） |
| Q5/Q1 | Yang YF et al.(2015) substudy4/  Slieker RC et al.(2019) substudy4 | 1.19 (0.98, 1.45） | *I^2^* =45%（*P* = 0.18） |
| ***CV-OR*** |  |  |  |
| **Index** |  |  |  |
| Adjusted CV | / | / | */* |
| CV | / | / | */* |
| **Renal Outcome** |  |  |  |
| incidence of albuminuria | Low S et al.(2016) substudy3/  Lee S et al.(2021) substudy2 | 1.27 (0.71, 2.29） | *I^2^* = 81%（*P* = 0.02） |
| incidence of eGFR exacerbation | Low S et al.(2016) substudy1/  Low S et al.(2016) substudy2/  Low S et al.(2016) substudy4/  Low S et al.(2016) substudy5/  Low S et al.(2017) | 1.73 (1.36, 2.19） | *I^2^* = 56%（*P* = 0.02） |
| composite outcomes | Lee S et al.(2021) substudy1/  Lee S et al.(2021) substudy3/  Ma Y et al.(2023) | 1.02 (0.98, 1.06） | *I^2^* = 87%（*P* < 0.01） |
| **Sample Size** |  |  |  |
| <1000 | Ma Y et al.(2023)/  Low S et al.(2016) | 1.97 (1.63, 2.38） | *I^2^* = 0%（*P* = 0.42） |
| ≥1000 | Lee S et al.(2021)/  Low S et al.(2017) | 1.02 (0.98, 1.05） | *I^2^* = 80%（*P*＜0.01） |
| **Area** |  |  |  |
| Asia | Ma Y et al.(2023)/  Lee S et al.(2021) substudy1/  Lee S et al.(2021) substudy2/  Lee S et al.(2021) substudy3 | 1.01 (0.98, 1.03） | *I^2^* = 85%（*P<0*.01） |
| Non-Asia | Low S et al.(2016)/  Low S et al.(2017) | 1.74 (1.40, 2.16） | *I^2^* = 51%（*P* = 0.03） |
| **Design** |  |  |  |
| prospective | Low S et al.(2016) | 1.86 (1.52, 2.28） | *I^2^* = 0%（*P* =0.57） |
| retrospective | Ma Y et al.(2023)/  Low S et al.(2017)/  Lee S et al.(2021) | 1.03 (0.99, 1.07） | *I^2^* = 84%（*P* <0.01） |
| **Time** |  |  |  |
| <median of 8 years | Low S et al.(2016)/  Ma Y et al.(2023)/  Low S et al.(2017) | 1.81 (1.47, 2.23） | *I^2^* = 52%（*P=*0.02） |
| ≧median of 8 years | Lee S et al.(2021) | 1.00 (0.99, 1.02） | *I^2^* = 46%（*P* = 0.16） |
| **Quartile** |  |  |  |
| Q2/Q1 | Low S et al.(2016) substudy1/  Low S et al.(2017) substudy1/  Low S et al.(2017) substudy4 | 1.27 (0.83, 1.95） | *I^2^* = 53%（*P* = 0.12） |
| Q3/Q1 | Low S et al.(2016) substudy2/  Low S et al.(2017) substudy2/  Low S et al.(2017) substudy5 | 1.86 (1.04, 3.35） | *I^2^* =75%（*P* =0.02） |
| Q4/Q1 | Low S et al.(2017) substudy3/  Low S et al.(2017) substudy6 | 2.26 (1.52, 3.36） | *I^2^* =19%（*P* = 0.27） |
| Q5/Q1 | / | / | */* |
| ***HGI-HR*** |  |  |  |
| **Renal Outcome** |  |  |  |
| incidence of albuminuria | Lin CH et al.(2022) substudy5 | 1.02 (0.81, 1.28） | */* |
| incidence of eGFR exacerbation | Lin CH et al.(2022) substudy1/  Lin CH et al.(2022) substudy2/  Lin CH et al.(2022) substudy3/  Lin CH et al.(2022) substudy4 | 1.99 (1.57, 2.50） | *I^2^* = 0%（*P* = 0.94） |
| composite outcomes | Cardoso CRL et al.(2024) substudy1/  Cardoso CRL et al.(2024) substudy2/  Cardoso CRL et al.(2024) substudy3/  Cardoso CRL et al.(2024) substudy4 | 1.26 (1.14, 1.40） | *I^2^* = 0%（*P* = 0.44） |
| **Sample Size** |  |  |  |
| <1000 | / | / | */* |
| ≥1000 | / | / | */* |
| **Design** |  |  |  |
| prospective | Cardoso CRL et al.(2024) substudy1/  Cardoso CRL et al.(2024) substudy2/  Cardoso CRL et al.(2024) substudy3/  Cardoso CRL et al.(2024) substudy4 | 1.26 (1.14, 1.40） | *I^2^* = 0%（*P* = 0.44） |
| retrospective | Lin CH et al.(2022) substudy1/  Lin CH et al.(2022) substudy2/  Lin CH et al.(2022) substudy3/  Lin CH et al.(2022) substudy4/  Lin CH et al.(2022) substudy5 | 1.71 (1.17, 2.49） | *I^2^* = 76%（*P* < 0.01） |
| **Time** |  |  |  |
| <median of 8 years | Lin CH et al.(2022) substudy1/  Lin CH et al.(2022) substudy2/  Lin CH et al.(2022) substudy3/  Lin CH et al.(2022) substudy4/  Lin CH et al.(2022) substudy5 | 1.71 (1.17, 2.49） | *I^2^* = 76%（*P* < 0.01） |
| ≧median of 8 years | Cardoso CRL et al.(2024) substudy1/  Cardoso CRL et al.(2024) substudy2/  Cardoso CRL et al.(2024) substudy3/  Cardoso CRL et al.(2024) substudy4 | 1.26 (1.14, 1.40） | *I^2^* = 0%（*P* = 0.44） |
| **Quartile** |  |  |  |
| Q2/Q1 | / | / | */* |
| Q3/Q1 | / | / | */* |
| Q4/Q1 | / | / | */* |
| Q5/Q1 | / | / | */* |
| ***Per 1% increase in SD-HR*** | | | |
| **Index** |  |  |  |
| Adjusted SD | / | / | */* |
| SD | / | / | */* |
| **Renal Outcome** |  |  |  |
| incidence of albuminuria | Sugawara A et al.(2012) substudy1/  Sugawara A et al.(2012) substudy2 | 1.24 (1.09, 1.41） | *I^2^* = 0%（*P* = 0.43） |
| incidence of eGFR exacerbation | Rodríguez-Segade S et al.(2012)/  Wu TE et al.(2022) substudy1/  Wu TE et al.(2022) substudy2 | 1.55 (1.26, 1.91） | *I^2^* = 53%（*P* = 0.12） |
| composite outcomes | / | / | */* |
| **Sample Size** |  |  |  |
| <1000 | Sugawara A et al.(2012) substudy1/  Sugawara A et al.(2012) substudy2 | 1.24 (1.09, 1.41） | *I^2^* = 0%（*P* = 0.43） |
| ≥1000 | Rodríguez-Segade S et al.(2012)/  Wu TE et al.(2022) substudy1/  Wu TE et al.(2022) substudy2 | 1.55 (1.26, 1.91） | *I^2^* = 53%（*P* = 0.12） |
| **Area** |  |  |  |
| Asia | Sugawara A et al.(2012) substudy1/  Sugawara A et al.(2012) substudy2/  Wu TE et al.(2022) substudy1/  Wu TE et al.(2022) substudy2 | 1.44 (1.18, 1.75） | *I^2^* = 66%（*P=*0.03） |
| Non-Asia | Rodríguez-Segade S et al.(2012) | 1.37 (1.12, 1.68） | */* |
| **Design** |  |  |  |
| prospective | / | / | */* |
| retrospective | / | / | */* |
| **Time** |  |  |  |
| <median of 8 years | Sugawara A et al.(2012) substudy1/  Sugawara A et al.(2012) substudy2/  Rodríguez-Segade S et al.(2012) | 1.28 (1.14, 1.42） | *I^2^* = 0%（*P* = 0.52） |
| ≧median of 8 years | Wu TE et al.(2022) substudy1/  Wu TE et al.(2022) substudy2 | 1.72 (1.21, 2.45） | *I^2^* = 64%（*P* = 0.10） |
| **Quartile** |  |  |  |
| Q2/Q1 | / | / | */* |
| Q3/Q1 | / | / | */* |
| Q4/Q1 | / | / | */* |
| Q5/Q1 | / | / | */* |

SD: standard deviation; CV: coefficient of variation; HVS: HbA1c variability score; HGI: Hemoglobin glycation index; HR: hazard ratio; RR: relative ratio; OR: Odds Ratio.

**Table 4.** Sensitivity Analysis of the Association Between HbA1c Variability and Mortality in Patients in DM.

| Type analysis | Trial | Pooled HR/OR(95% CI) | *I^2^* (*p* value) |
| --- | --- | --- | --- |
| ***CV-TDM*** | | | |
| Excluding trials | Afghahi H et al.(2022) substudy5 | 1.36（1.21-1.53） | *I^2^* = 0%（*P* =0.54） |
| ***CV-T2DM*** | | | |
| Excluding trials | Yang YF et al.(2015) substudy4 | / | *I^2^* = 55%（*P=*0.02） |

CV: coefficient of variation; HR: hazard ratio; OR: Odds Ratio.

**Table 5.** Sensitivity Analysis of the Association Between HbA1c Variability and Renal Progression in Patients with T1DM

| Type analysis | Trial | Pooled HR/OR (95% CI) | *I^2^* (*p* value) |
| --- | --- | --- | --- |
| ***SD-HR*** | | | |
| Excluding trials with low quality | / | / | / |
| Excluding trials without specified follow-up | Bille N et al.(2021) | 1.95 (1.41, 2.71） | *I^2^* = 64%（*P* =0.04） |
| Excluding trials which reported RRs | / | / | / |
| Fixed model analysis | Wadén J et al.(2009)/  Romero-Aroca P et al.(2021) | 0.89 (0.56, 1.41)/  0.92 (0.56, 1.53) | *I^2^* = 83%（*P* <0.01)/  *I^2^* = 87%（*P* <0.01） |
| ***SD-OR*** | | | |
| Excluding trials with SD | Virk SA et al.(2016) | 2.29 (1.45, 3.62） | *I^2^* = 11%（*P=*0.34） |
| Excluding trials with follow-up ＜5 years | Rosa LCGFD et al.(2019) | 1.79 (1.06, 3.03） | *I^2^* = 79%（*P*＜0.01） |
| Excluding trials which reported RRs | / | / | */* |
| Fixed model analysis | Suh J et al.(2023) | 1.38 (0.90, 2.10） | *I^2^* = 53%（*P=* 0.10） |
| ***HVS-HR*** |  |  |  |
| Excluding trials | Bille N et al.(2021) substudy2/  Bille N et al.(2021) substudy4 | 0.69 (0.46, 1.04)/  0.49 (0.32, 0.75) | *I^2^* = 0%（*P=* 0.41)/  *I^2^* = 0%（*P=* 0.60) |
| Excluding trials with follow-up ＜5 years | / | / | */* |
| Excluding trials which reported RRs | / | / | */* |
| Fixed model analysis | / | / | */* |
| ***CV-HR*** |  |  |  |
| Excluding trials | Muthukumar A et al.(2024) | 0.78 (0.55, 1.10） | *I^2^* = 58%（*P=* 0.05） |
| Excluding trials with follow-up ＜5 years | Bille N et al.(2021) | 1.48 (0.91, 2.41） | *I^2^* = 82%（*P<0.01*） |
| Excluding trials which reported RRs | / | / | / |
| Fixed model analysis | Romero-Aroca P et al.(2021)/  Nazim J et al.(2014) | 1.03 (0.69, 1.54)/  1.01 (0.57, 1.77） | *I^2^* = 81%（*P<0.01)/*  *I^2^* = 81%（*P<0.01)* |
| ***CV-OR*** | | | |
| Excluding trials with | Virk SA et al.(2016) substudy1/  Virk SA et al.(2016) substudy2 | 1.70 (0.21, 14.12)/  1.64 (0.63, 4.24) | *I^2^* = 66%（*P*＝0.05)/  *I^2^* = 64%（*P*＝0.06) |
| Excluding trials with follow-up ＜5 years | Rosa LCGFD et al.(2019) substudy1/  Rosa LCGFD et al.(2019) substudy2 | 1.55 (0.87, 2.76)/  1.17 (0.81, 1.68) | *I^2^* = 75%（*P*＝0.02）/  *I^2^* = 50%（*P*＝0.13） |
| Excluding trials which reported RRs | / | / | */* |
| Fixed model analysis | / | / | */* |
| ***Per 1% increase in SD*** | | | |
| Excluding trials with low quality | / | / | / |
| Excluding trials with follow-up ＜5 years | / | / | */* |
| Excluding trials which reported RRs | / | / | */* |
| Fixed model analysis | Raman S et al.(2016) substudy1/  Raman S et al.(2016) substudy2 | 1.48 (1.26, 1.74)/  1.29 (1.10, 1.52) | *I^2^* = 28%（*P* =0.24)/  *I^2^* = 0%（*P* =0.89) |

SD: standard deviation; CV: coefficient of variation; HVS: HbA1c variability score; ; HR: hazard ratio; RR: relative ratio; OR: Odds Ratio..

**Table 6.** Sensitivity Analysis of the Association Between HbA1c Variability and Renal Progression in Patients with T2DM

| Type analysis | Trial | Pooled HR/OR (95% CI) | *I^2^* (*p* value) |
| --- | --- | --- | --- |
| ***SD-HR*** | | | |
| Excluding trials with low quality | / | / | / |
| Excluding trials with follow-up ＜5 years | Lee MY et al.(2018)/  Teh XR et al.(2025) | 1.48 (1.25-1.76)/  1.28 (1.17, 1.41) | *I^2^* = 49%（*P*<0.01)/  *I^2^* = 61%（*P*<0.01) |
| Excluding trials which reported RRs | / | / | / |
| Fixed model analysis | Hsu CC et al.(2012) | 1.29 (1.17, 1.42） | *I^2^* = 68%（*P* < 0.01） |
| ***SD-OR*** |  |  |  |
| Excluding trials with low quality | / | / | / |
| Excluding trials with follow-up ＜5 years | Song KH et al.(2019) | 1.16 (1.03, 1.30） | *I^2^* = 43%（*P=*0.13） |
| Excluding trials which reported RRs | / | / | / |
| Fixed model analysis | Teliti M et al.(2018)/  Song KH et al.(2019) | 1.32 (1.08, 1.60)/  1.16 (1.03, 1.30） | *I^2^* = 73%（*P* <0.01）/*I^2^* = 43%（*P=*0.13） |
| ***CV-HR*** | | | |
| Excluding trials with low quality | / | / | / |
| Excluding trials with follow-up ＜5 years | Teh XR et al.(2025)/  Sun B et al.(2022) | 1.17 (1.08, 1.27)/  1.19 (1.09, 1.30) | *I^2^* = 43%（*P* = 0.03）/*I^2^* = 53%（*P*＜0.01） |
| Excluding trials which reported RRs | / | / | */* |
| Fixed model analysis | Teh XR et al.(2025)/  Sun B et al.(2022) | 1.17 (1.08, 1.27)/  1.19 (1.09, 1.30) | *I^2^* = 43%（*P* = 0.03）/*I^2^* = 53%（*P*＜0.01） |
| ***CV-OR*** |  |  |  |
| Excluding trials with low quality | Low S et al.(2016) | 1.81 (1.47, 2.23) | *I^2^* = 52%（*P =* 0.02） |
| Excluding trials with follow-up ＜5 years | Ma Y et al.(2023) | 1.06 (1.01, 1.10) | *I^2^* = 83%（*P <* 0.01） |
| Excluding trials which reported RRs | / | / | */* |
| Fixed model analysis | Ma Y et al.(2023) | 1.06 (1.01, 1.10) | *I^2^* = 83%（*P <* 0.01） |
| ***HVS-HR*** |  |  |  |
| Excluding trials with low quality | Yan Y et al.(2022) substudy4 | 1.26 (1.09, 1.46） | *I^2^* = 0%（*P=*0.56） |
| Excluding trials with follow-up ＜5 years | / | / | */* |
| Excluding trials which reported RRs | / | / | */* |
| Fixed model analysis | / | / | */* |
| ***HVS-OR*** |  |  |  |
| Excluding trials with low quality | / | / | / |
| Excluding trials with follow-up ＜5 years | Zhou Y et al.(2022) | 1.00 (1.00, 1.01） | *I^2^* = 22%（*P=*0.28） |
| Excluding trials which reported RRs | / | / | */* |
| Fixed model analysis | / | / | */* |
| ***HGI-HR*** |  |  |  |
| Excluding trials with low quality | / | / | / |
| Excluding trials with follow-up ＜5 years | / | / | / |
| Excluding trials which reported RRs | / | / | / |
| Fixed model analysis | Lin CH et al.(2022) substudy5 | 1.46 (1.25, 1.70） | *I^2^* = 54%（*P* =0.03） |
| ***SD-per 1% increase-HR*** | | | |
| Excluding trials | Wu TE et al.(2022) substudy2 | 1.31 (1.19, 1.45） | *I^2^* = 0%（*P* ＝ 0.44） |
| Excluding trials with follow-up ＜5 years | Sugawara A et al.(2012) | 1.55 (1.26, 1.91） | *I^2^* = 53%（*P*＝ 0.12） |
| Excluding trials which reported RRs | / | / | */* |
| Fixed model analysis | Sugawara A et al.(2012) | 1.55 (1.26, 1.91） | *I^2^* = 53%（*P*＝ 0.12） |
| ***SD-per 1% increase-OR*** | | | |
| Excluding trials with low quality | / | / | / |
| Excluding trials with follow-up ＜5 years | / | / | */* |
| Excluding trials which reported RRs | / | / | */* |
| Fixed model analysis | / | / | */* |
| ***CV-per 1% increase-HR*** | | | |
| Excluding trials with low quality | / | / | / |
| Excluding trials with follow-up ＜5 years | / | / | */* |
| Excluding trials which reported RRs | / | / | */* |
| Fixed model analysis | / | / | */* |
| ***CV-per 1% increase-OR*** | | | |
| Excluding trials with low quality | / | / | / |
| Excluding trials with follow-up ＜5 years | / | / | */* |
| Excluding trials which reported RRs | / | / | */* |
| Fixed model analysis | / | / | */* |

SD: standard deviation; CV: coefficient of variation; HVS: HbA1c variability score; HGI: Hemoglobin glycation index; HR: hazard ratio; RR: relative ratio; OR: Odds Ratio.

**Table 7: Publication bias based on Egger tests.**

| **Primary outcomes** | ***P* value** |
| --- | --- |
| Mortality Rate-T2DM-CV-HR | 0.160 |
| Incidence Rate-T2DM-SD-HR | 0.389 |
| Incidence Rate-T2DM-CV-HR | 0.009 |
| Incidence Rate-T2DM-CV-OR | <0.05 |
| Incidence Rate-T1DM-SD-HR | 0.036 |

**Table 8: Results of Trim-and-Fill Analysis**

| **Incidence Rate-T2DM-CV-HR** | **Before Trim-and-Fill** | **After Trim-and-Fill** | **Significance of Changes** |
| --- | --- | --- | --- |
| Effect Size | 0.165(0.093, 0.237) | 0.139(0.013, 0.265) | Weakened Effect Size |
| *P* value | 0.009 | <0.05 | Statistical significance is maintained, with a possible slight increase |
| Publication Bias | / | Four studies were filled | Publication bias exists |
| **Incidence Rate-T2DM-CV-OR** | **Before Trim-and-Fill** | **After Trim-and-Fill** | **Significance of Changes** |
| Effect Size | 0.439(0.214, 0.663) | 0.352(0.119, 0.585) | Weakened Effect Size |
| *P* value | <0.001 | <0.05 | Statistical significance is maintained, with a possible slight increase |
| Publication Bias | / | Two studies were filled | Publication bias exists |
| **Incidence Rate-T1DM-SD-HR** | **Before Trim-and-Fill** | **After Trim-and-Fill** | **Significance of Changes** |
| Effect Size | -0.021(-0.475, 0.433) | 0.038(-0.396, 0.473) | Weakened Effect Size |
| *P* value | 0.036 | <0.05 | Statistical significance is maintained, with a possible slight increase |
| Publication Bias | / | One studies were filled | Publication bias exists |
