# Supplementary material for: Association between glycated hemoglobin variability and risk of diabetic kidney disease and diabetic retinopathy in diabetic patients: a systematic review and meta-analysis
Source: Front Endocrinol (Lausanne). 2026 Jan 30;17:1703190. doi: 10.3389/fendo.2026.1703190 (PMC12901347; doi:10.3389/fendo.2026.1703190)
Supplement: Supplementary file 6 [file Table2.docx]

**Appendix B**

**Table 1** Subgroup analyses for SD and CV of DR

| Subgroup | Studies | Pooled HR//OR  (95% CI) | *I^2^* (*p* value) |
| --- | --- | --- | --- |
| ***SD-T1DM-OR*** | | | |
| **Index** | | | |
| Adjusted SD | Rosa LCGFD et al.(2019) substudy1/  Rosa LCGFD et al.(2019) substudy2/  Suh J et al.(2023) substudy1/  Suh J et al.(2023) substudy2 | 7.52 (4.02, 14.05） | *I^2^* = 0%（*P* = 0.98） |
| SD | Virk SA et al.(2016) | 1.32 (1.00, 1.74） | */* |
| **Sample Size** | | | |
| ＜1000 | Rosa LCGFD et al.(2019) substudy1/  Rosa LCGFD et al.(2019) substudy2/  Suh J et al.(2023) substudy1/  Suh J et al.(2023) substudy2 | 7.52 (4.02, 14.05） | *I^2^* = 0%（*P* = 0.98） |
| ≥1000 | Virk SA et al.(2016) | 1.32 (1.00, 1.74） | */* |
| **Area** | | | |
| Non-Asia | Rosa LCGFD et al.(2019) substudy1/  Rosa LCGFD et al.(2019) substudy2/  Virk SA et al.(2016) | 3.90 (0.82, 18.49） | *I^2^* = 80%（*P* < 0.40） |
| Asia | Suh J et al.(2023) substudy1/  Suh J et al.(2023) substudy2 | 6.99 (3.32, 14.70） | *I^2^* = 0%（*P* = 0.85） |
| **Design** |  |  |  |
| prospective | Virk SA et al.(2016) | 1.32 (1.00, 1.74） | */* |
| retrospective | Rosa LCGFD et al.(2019) substudy1/  Rosa LCGFD et al.(2019) substudy2/  Suh J et al.(2023) substudy1/  Suh J et al.(2023) substudy2 | 7.52 (4.02, 14.05） | *I^2^* = 0%（*P* = 0.98） |
| ***SD-T2DM-HR*** | | | |
| **Index** | | | |
| Adjusted SD | Ma C et al.(2022) substudy1/  Ma C et al.(2022) substudy2/  Ma C et al.(2022) substudy3 | 0.88 (0.50, 1.54） | *I^2^* = 0%（*P* = 0.06） |
| SD | Wu TE et al.(2022) substudy1/  Wu TE et al.(2022) substudy2/  Teh XR et al.(2025)/  Cardoso CRL et al.(2018) substudy1/  Cardoso CRL et al.(2018) substudy2/  Hu J et al.(2021) substudy1/  Hu J et al.(2021) substudy2/  Hu J et al.(2021) substudy3 | 1.21 (1.07, 1.37) | *I^2^* = 73%（*P* ＜ 0.01） |
| **Design** | | | |
| prospective | Cardoso CRL et al.(2018) substudy1/  Cardoso CRL et al.(2018) substudy2/  Wu TE et al.(2022) substudy1/  Wu TE et al.(2022) substudy2/  Ma C et al.(2022) substudy1/  Ma C et al.(2022) substudy2/  Ma C et al.(2022) substudy3/  Hu J et al.(2021) substudy1/  Hu J et al.(2021) substudy2/  Hu J et al.(2021) substudy3 | 1.32 (1.00, 1.74） | *I^2^* = 68%（*P* < 0.01） |
| retrospective | Teh XR et al.(2025) | 1.20 (1.09, 1.32) | */* |
| **Sample Size** | | | |
| ＜2000 | Cardoso CRL et al.(2018) substudy1/  Cardoso CRL et al.(2018) substudy2/  Wu TE et al.(2022) substudy1/  Wu TE et al.(2022) substudy2 | 1.02 (0.92, 1.13） | *I^2^* = 0%（*P* = 0.89） |
| 2000-40000 | Ma C et al.(2022) substudy1/  Ma C et al.(2022) substudy2/  Ma C et al.(2022) substudy3/  Hu J et al.(2021) substudy1/  Hu J et al.(2021) substudy2/  Hu J et al.(2021) substudy3 | 1.41 (1.22, 1.63） | *I^2^* = 28%（*P* = 0.23） |
| ≧40000 | Teh XR et al.(2025) | 1.20 (1.09, 1.32) | */* |
| **Area** | | | |
| Non-Asia | Cardoso CRL et al.(2018) substudy1/  Cardoso CRL et al.(2018) substudy2 | 1.13（1.05-1.21） | *I^2^* = 0%（*P* = 0.63） |
| Asia | Wu TE et al.(2022) substudy1/  Wu TE et al.(2022) substudy2/  Ma C et al.(2022) substudy1/  Ma C et al.(2022) substudy2/  Ma C et al.(2022) substudy3/  Hu J et al.(2021) substudy1/  Hu J et al.(2021) substudy2/  Hu J et al.(2021) substudy3/  Teh XR et al.(2025) | 1.27 (1.11, 1.44） | *I^2^* = 56%（*P* = 0.02） |
| **Time** | | | |
| median of 5 years | Hu J et al.(2021) substudy1/  Hu J et al.(2021) substudy2/  Hu J et al.(2021) substudy3 | 1.45 (1.28, 1.65） | *I^2^* = 31%（*P* = 0.23） |
| ≧median of 5 years | Cardoso CRL et al.(2018) substudy1/  Cardoso CRL et al.(2018) substudy2/  Wu TE et al.(2022) substudy1/  Wu TE et al.(2022) /  Teh XR et al.(2025) | 1.09 (0.99, 1.20） | *I^2^* = 30%（*P* = 0.22） |
| **Quartile** | | | |
| Q2/Q1 | Hu J et al.(2021) substudy1/  Ma C et al.(2022) substudy1 | 1.25 (0.75, 2.08） | *I^2^* = 35%（*P* = 0.21） |
| Q3/Q1 | Hu J et al.(2021) substudy2/  Ma C et al.(2022) substudy2 | 1.13 (0.64, 2.01） | *I^2^* =45%（*P* = 0.18） |
| Q4/Q1 | Hu J et al.(2021) substudy3/  Ma C et al.(2022) substudy3 | 1.61 (1.36, 1.91) | *I^2^* =0%（*P* = 0.67） |
| ***CV-T1DM-HR*** |  |  |  |
| **Sample Size** |  |  |  |
| <1000 | Schreur V et al.(2018)/  Romero-Aroca P et al.(2021) substudy1/  Romero-Aroca P et al.(2021) substudy2 | 1.21 (0.96, 1.53） | *I^2^* = 67%（*P* = 0.05） |
| 1000-30000 | Hietala K et al.(2013) substudy1/  Hietala K et al.(2013) substudy2/  Hietala K et al.(2013) substudy3 | 1.35 (1.07, 1.70） | *I^2^* = 0%（*P=*0.42） |
| ≧30000 | Hermann JM et al.(2014) | 1.11 (1.10, 1.12) | */* |
| **Design** | | | |
| prospective | Hermann JM et al.(2014)/  Romero-Aroca P et al.(2021) substudy1/  Romero-Aroca P et al.(2021) substudy2 | 1.32 (1.00, 1.74） | *I^2^* = 52%（*P* = 0.13） |
| retrospective | Schreur V et al.(2018)/  Hietala K et al.(2013) substudy1/  Hietala K et al.(2013) substudy2/  Hietala K et al.(2013) substudy3 | 1.20 (1.09, 1.32) | *I^2^* = 84%（*P* <0.01） |
| **Area** |  |  |  |
| 南欧 | Romero-Aroca P et al.(2021) substudy1/  Romero-Aroca P et al.(2021) substudy2 | 1.40 (1.12, 1.76） | *I^2^* =0%（*P=* 0.88） |
| 北欧 | Hietala K et al.(2013) substudy1/  Hietala K et al.(2013) substudy2/  Hietala K et al.(2013) substudy3 | 1.50 (1.27, 1.78) | *I^2^* = 0%（*P=*0.42） |
| 中欧 | Hermann JM et al.(2014) | 1.11 (1.10, 1.12) | */* |
| 西欧 | Schreur V et al.(2018) | 1.05 (1.03, 1.08) | */* |
| ***CV-T1DM-OR*** |  |  |  |
| **Time** |  |  |  |
| **Design** | | | |
| prospective | Virk SA et al.(2016) | 1.31 (1.03, 1.67） | */* |
| retrospective | Rosa LCGFD et al.(2019) | 5.24 (1.60, 17.16） | */* |

SD: standard deviation; CV: coefficient of variation; HR: hazard ratio; OR: odd ratio.

**Table 2. Sensitivity analysis of the association between HbA1c variability and DR**

| Type analysis | Trial | Pooled HR/OR  (95% CI) | *I^2^* (*p* value) |
| --- | --- | --- | --- |
| ***SD-T1DM-OR*** | | | |
| Excluding trials | Virk SA et al.(2016) | 7.52 (4.02, 14.05） | *I^2^* = 0%（*P=*0.98） |
| ***SD-T2DM-HR*** |  |  |  |
| Excluding trials | Hu J et al.(2021) substudy3 | 1.15 (1.04, 1.26） | *I^2^* = 39%（*P* =0.10） |
| ***SD-T2DM-OR*** |  |  |  |
| Excluding trials | Song KH et al.(2019) | 0.98 (0.88, 1.08) | *I^2^* = 36%（*P* =0.20） |
| ***CV-T1DM-HR*** |  |  |  |
| Excluding trials with Sample Size <500 | Schreur V et al.(2018)/  Romero-Aroca P et al.(2021) substudy1/  Romero-Aroca P et al.(2021) substudy2 | 1.36 (1.14, 1.63)/  1.14 (1.07, 1.22)/  1.13 (1.07, 1.21) | *I^2^* = 73%（*P* <0.01)/  *I^2^* = 85%（*P* <0.01)/  *I^2^* = 84%（*P* <0.01) |
| Fixed model analysis | Hermann JM et al.(2014)/  Schreur V et al.(2018)/  Romero-Aroca P et al.(2021) substudy1/  Romero-Aroca P et al.(2021) substudy2 | 1.35 (1.10, 1.66)/  1.36 (1.14, 1.63)/  1.14 (1.07, 1.22)/  1.13 (1.07, 1.21) | *I^2^* = 80%（*P*＜0.01）/  *I^2^* = 73%（*P* <0.01)/  *I^2^* = 85%（*P* <0.01)/  *I^2^* = 84%（*P* <0.01) |
| ***CV-T2DM-HR*** |  |  |  |
| Excluding trials | Teh XR et al.(2025) | 1.11 (1.06, 1.16) | *I^2^* = 0%（*P* =0.57） |
| ***CV-T2DM-OR*** |  |  |  |
| Excluding trials | Dhatariya K et al.(2021) | 0.99 (0.98, 1.00) | *I^2^* = 0%（*P* =0.69） |

SD: standard deviation; CV: coefficient of variation; HR: hazard ratio; OR: odd ratio.
