# Supplementary material for: Association between glycated hemoglobin variability and risk of diabetic kidney disease and diabetic retinopathy in diabetic patients: a systematic review and meta-analysis
Source: Front Endocrinol (Lausanne). 2026 Jan 30;17:1703190. doi: 10.3389/fendo.2026.1703190 (PMC12901347; doi:10.3389/fendo.2026.1703190)
Supplement: Supplementary file 7 [file Table3.docx]

| **Metric** | **Computation** | **Meaning** | **Advantages** | **Limitations** |
| --- | --- | --- | --- | --- |
| HbA1c-SD | $\sqrt{\Sigma_{k=1}^{n}\frac{\left( x_{i}-\bar{x} \right)^{2}}{n-1}}$ | Fluctuation of HbA1c around the average value | Easy to compute | Clinical interpretation is not intuitive; Unable to distinguish short-term sharp fluctuations from long-term chronic trends; Unable to differentiate the direction of changes in fluctuations. |
| HbA1c-CV | $SD/\bar{X}$ | HbA1c variability around the mean | Easy to compute; Removing the effect of the mean | Clinical interpretation is not intuitive; Unable to distinguish short-term sharp fluctuations from long-term chronic trends; Unable to differentiate the direction of changes in fluctuations. |
| HbA1c-HVS | The number of HbA1c changes >0.5% over the total number of HbA1c measurements | Fluctuation frequency of HbA1c | Intuitive results;  Meets the actual clinical needs | Ignore the extent of change; Unable to differentiate the direction of changes in fluctuations; Affected by the timing and frequency of measurements. |
| HbA1c-HGI | Measured HbA1c minus predicted HbA1c from FBG levels | Difference between actual HbA1c and HbA1c predicted from FPG | Indicating influence of non-glycemic factors on HbA1c | Difficult to calculate |

**Appendix C**

**Table 1** Comparison of glycated hemoglobin variability indicators

n= total number of HbA1c measurements, $X_{i}$= serially measured HbA1c, and $\bar{X}=$ mean of HbA1c.
